# Contribution of Arbuscular Mycorrhizal Fungi, Phosphate–Solubilizing Bacteria, and Silicon to P Uptake by Plant

**DOI:** 10.3389/fpls.2021.699618

**Published:** 2021-07-01

**Authors:** Hassan Etesami, Byoung Ryong Jeong, Bernard R. Glick

**Affiliations:** ^1^Department of Soil Science, University of Tehran, Tehran, Iran; ^2^Department of Horticulture, Division of Applied Life Science (BK21+ Program), Graduate School, Gyeongsang National University, Jinju, South Korea; ^3^Department of Biology, University of Waterloo, Waterloo, ON, Canada

**Keywords:** phosphorus availability, silicon fertilizer, silicate solubilization, silicate-solubilizing bacteria, synergistic interactions

## Abstract

Phosphorus (P) availability is usually low in soils around the globe. Most soils have a deficiency of available P; if they are not fertilized, they will not be able to satisfy the P requirement of plants. P fertilization is generally recommended to manage soil P deficiency; however, the low efficacy of P fertilizers in acidic and in calcareous soils restricts P availability. Moreover, the overuse of P fertilizers is a cause of significant environmental concerns. However, the use of arbuscular mycorrhizal fungi (AMF), phosphate–solubilizing bacteria (PSB), and the addition of silicon (Si) are effective and economical ways to improve the availability and efficacy of P. In this review the contributions of Si, PSB, and AMF in improving the P availability is discussed. Based on what is known about them, the combined strategy of using Si along with AMF and PSB may be highly useful in improving the P availability and as a result, its uptake by plants compared to using either of them alone. A better understanding how the two microorganism groups and Si interact is crucial to preserving soil fertility and improving the economic and environmental sustainability of crop production in P deficient soils. This review summarizes and discusses the current knowledge concerning the interactions among AMF, PSB, and Si in enhancing P availability and its uptake by plants in sustainable agriculture.

## Introduction

There is a growing need to improve food production to meet the requirements of the increasing world population. This may be done in either of two ways: increasing the area under cultivation or enhancing the yield per unit area. The former is not possible in many countries of the world due to a number of restrictions including the availability of water or soil resources, climate change, drought, and soil salinization (Etesami and Noori, 2019). On the other hand, one of the ways to increase the yield per unit area is to improve the nutritional properties of the soil. As an essential plant nutrient, P is required for carbon metabolism, energy generation, energy transfer, enzyme activation, membrane formation, and nitrogen (N_2_) fixation (Schachtman et al., 1998). P also forms key biological molecules like ATP, nucleic acids, and phospholipids (Marschner, 1995). P deficiency is a significant limiting factor for the growth and yield of crops that affects approximately 50% of all agricultural ecosystems around the world (Lynch, 2011; Ringeval et al., 2017; Etesami, 2020). To address this issue, there has been an enormous worldwide increase in the use of P fertilizers. The high agricultural P demand has put the sustainability of P mining for fertilizer production into question (Elser, 2012). P fertilizers often lead to the addition of a large excess of P in agricultural soils. Unfortunately, >80% of the P fertilizers applied to the soil is lost due to adsorption and fixation processes (de La Vega et al., 2000; Vance et al., 2003) or it is transformed into organic forms (Holford, 1997), which represent 40–80% of total soil P (Bünemann et al., 2010), with phytates as the most common form (Menezes-Blackburn et al., 2014). Therefore, the availability of this added P to plants is limited (about 0.1% of the total P).

P is usually absorbed by the plant in a limited range of soil conditions, i.e., pH 6.5–7 as H_2_PO_4_^–^ and HPO_4_^2–^. When the soil pH exceeds 7.0, inorganic phosphate (Pi) is predominantly mineralized and immobilized as calcium phosphates. At lower soil pH levels, P is usually bound/adsorbed by soluble aluminum (Al), iron (Fe), manganese (Mn), or the associated hydrous oxides (Brady and Weil, 1999). At neutral pH, Pi adsorbs to weathered silicates such as clay minerals (Rajan, 1975). Thus, the P concentration in soils with pH < 6.5 or pH > 7 is suboptimal, and is generally about 1–10 μM (Schachtman et al., 1998), which can result in crop yield depressions of 5–15% (Shenoy and Kalagudi, 2005).

The theoretical increase in plant growth efficiency from adding chemical P fertilizers has peaked so that additional chemical P fertilization cannot be expected to significantly increase plant yield (Etesami, 2020). Twenty-two million tons of P (3–4% of the total P demand) are annually extracted from natural sources (i.e., non-renewable phosphate rocks), according to the US geological survey (Gaxiola et al., 2011), which puts the natural P sources in risk of depletion (Cordell et al., 2009). Therefore, a more efficient use of P is needed, including maximizing P acquisition and utilization efficiencies (Veneklaas et al., 2012).

Some plants can efficiently acquire and/or use P to maintain metabolism and growth (Lambers et al., 2010; Aziz et al., 2014). Some plant mechanisms for improving P acquisition efficiency include (Ramaekers et al., 2010; Johri et al., 2015): (i) increased expression of high affinity P transporters; (ii) soil exploration at a minimal metabolic cost; (iii) topsoil foraging; (iv) stimulation of root hair growth; (v) redistribution of growth among root types; (vi) increase of the root-to-shoot ratio; (vii) the secretion of organic acids (e.g., citrate, malate, or oxalate) from roots to the soil; (viii) the activation of an advanced bio-molecular system; and (xi) enhanced acid phosphatase (rAPase) or phytase secretion.

Plants have also developed some biotic interactions with diverse soil microorganisms that promote plant growth. Arbuscular mycorrhizal fungi (AMF) and plant growth-promoting bacteria (PGPB) are the most common such microorganisms. AMF and PGPB, and especially the phosphate-solubilizing bacteria subgroup (PSB), are known to help overcome P deficiency in plants. PSB and AMF are a part of the key biogeochemical cycling processes (Sharma et al., 2013; Etesami, 2020).

Phosphate–solubilizing bacteria exist in most soils (Rodrìguez and Fraga, 1999). In *in vitro* conditions, they can improve P bioavailability by lowering the soil pH, solubilizing Pi, activating synthesized phosphatases, mineralizing organic P, and/or chelating P from Al^3+^, Ca^2+^, and Fe^3+^ (Rodrìguez and Fraga, 1999; Browne et al., 2009; Sharma et al., 2013; Etesami, 2020). Nearly all soils also contain AMF, which associate with approximately 80% of all plant roots (Smith and Read, 2008; Brundrett and Tedersoo, 2018). The ability of AMF to promote plant growth and yield and enhance P uptake has been well documented (Miransari, 2010; Jansa et al., 2011; Smith et al., 2011; Smith and Smith, 2011; Nadeem et al., 2014; Brundrett and Tedersoo, 2018; Etesami, 2020).

As a consequence of variable soil conditions, microorganisms may change crop productivity. Climate change also has a substantial impact on the effectiveness of microorganisms. One way to increase the efficiency of microorganisms under adverse environmental conditions is the co–inoculation of microorganisms (Nadeem et al., 2014; Etesami et al., 2015b; Etesami and Maheshwari, 2018; Ghorchiani et al., 2018) that stimulates plant growth through various mechanisms (Bashan et al., 2004). AMF and PGPB can work together to yield sustainable plant growth in malnourished environments (Zarei et al., 2006; Mohamed et al., 2014; Nadeem et al., 2014; Lee et al., 2015; Xun et al., 2015). Combinations of AMF and PGPB are commonly used to increase crop yields (Mäder et al., 2011; Ghorchiani et al., 2018), improve fruit quality (Ordookhani et al., 2010; Bona et al., 2016), boost phytoremediation, enhance the fertilizer nutrient use efficiency (Xun et al., 2015), lower chemical fertilization application requirements (Adesemoye et al., 2009), and increase salinity tolerance (Gamalero et al., 2009).

The use of silicon (Si) fertilizer has also been proposed as an environmentally friendly, ecologically compatible method of improving plant growth and the resistance to multiple environmental stresses including nutritional imbalances (Etesami and Jeong, 2018, 2020; Etesami et al., 2020). Previous studies have reported that Si increases plant uptake of P (Kostic et al., 2017; Neu et al., 2017; Rezakhani et al., 2019a, b; Schaller et al., 2019). Combining Si and microorganism applications has been proposed to effectively induce improved plant growth and nutrition (Etesami, 2018; Etesami and Adl, 2020). Previous studies have observed that AMF and Si work together to improve plant growth regardless of the stress conditions (Hajiboland et al., 2018; Moradtalab et al., 2019), and that PSB and Si synergistically help plants better uptake P (Rezakhani et al., 2019a, b). However, how AMF, PSB and Si interact to affect P availability for plants is poorly understood. Thus, a better understanding of the interactions of AMF, PSB and Si would allow growers to rely less on chemical P fertilizers and instead utilize biological processes to maintain fertility and enhance plant growth. Hence, this review discusses the mechanisms which AMF, PSB, and Si, individually and together, use to increase plant uptake of P in agricultural systems where proper nutrition might otherwise suggest heavy use of P fertilizers. This review also highlights future research needs regarding how to improve plant uptake of P using AMF, PSB, and Si. In addition, the role of silicate-solubilizing bacteria (SSB), which convert insoluble silicate forms to available forms for the plant, in increasing P and Si availability and their uptake by plants is discussed.

## Plant Responses to P Scarcity

Plants exhibit a complex array of biochemical, morphological, and physiological adaptations to deal with P deficiency, which are generally known as “P starvation responses” (Plaxton, 2004) and endeavor to increase P acquisition capacity and to preserve plant vitality (Pang et al., 2015). Some P deficiency responses are as follows. A preferential carbohydrate allocation toward the roots, higher density of root hairs, greater root surface area and length, as well as root cluster formations alter the root structure and lead to reduced plant growth and increased root-to-shoot ratio (Gilroy and Jones, 2000; Liao et al., 2001; Sánchez-Calderón et al., 2006; Lynch, 2007; Niu et al., 2013; Aziz et al., 2014; Lambers and Plaxton, 2015). The greater surface area provided by the larger root system allows for better absorption of nutrients, including P, through increased contact with the soil (Römer and Schenk, 1998; López-Bucio et al., 2003; Lynch, 2007). Another important response to P deficiency is an increase in the root organic acid exudations, i.e., carboxylates (mainly citrate and malate) to the rhizosphere to increase the rhizospheric inorganic P availability (Neumann and Römheld, 1999; Vance et al., 2003; Raghothama and Karthikeyan, 2005; Johnson and Loeppert, 2006; Pang et al., 2015). Plants also exhibit an increased efficiency of cellular P uptake. Inorganic P in soils is generally very immobile, so that the uptake of rhizospheric Pi is affected by the high–affinity Pi/H^+^ symporters associated to the plasma membranes that belong to the *PHT1* gene family (Gu et al., 2016). Previous studies observed that P deficiency induced the expression of Pi transporters in wheat (Gilroy and Jones, 2000; Tittarelli et al., 2007; Miao et al., 2009; Jia et al., 2011; Kostic et al., 2015; Kostic et al., 2017). Plants also induce enzymes that scavenge and recycle Pi, such as acid phosphatase, which catalyzes Pi hydrolysis from Pi–monoesters; nuclease, which degrades extracellular DNA and RNA; and phosphodiesterase, which liberates Pi from nucleic acids (Duff et al., 1994; Plaxton and Carswell, 1999; Gaume et al., 2001; Plaxton, 2004). Plants may also induce alternate cytosolic glycolysis pathways (Plaxton and Carswell, 1999), tonoplast pyrophosphatase that pumps H^+^, and different respiratory electron transport pathways (Gonzàlez-Meler et al., 2001; Plaxton, 2004). Plants also remobilize the internal P from one plant part to another (Gill and Ahmad, 2003). Plants modify the carbohydrate partitioning between source and sink, photosynthesis, sugar metabolism in response to P deficiency (Sánchez-Calderón et al., 2006), the cations in carbon metabolism and alternate respiratory pathways (Uhde-Stone et al., 2003), and/or membrane biosynthesis to require lower amounts of P (Uhde-Stone et al., 2003; Lambers et al., 2006). Moreover, plants establish mycorrhizal symbioses, beneficial associations between soil fungi and plant roots (Smith and Read, 2008).

## AMF and Their Mechanisms of P Uptake/Mobilization

Arbuscular mycorrhizae are endomycorrhiza where the fungal hyphae penetrate the root cell walls and get in touch with the plasmalemma. AMF are commonly found in all of earth’s ecosystems with plants (Redecker et al., 2013). The formation of arbuscular mycorrhiza has allowed plants to survive and grow in natural habitats for millions of years without fertilizers, pesticides and irrigation. AMF belong to the subphylum Glomeromycotina (Bruns et al., 2018), encompassing 340 described species^1^.

Having evolved 400–450 million years ago, this symbiosis is likely the oldest type of mycorrhiza, and it involves a wide variety of plants (Smith and Read, 2008). AMF are obligate symbionts and acquire all of their organic carbon requirements from their plant partners. The symbiosis is often mutualistic based largely on carbon exchange from the plant (4–20% of photosynthetically fixed carbon) and P delivered by the fungi (Wright et al., 1998; Smith and Smith, 2011). More than 80% of earth’s plant species are estimated to be able to form this mycorrhizal symbiosis (Wang and Qiu, 2006). The benefits of the arbuscular mycorrhizae in various plants (mostly in crops) have been proven (Smith and Read, 2008). AMF increase plant resistance to abiotic stresses, improve mineral uptake (particularly of P), enhance water relations, and provide protection against soil-borne pathogens to promote plant growth (Smith and Read, 2008). On top of significantly aiding the P supply to plants, AMF can help plants acquire macronutrients and micronutrients like Cu, K, Mg, N, and Zn, especially when they’re present in less soluble forms in soils (Meding and Zasoski, 2008; Smith and Read, 2008). These fungi penetrate the root cortical cell walls and establish arbuscules, which are haustoria–like structures, that mediate the metabolite exchanges between the host cell and the fungi (Oueslati, 2003). AMF enhance the root zone absorption area by 10–100% and improve the plant ability to utilize more soil resources. Mycorrhizal roots are able to reach a greater soil volume than non-mycorrhizal ones, thanks to the extraradical hyphae that facilitate the nutrient absorption and translocation (Smith and Read, 2008). AMF increase the nutrient absorption by increasing the absorption area of the roots, and also release chemicals such as glomalin, a glycoprotein secreted by hyphae and spores of AMF. Glomalin in the soil aids the uptake of nutrients such as Fe and P that are difficult to dissolve (Smith and Read, 2008; Miransari, 2010; Emran et al., 2017; Begum et al., 2019). P is easily absorbed from soil particles and therefore Pi-free zones are readily formed around the roots. Extraradical hyphae of the mycorrhizal roots extend beyond these P-depleted zones, taking up the bio-available Pi that is otherwise inaccessible to plants.

The roots of arbuscular mycorrhizal plants have two pathways to absorb P. The first pathway is common to both arbuscular mycorrhizal plants and non-arbuscular mycorrhizal plants, where P is directly absorbed from the root epidermis and hairs. The second pathway involves P entering the root cortical cells (intraradical mycelium) (Smith and Smith, 2011), where symbiotic interfaces are provided by arbuscules or hyphal coils, through the fungal hyphae (P uptake from the interfacial apoplast by cell-specific Pi transporter gene expression in the mycorrhizal roots) (Benedetto et al., 2005; Balestrini et al., 2007; Gomez-Ariza et al., 2009; Tisserant et al., 2012; Fiorilli et al., 2013). This is a rapid P translocation over many centimeters. New physiological and molecular evidence suggests that for P, regardless of plant growth responses, the mycorrhizal pathway is operational (Smith and Smith, 2011). The function of the transporters and the translocation of Pi in the fungi and the transfer of Pi to the host plants have been well reviewed (Johri et al., 2015; Ezawa and Saito, 2018).

As mentioned above, the low solubility of P in acidic and alkaline soils (e.g., lower than 10 μM) results in a very low mobility (Schachtman et al., 1998). Therefore, when P is absorbed by the roots, its replacement from bulk soil is very slow, which leads to the establishment of P-depletion zones, where all the available P has been utilized quickly from around the roots, thereby reducing P uptake by the root epidermis hairs (the first pathway of P absorption) (Schachtman et al., 1998; Smith and Smith, 2011). Therefore, for improved P acquisition, plants must overcome these depletion areas and display root activities in other parts of the soil. The result of this effort for P (and other relatively immobile soil resources) acquisition is determined by the root system surface area. The most important role of the hyphae in mycorrhizal fungi is the increase of the root surface area (depletion is lower around small–diameter arbuscular mycorrhizal fungal hyphae) (Smith and Smith, 2011). In addition, mycorrhizal plants are able to exude organic acids such as citrate and malate that chelate Al^3+^ (Klugh and Cumming, 2007; Klugh-Stewart and Cumming, 2009) and Ca^2+^ and dissolve aluminum and calcium phosphates. By enhancing the soil contact area through AMF hyphae, plants are granted improved access to Pi and orthophosphates in the soil solution (Bouhraoua et al., 2015), as the roots are able to directly take up the released Pi with the help of arbuscular mycorrhizal fungal hyphae. Arbuscular mycorrhizal roots do not establish a fungal sheath, and theoretically are able to use both of the nutrient uptake pathways. It has been proposed that the two nutrient uptake pathways act additively in the arbuscular mycorrhizal symbiosis (Bücking et al., 2012). However, approximately 80% of P uptake in a mycorrhizal plant is estimated to be supplied by the fungi (Marschner and Dell, 1994). AMF also increase the ability of legumes to fix N_2_ and reduce the amount of inorganic N that leaches (Veresoglou et al., 2012). Nitrogen is a component of chlorophyll and thus is important for photosynthesis. The transfer of photosynthetic materials to the roots results, in turn, in increased activity of soil microorganisms including AMF and PSB.

In general, AMF can increase P uptake in P-deficient soils by (i) increasing the P uptake rate (P influx) per unit of arbuscular mycorrhizal root. This increased P uptake rate with AMF is due to the high effectiveness with which hyphal surfaces absorb P from the soil, compared to the cylindrical root surfaces (Sharif and Claassen, 2011); (ii) expanding the mycorrhizal hyphal network to reach beyond the rhizosphere, absorbing Pi by AMF hyphae via fungal Pi transporters up to 25 cm around the roots, translocating the Pi to intracellular fungal structures in the root cortical cells (Smith et al., 2011; Garg and Pandey, 2015); (iii) storing P in the form of polyphosphates, such that the fungi can keep the internal Pi levels relatively low, effectively transferring P from soil to plant-based hyphae through appressoria and from the extraradical mycelium to the intraradical mycelium (Pepe et al., 2020); (iv) having hyphae with a small diameter (2–20 μm) that allow the fungi to access small soil cores for P, and achieve greater P influx rates for a given surface area (Jakobsen et al., 1992; Jakobsen et al., 2001); and (v) decreasing the depletion zone around the roots or hyphae (decreasing the impact of rhizospheric Pi depletion) (Smith et al., 2011; Garg and Pandey, 2015). In one study, P depletion around the roots of *Capsicum annuum* L. plants or the hyphae of *Glomus mossea* only extended to about 0.06 cm and thus only ∼7% of the soil P was positionally available to the roots. But for the hyphae it was ∼100%, of the soil was positionally available because the half distance between neighboring hyphae was only 0.01 cm (Sharif and Claassen, 2011). As a general conclusion, the high effectiveness of hyphal surfaces to absorb P from soils may be enough in most cases to explain how AMF improve the uptake of available P from the soil.

## PSB and Their Mechanisms of P Uptake/Mobilization

Rhizospheric P mineralization and solubilization are important mechanisms by which PSB increase the nutrient availability for plants (Glick, 2012). PSB play a major role in all three main parts of the soil P cycle (dissolution–precipitation, mineralization–immobilization, and sorption–desorption). There are various mechanisms by which PSB can change the insoluble phosphates into available forms (Gyaneshwar et al., 2002; Khan et al., 2007; Sharma et al., 2013; Etesami and Maheshwari, 2018; Etesami, 2020). PSB strains belong to various genera (e.g., *Achromobacter*, *Actinomadura*, *Aerobactor*, *Agrobacterium*, *Alcaligenes*, *Arthrobacter, Azotobacter*, *Azospirillum*, *Bacillus*, *Chryseobacterium*, *Delftia*, *Enterobacter*, *Gordonia*, *Klebsiella*, *Pantoea*, *Phyllobacterium*, *Pseudomonas*, *Rhizobium*, *Rhodococcus*, *Serratia*, *Streptomyces, Thiobacillus*, *Xanthobacter*, *Xanthomonas*) (Sharma et al., 2013; Etesami, 2020) and can solubilize insoluble Pi compounds including dicalcium phosphate, hydroxyapatite, tricalcium phosphate, and rock phosphate, and mineralize organic phosphate compounds to forms that can be absorbed by plants (i.e., H_2_PO_4_^–^ and HPO_4_^2–^) (Khan et al., 2009; Ramaekers et al., 2010; Alori et al., 2017; Etesami, 2020). Each phosphate–solubilizing bacterium may employ multiple mechanisms to solubilize insoluble P. Some of the most significant bacterial mechanisms that increases P availability for plants are briefly discussed in the following sections.

### Production of Organic Acids

**FIGURE 1:**
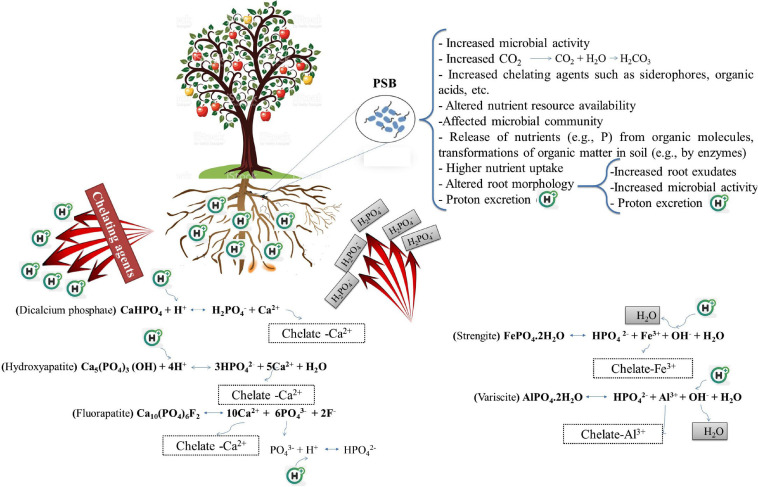
The part that phosphate solubilizing bacteria (PSB) play to improve plant’s ability to acquire the soil phosphorus by altering the sorption equilibria that may increase the net orthophosphate ion transfer into the soil solution. Protons and metal chelating agents are especially effective in solubilizing precipitated phosphorus forms (aluminum phosphates and iron phosphates in acidic conditions, and calcium phosphates in alkaline conditions). Le Chatelier’s principle states that increasing a certain substance’s concentration leads to the balance shifting such that consumption of that substance increases, and lowering the concentration of a material promotes the balance toward the production of the said material. Chelating agents (organic anions, siderophores, etc.) remove Al^3+^, and Ca^2+^, and Fe^3+^ ions from the reaction and cause the balance to shift such that more H_2_PO_4_^–^ and HPO_4_^2–^ are produced. The addition of H^+^ ion also causes the balance to be adjusted to encourage the reduction of H^+^ ions, leading to more H_2_PO_4_^–^ and HPO_4_^2–^ to be produced.

Most P uptake occurs in the pH range 6.5–7. However, because of equilibrium reactions such as sorption/desorption and the dissolution of P-bearing minerals are pH-dependent, PSB solubilize Pi in neutral to alkaline soils by excreting protons and producing organic and inorganic acids (Farhat et al., 2009; Jones and Oburger, 2011). NH_4_^+^ assimilation by plants and PSB leads to hydrogen ion (H^+^) excretion to maintain electroneutrality (Parks et al., 1990; Wu et al., 2008). Organic acids (e.g., 2–ketogluconic, aspartic, citric, gluconic, lactic, malic, malonic, oxalic, succinic, and tartaric acid) are produced by bacterial metabolism, mainly due to oxidative respiration or carbon source fermentations, such as periplasmic glucose oxidizing into gluconic acid and being released into the soil solution, or the oxidation of organic matter or animal fertilizers added to the soil (Gyaneshwar et al., 2002; Trolove et al., 2003; Goldstein, 2007; Jones and Oburger, 2011). Organic acids can solubilize P from mineral surfaces via ligand-promoted dissolution or ligand exchange (Jones and Oburger, 2011; Oburger et al., 2011). In addition, PSB can indirectly reduce the pH of the rhizosphere and increase P levels by affecting the root system and, consequently, increasing the root exudates. Since root exudates contain different chelating agents and organic acids, they can increase the rhizospheric P availability. Organic acids (or organic anions) can enhance the rhizospheric P levels by lowering the pH, as PSB generally release the dissociated organic acids with protons, which allows them to preserve electroneutrality (Whitelaw et al., 1999; Castagno et al., 2011; Jones and Oburger, 2011). Organic acids compete with phosphates for fixation sites, or even replace the adsorbed phosphates on the soil clays surfaces, such as amorphous aluminum oxides, goethite, kaolinite, and montmorillonite. Chelating agents present in the root exudates (e.g., siderophores) can improve P availability to plants by promoting the chelation of P-bound Al^3+^, Ca^2+^, and Fe^3+^, or establishing soluble complexes with metal ions associated with insoluble P, which circumvents Pi precipitation (Figure 1) (Whitelaw, 1999; Rashid et al., 2004; Osorio Vega, 2007). On the other hand, root exudates come from different carbon sources (e.g., amino acids, mucilage, nucleotides, organic acids, phyto–siderophores, sugars, and vitamins) and have different signals, which lead to the attraction of microbial flora at the root level, including PSB. Increases in the microbial population result in the production of more rhizospheric organic acid production and subsequently decreases the rhizospheric pH (Khan et al., 2007; Pothier et al., 2007; Badri and Vivanco, 2009; Drogue et al., 2013; Sharma et al., 2013; Etesami et al., 2015b; Etesami, 2020; Figure 1).

### Production of Inorganic Acids

Mineral acids like carbonic acid (H_2_CO_3_), hydrochloric acid (HCl), nitric acid (HNO_3_), and sulfuric acid (H_2_SO_4_), in addition to organic acids, have been reported to contribute to solubilizing insoluble Pi (Sharma et al., 2013). Sulfur–oxidizing bacteria (SOB) such as those belonging to the genus *Thiobacillus* and nitrifying bacteria (NB) like those belonging to the genera *Nitrosomonas* and *Nitrobacter*, oxidize sulfur and ammonia and lead to the formation of inorganic acids and, consequently, reduce the pH, which ultimately increases the rhizospheric P availability (Stamford et al., 2003). SOB oxidize reduced sulfur compounds to produce sulfuric acid in the presence of oxygen to obtain energy while NB get their energy by oxidizing inorganic nitrogen compounds. Carbon dioxide (CO_2_) resulting from microbial respiration and organic matter decomposition, after combining with water, becomes carbonic acid which can also reduce the rhizospheric pH and lead to increased P availability (Figure 1). In general, the role of inorganic acids in the solubilization of P is lower than that of organic acids and is less frequently reported. Since the ability of PSB to lower the pH in certain instances is not always associated with Pi solubilization ability, acidification cannot be the only mechanism for dissolving insoluble Pi (Bashan et al., 2013).

### Siderophore Production

Plants and microorganisms in low-iron conditions produce siderophores which are low molecular weight (200–2000 Da) organic compounds with an iron–chelating ability (Ahmed and Holmström, 2014). The primary role of siderophores is to chelate Fe(III) under various environmental conditions making the element available to plants and microorganisms. Siderophores can bind to a variety of metals besides Fe(III) including Al, Ca, Cd, Co, Cu, Mn, Mo, Ni, Pb, and Zn, albeit with a lower affinity (Ahmed and Holmström, 2014). PSB have also been shown to be capable of producing siderophores (Vassilev et al., 2006; Caballero-Mellado et al., 2007; Hamdali et al., 2008; Karimzadeh et al., 2020) which can promote the dissolution of insoluble mineral P (Sharma et al., 2013). Siderophores can improve P availability for plants by ligand exchange and chelating the elements (e.g., Al^3+^, Ca^2+^, and Fe^3+^) that form a complex with P (Figure 1).

### Indole–3–Acetic Acid (IAA) and ACC-Deaminase Production

One mechanism that plants employ to deal with P deficiency is to allocate a large portion of the photosynthetic substrates to root growth, to develop fine roots with small diameters with greater surface area. Fine roots, especially root hairs, are associated with scavenging soil P with their high surface area (Rengel and Marschner, 2005). PGPB, including PSB, can improve a plant’s P capturing capacity by promoting root growth through branching, hormonal stimulation, or root hair development (phytostimulation; e.g., production of IAA or enzymes that modify plant ethylene precursors, like 1-aminocyclopropane 1-carboxylic acid (ACC) deaminase) (Richardson et al., 2009; Hayat et al., 2010; Emami et al., 2019). A plant’s response to P starvation stress can result in a decrease in the number of root hairs (Borch et al., 1999). The ACC deaminase enzyme can degrade the precursor for the ethylene production and influences how P affects the root growth; ethylene can adjust the root architectural response to soil P availability (Etesami and Maheshwari, 2018).

The abundance and length of the root hairs are positively correlated with the immobile element uptake. Modified root morphology of inoculated plants may enhance P uptake (Rengel and Marschner, 2005). Many PSB genera in soils are known to secrete IAA (Ahemad and Khan, 2010; He et al., 2010; Ahemad, 2012; Misra et al., 2012; Oves et al., 2013; Etesami and Alikhani, 2016a, b; Etesami and Maheshwari, 2018; Emami et al., 2019; Karimzadeh et al., 2020) that plant roots absorb, leading to increased endogenous pool of IAA in plants (Glick et al., 2007). In addition, many PSB are also reported to produce ACC deaminase (Iqbal et al., 2012; Sarathambal and Ilamurugu, 2013; Etesami et al., 2014; Shahzad et al., 2014; Etesami and Alikhani, 2016a; Karimzadeh et al., 2020).

Bacterial IAA can promote the development (architecture, branching, etc.) of the root system and increase root exudation. Organic acids in root exudates lead to rhizosphere acidification (Dakora and Phillips, 2002; Amir and Pineau, 2003; Jones et al., 2003) and also play an important part in forming and increasing the mobility of complexes with essential ions for plant uptake (Figure 1; Etesami et al., 2015a). For example, Hinsinger (2001) reported the role of exuded carboxylates in solubilizing various P complexes. Exuded organic acid anions may also be the growth substrates for microorganisms. Root exudates are a more effective nutrient source than soil organic matter that are easily degradable for microorganisms in the rhizosphere (Rengel and Marschner, 2005).

The increase in CO_2_ production from respiration of the rhizosphere microbial population leads to acidification of the rhizospheric environment. This can also lead to enhanced P availability, by increasing the release of new root extracts. Rhizospheric acidification also results from the H^+^ pump from plant and microbe nutrient uptake, N_2_ fixation by the symbiosis between *Rhizobium* and legume, and organic matter decomposition (Marschner and Rimmington, 1988). Certain microorganisms may indirectly enhance P nutrition for plants by enhancing root growth or root hair elongation, which allows for a greater degree of soil exploration instead of directly increasing soil P availability. IAA–producing PSB can also solubilize insoluble Pi in a manner similar to PSB by increasing the root surface area and subsequently increasing the root exudates (Dobbelaere et al., 1999; Lambrecht et al., 2000; Steenhoudt and Vanderleyden, 2000; Etesami and Maheshwari, 2018; Emami et al., 2019). In general, plant growth regulators influence root architecture and can increase P acquisition efficiency, especially from unavailable forms, and for this purpose root traits are a key factor (Campos et al., 2018).

### Organic P Mineralization

Organic P forms a significant part (40–80%) of the total soil P. Plants encountering P deficiency increase the exudation of P–hydrolyzing enzymes. In addition to dissolving phosphates affected by organic acids, the reactions of the phosphatase group of enzymes in the soil are also important. Phosphatases play a significant part in the organic P mineralization in soils. PSB can mineralize organic P by secreting phosphatases (Khan et al., 2009; Sharma et al., 2013; Etesami, 2020). Microbial–derived phosphatases are more likely to be combined with phosphate compounds than plant phosphatases are, and they help release orthophosphates from soil organic P (Tarafdar et al., 2001). Phytate (inositol hexaphosphate) is one of main soil organic P forms, accounting for over 50% of the total soil P (Osborne and Rengel, 2002). Phosphatases are not effective in mineralizing phytate. Phytase secreted by microorganisms converts phytate into P esters that can be broken down into Pi by phosphatases (Rengel and Marschner, 2005). Inorganic P immobilization by PSB can indirectly help P solubilization. PSB remove and assimilate P from the liquid culture medium according to the sink theory, activating the indirect dissolution of apatite or Ca_3_(PO_4_)_2_ (Illmer et al., 1995; Guidry and Mackenzie, 2003). This can be explained according to Le Chatelier’s principle, which states that lowering the concentration of Pi in soil solution promotes the balance toward the production of the Pi (e.g., release of Pi from calcium phosphates). Over a long period of time, all of the microbial P can potentially become available to plants. P immobilization in the biomass has been suggested to be an important mechanism for regulating P supply in a soil solution (Seeling and Zasoski, 1993), and for keeping labile P forms protected from reactions with the soil (Olander and Vitousek, 2004).

## Si and Its Role in P Uptake/Mobilization

Elemental Si is the second most abundant element in the lithosphere (approximately 28%). Si dioxide (SiO_2_) is the most common form of Si in soils. The main Si components in most soils includes amorphous silica, feldspars, kaolin, orthoclase, plagioclase, quartz, smectite, and vermiculite (Sahebi et al., 2015). Most Si contained in silicate minerals, and only a very small portion of the Si found in nature is available for use by plants (Struyf et al., 2010). The soluble Si is dependent on the pH and redox potential of the soil (Ma and Takahashi, 2002). In soils, Si is found as amorphous Si (minerogenic silica nodules, biogenic phytoliths, etc.), dissolved Si (adsorbed to aluminum or iron oxides/hydroxides or free in the soil solution), crystalline Si (primary silicates like feldspars, mica, quartz and secondary silicates like clay minerals), and poorly crystalline Si (e.g., secondary quartz) (Sauer et al., 2006). The soil soluble Si levels in ecosystems can differ up to two orders of magnitude (0.01–2.0 mM) (Haynes, 2014), and is mainly dependent on the parent material, soil diagenesis stage, and vegetation type (Derry et al., 2005; Struyf and Conley, 2009).

Si is not identified as an essential nutrient for plant growth and development. However, an increasing number of studies indicate that Si is a quasi-essential nutrient and is beneficial to plants, especially when under different stresses such as drought, heavy metal toxicity, nutritional imbalance, plant pathogens, and salinity; Si is also known to play an important part in plant ecology and evolution (Etesami and Jeong, 2018). Plant roots absorb the Si present as silicic acid [Si(OH)_4_] at levels of 0.1–0.6 mM in the soil solution, and pass it through the plasma membrane via two Si transporters, *Lsi1* and *Lsi2*, that respectively function as the influx and efflux transporters and have been identified in barley, pumpkin, rice, and wheat (Ma et al., 2006, 2007; Chiba et al., 2009; Mitani-Ueno et al., 2011; Montpetit et al., 2012). Si is polymerized to silica gel (SiO_2_⋅nH_2_O) in plants, generally referred to as silica bodies or phytoliths, which are released back into the soil as dead plant materials that decay and then may be taken up by plants (Carey and Fulweiler, 2012). Si is customarily found as hydrogen-bound bound organic Si complexes in plant tissues (Carlisle et al., 1977) and saturates the walls of the epidermis and vessels (Kaufmian et al., 1969) where it strengthens plant tissues and reduces water transpiration.

Si levels in the aboveground plant parts differ greatly depending on the plant species, accounting for 0.1–10.0% of the dry weight, and are often at concentrations similar to that of essential macronutrients such as K, N, and P (Epstein, 1999). Plants take up Si actively via metabolically–driven transporters, or passively or rejectively, with water (Mitani and Ma, 2005). The disparity in the Si accumulation of different crop species is due to the difference in the Si absorbing capacity of the roots. Generally, monocots are considered good Si accumulators, where Si concentrations are greater than 1% of the dry weight, whereas most dicots accumulate Si at levels lower than 0.1% of the total biomass and are considered excluders (Guntzer et al., 2012).

Si also influences the uptake of micronutrients and macronutrients in plants (Etesami and Jeong, 2018; Greger et al., 2018). Si fertilization increases P levels in different crops and improves plant growth by enhancing P availability for plants (Gladkova, 1982; Jianfeng and Takahashi, 1991; Singh and Sarkar, 1992; Owino-Gerroh and Gascho, 2005; Kostic et al., 2017; Neu et al., 2017; Reithmaier et al., 2017; Etesami and Jeong, 2018; Rezakhani et al., 2019a, b; Schaller et al., 2019). For example, Greger et al. (2018) found that Si increased the soil P availability by up to 50%. Kostic et al. (2017) also observed that Si supplied as Na_2_SiO_3_ increased P levels in the shoots of wheat grown in low P acid soil (available *P* < 4 mg kg^–1^ and pH 4.0) to an adequate level (>0.3%) in the range of P-fertilized wheat under greenhouse conditions. In this study, Si application increased the root organic acid exudation, such as malate and citrate that mobilize the rhizospheric Pi and up-regulate expression of Pi transporters (*TaPHT1.1* and *TaPHT1.2*). This organic acid exudation by the wheat roots was many times higher than without Si application, and the P uptake was doubled. There is insufficient data regarding the effect of exogenous Si on organic acid production in plants. In a recent study, it was found that Si can alter organic acid production in plants by increasing carbon fluxes into TCA cycle and the activity of TCA cycle enzymes (Das et al., 2019). However, further work is needed to elucidate how Si modulates organic acid metabolism in plants under P deficit conditions.

Much remains to be investigated on how Si interferes with soil P mobilization. Some mechanisms by which Si improves soil P availability and plant P uptake are as follows: (i) competitive exchange and sorptive interaction of P and Si (Smyth and Sanchez, 1980; Koski-Vähälä et al., 2001; Owino-Gerroh and Gascho, 2005; Konhauser et al., 2007; Planavsky et al., 2010). P binding to soil minerals was observed to be the lowest with silicate minerals (Rajan, 1975; Brady and Weil, 1999); (ii) increasing the soil pH to enhance soil P availability in acidic soils (Owino-Gerroh and Gascho, 2005); (iii) indirectly improving P utilization by plants by decreasing the uptake and availability of metals (Hingston, 1972; Sigg and Stumm, 1981; Schwertmann and Fechter, 1982; Ma and Takahashi, 1990). P availability is controlled by levels of other metals such as Fe and Mn under P deficiency. A large proportion of soil Pi is strongly bound/adsorbed to aluminum, iron and manganese hydroxides in the soil (Beauchemin et al., 2003). Si decreases the iron and manganese availability in soil by affecting the element binding to the soil particles (Schaller et al., 2019) and reducing the pool of hydroxides (Treder and Cieslinski, 2005; Meharg and Meharg, 2015) and can therefore indirectly increase P availability (Ma, 2004; Greger et al., 2018). Si may increase P availability for plants even in high P conditions by mobilizing P from such mineral surfaces (e.g., aluminum, iron and manganese hydroxides) (Cross and Schlesinger, 1995; Yang and Post, 2011); (iv) modifying the C:N:P stoichiometry and improving the nutrient use efficiency (Neu et al., 2017); (v) increasing the root organic anion efflux to mobilize the rhizospheric Pi (Kostic et al., 2017; Etesami and Jeong, 2018). Si significantly increased the exudation rates of citrate and malate to directly stimulate inorganic P acquisition by the roots (Kostic et al., 2017). Organic anions such as acetic, aconitic, citric, malic, fumaric, lactic, oxalic, and succinic acids compete with Pi to form complexes with aluminum, calcium, and iron and may hydrolyze organic P (Grierson, 1992; Gerke et al., 2000; Hinsinger, 2001; Kihara et al., 2003; Aziz et al., 2011; Etesami and Jeong, 2018). Organic acids like malic and citric acids were observed to reduce the pH and result in a substantially increased P mobilization from calcium compounds (Dinkelaker and Marschner, 1992) and effectively enhanced P uptake from sparingly soluble rock phosphates (Aziz et al., 2011); (*vi*) enhancing the gene expressions related to Pi uptake under P deficiency, which is key to improving the Pi absorption in different plant species (Leggewie et al., 1997; Karthikeyan et al., 2002; Tittarelli et al., 2007; Miao et al., 2009; Kostic et al., 2017). The P use efficiency of plants under P deficiency could be improved with manipulation of gene expressions related to Pi uptake (Aziz et al., 2014). A number of genes are involved with plant adaptation to P deficiency, associated with regulating the acquisition, internal remobilization of P, and changing the metabolism as well as signaling transduction (Fang et al., 2009). Si has been observed to modulate the expression of stress-related genes and alter plant metabolism in response to various plant stresses (Pavlovic et al., 2013; Ye et al., 2013; Kim et al., 2014; Kostic et al., 2017); and (vii) mobilizing or desorbing of organic carbon from soil particles or mineral binding sites (e.g., goethite) (Tipping, 1981; Reithmaier et al., 2017). Si has a strong bonding affinity to minerals in the soil comparable with carbon and P, and may mobilize the two elements and make them more available for microbial decomposition (Schaller et al., 2019). The released carbon can supply the microorganisms, including PSB, with energy for their growth in soils. The carbon dioxide produced by microbial respiration results in the production of carbonic acid, leading to increased P availability. Microbial respiration was observed to lower the soil pH by producing carbonic acid, and thus led to dissolution of apatite as Pi (Guidry and Mackenzie, 2003). How the Si availability in soils interacts with P availability in soils is generally poorly understood and requires further research.

## Synergistic Effects of AMF, PSB, and Si on P Availability

### Synergistic Effects of AMF and PSB on P Availability

**FIGURE 2:**
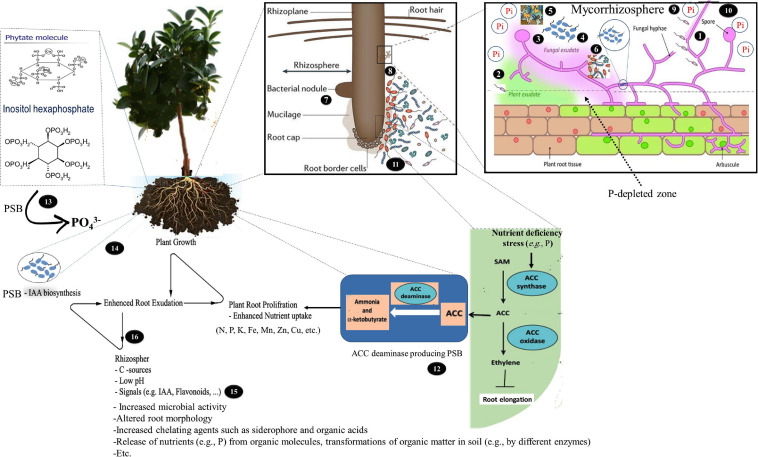
A schematic representation of how the interaction between arbuscular mycorrhizal fungi (AMF) and phosphate-solubilizing bacterium (PSB) affect the host plant’s utilization of the organic and inorganic phosphorus (P). **(1)** PSB use the hyphae to access greater soil volumes further away, which can benefit the fungi as phosphorus solubilizers may grow into insoluble-phosphorus-containing patches away from the route along the AMF hyphae; **(2)** PSB can colonize the rhizosphere (potentially rich with resources from plant exudates) by using the AMF hyphae to allow growth in other directions towards the plant; **(3)** AMF can supply energy-rich carbohydrates (C) via the extraradical hyphae for PSB; **(4)** the fungal exudates can change the rhizospheric pH, influence the chemical composition of the root exudates, and increase microbial activity; **(5)** AM hyphae and mycelial exudates can improve soil structure by binding to soil particles and through glomalin production, which may glue soil particles together and subsequently affect soil moisture retention; **(6)** AMF colonization can influence the bacterial community compositions in the mycorrhizosphere; **(7)** AMF can increase the ability of legumes to fix N_2_ and reduce the amount of inorganic nitrogen that leaches, increasing plant growth and subsequently enhancing root exudates; **(8)** PSB can stimulate the AMF colonization; **(9)** AMF, unlike root hairs, can extend much farther away from the root surfaces. In other words, the extraradical hyphae extend beyond the phosphorus depletion zone, to absorb the bioavailable phosphates that are otherwise inaccessible for plants. In addition, AMF-solubilized phosphates and bacteria are more efficiently taken up by plants through a mycorrhizal-mediated channel between the plant roots and the surrounding soil; **(10)** AMF can take up and transfer inorganic phosphorus to the roots by their effective mycorrhizal mycelium, reaching microhabitats where orthophosphates are made available with phosphorus-mobilizing microorganisms and preventing quick immobilization of orthophosphates by microbial biomass; **(11)** PSB can improve the nitrogen and phosphorus availability for AMF and plants through organic matter decomposition; **(12)** PSB (ACC deaminase positive), by synthesizing ACC deaminase, can lower the stress ethylene levels, which is involved in stimulating the growth of AMF; **(13)** PSB can increase the AMF hyphal growth by hydrolyzing organic phosphorus with secretion of enzyme phosphatases and phytases and providing mineral phosphorus for the fungi; **(14)** PSB (IAA positive) can increase the IAA levels, resulting in more lateral roots that form the preferred penetration sites for AM hyphae. In addition, AMF can use the IAA the related compounds as a part of their colonization strategy to interact with plants, which leads to stimulated plant growth and modified basal plant defense mechanisms; **(15)** PSB can promote the induce flavonoid release from plants to promote mycorrhiza formations, thus facilitating root colonization; and **(16)** PSB (IAA positive) can loosen plant cell walls to promote root exudation to provide additional nutrients that support the microbial growth of microbes.

In mycorrhizal association, the plant and fungi interact both in the soil around the root (rhizosphere) and in soil around the fungal hyphae (mycorrhizosphere) (Johansson et al., 2004). The fungi interact with other microorganisms in the mycorrhizosphere whose synergistic effects increase plant growth and also populations of both (Artursson et al., 2006; Agnolucci et al., 2015). The presence of different bacterial taxa that colonized the surface of AM extraradical hyphae and spores that form biofilm–like structures on them has been reported in natural ecosystems (Scheublin et al., 2010; Lecomte et al., 2011; Cruz and Ishii, 2012; Agnolucci et al., 2015; Iffis et al., 2016). There may exist cooperation between AMF and the associated bacteria, such as PSB (Zhang et al., 2016). PSB may provide the hyphae with Pi and rely on the carbon released by AMF. Earlier research demonstrated that AMF and PSB may enhance P acqusition of the AM host plant through their interactactions (Kim et al., 1997; Toro et al., 1997; Sharma et al., 2013; Calvo et al., 2014; Figure 2). However, the mechanisms by which this nutritional improvement is brought about remain unclear (Artursson et al., 2006). In the following sections, what is currently known of how AMF and PSB influence each other and, consequently, increase P availability, are discussed separately.

### Effects of PSB on AMF and AMF-Mediated P Availability

#### Effect of PSB on solubilizing insoluble phosphates

The phosphate-solubilizing activities of AMF are still controversial although AM plants have generally been shown to increase the uptake of insoluble Pi (Yao et al., 2001; Klugh-Stewart and Cumming, 2009; Campos et al., 2018). In many studies, mycorrhizal inoculants were observed to alter the composition and/or amount of total low molecular weight organic acids (LMWOAAs) exuded by AM plants (Klugh and Cumming, 2007; Klugh-Stewart and Cumming, 2009). However, direct evidence for solubilization of P by AM fungi has not been obtained to date. Despite the fact that AM fungi might not exude LMWOAAs by themselves, they can, however, improve P solubilization and/or mineralization indirectly by stimulating the surrounding soil microbes via the exudation of labile C, thus increasing local nutrient availability in the hyphosphere and in soil patches beyond the root hairs (Hodge et al., 2009; Cheng et al., 2012; Jansa et al., 2013).

PSB solubilize phosphates and release Pi ions from the sparingly soluble organic/inorganic P compounds found in nature into a form that AMF can acquire and deliver to the plant (Toro et al., 1997, 1998; Ordoñez et al., 2016). ^32^P-Labeling studies have shown that mycorrhiza increase the absorptive root surface areas to facilitate P uptake, but do not help in P solubilization (Gaur, 2003). In another ^32^P-labeling study, seven bacterial strains isolated from AMF spores facilitated P uptake by promoting the development of AM extraradical mycelium (Battini et al., 2017).

Arbuscular mycorrhizal fungi cannot extract P on their own from indigenous less-available forms of P sources, such as rock phosphates, and can only absorb Pi ions from the soil solution (Antunes et al., 2007). However, with the help of certain bacteria (Villegas and Fortin, 2001) AMF can acquire P from rock phosphates and translocate it to the host plant. AMF were able to acquire P from sources that were otherwise inaccessible with the help of PSB (Toro et al., 1997). These interactions can also indirectly benefit plants; *Medicago sativa* shoot P concentrations were observed to be improved (Zhang et al., 2016). The interaction between the two microorganism groups may lead to synergistic effects. It has been found that the AMF–PSB interactions only benefit plants when additional P was also supplied (Zhang et al., 2016). Zhang et al. (2014a) showed that P concentrations available in the soil regulate P mobilization and immobilization to determine the bacterial P contribution to plants. In general, when the available P level is low in soils, AMF and PSB compete for the P, and this competition is not stimulated by the fungi. With additional P supply, PSB improved the AMF hyphal growth, and the PSB activities were stimulated by the fungi (Zhang et al., 2016).

#### Effect of PSB on mineralizing organic P

During evolution with plants, AMF have lost the genes encoding proteins involved in saprotrophic function (Tisserant et al., 2013), which means that they cannot directly breakdown soil organic matter (Leigh et al., 2011; Zhang et al., 2014b). PSB can increase the AMF hyphal growth by hydrolyzing organic P with secretion of the phosphatases and phytases and providing mineral P to the fungi (Dobbelaere et al., 2003; Wang et al., 2016; Zhang et al., 2016). It has been reported that AMF cannot secrete phosphatases (Tisserant et al., 2013) and directly decompose organic nutrients (Smith and Read, 2008; Tisserant et al., 2013). AM fungi possess many genes encoding acid phosphatases in their genomes, with at least seven genes expressed in *Rhizophagus clarus* (Sato et al., 2015). However, secretion of phosphatases is mostly associated with the cell wall (Olsson et al., 2002) and their presence in the rhizosphere has been demonstrated only in limited cases (Tarafdar and Marschner, 1994; Koide and Kabir, 2000). The magnitude of these processes is questioned as it is difficult to isolate the effects of plants, fungi and others microorganisms present in the experiments under non-sterile conditions (Joner and Jakobsen, 1995; Joner et al., 2000). In an *in vitro* monoxenic culture, Sato et al. (2015) provided evidence that the acid phosphatase activity originated from *R. clarus*. Nevertheless, the interaction of AM association with the phosphatase activity and the subsequent P acquisition by efficient genotypes is still unclear (Campos et al., 2018).

**FIGURE 3:**
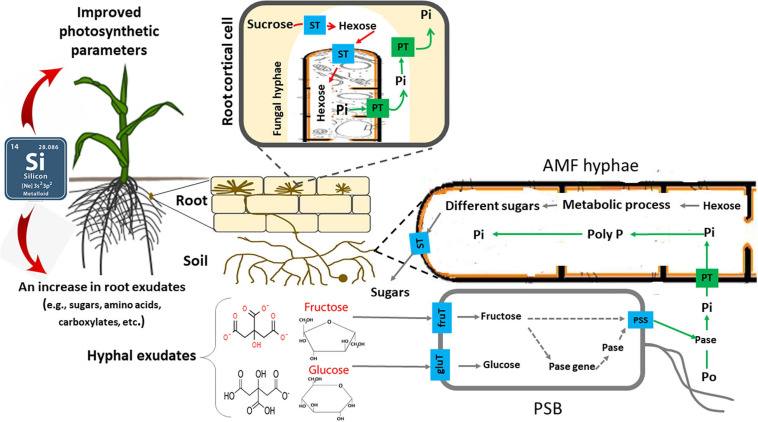
Schematic representation of role of silicon (Si), arbuscular mycorrhizal fungi (AMF), and phosphate-solubilizing bacteria (PSB) in mineralizing organic phosphorus (Po). Si increases photosynthetic products in root and rhizosphere by improving photosynthetic parameters of plant. The fructose exuded by AMF stimulates the expression of phosphatase genes in PSB as well as the rate of phosphatase release into the growth medium by regulating their protein secretory system. The phosphatase activity is subsequently increased, promoting the mineralization of Po (i.e., phytate) into Pi, stimulating simultaneously the processes involved in P uptake by the AMF. ST, sugar transporter; fruT, fructose transporter; glut, glucose transporter; PT, phosphate transporter; PSS, protein secretory system; Pase, phosphatase; Pi, inorganic P; and Po, organic P. For more details, see Zhang et al. (2018a).

Because AMF are unable to release phosphatases outside the hyphae, AMF’s organic P utilization appears to depend on the recruitment of other soil microbes (Tisserant et al., 2013; Zhang et al., 2016). The microbiome associated with the hyphae may play a key role in AMF’s utilization of organic P. AMF may shift the microbiome composition to influence organic P mineralization (Zhang et al., 2016, 2018b). Importantly, AMF hyphae seem to specifically recruit bacteria that produce alkaline phosphatase which can mineralize organic P; these species are not found when AMF is excluded (Zhang et al., 2018b). Despite the fact that a major AMF function is to increase P availability to plants (Smith and Read, 2008), AMF cannot release phosphatases into the soil (Tisserant et al., 2013; Zhang et al., 2016). In a study under controlled, sterile conditions, the AMF *Rhizophagus irregularis* DAOM 197198 released carbon-rich compounds to stimulate PSB functions, but did not directly influence the phosphatase activities (Zhang et al., 2016). Thus, AMF cannot directly utilize organic P, which limits its contribution to plant uptake of P. PSB accounts for up to 40% of all culturable bacteria (Jorquera et al., 2008) and can make up for this defect in AMF. This suggests that AMF and PSB need to interact to help plants uptake P (Zhang et al., 2018b). Recent results demonstrate that the AMF hyphal surfaces are colonized by PSB and the hyphal exudates are utilized as a carbon source (Wang et al., 2016). In other words, AMF can attract PSB and help them multiply to improve the organic P utilization by releasing hyphal exudates and providing a carbon source (Zhang et al., 2014b, 2016; Wang et al., 2016). PSB can then colonize the AMF hyphal surfaces (Wang et al., 2016). This enhances the activities of the phosphatases released by the PSB, and stimulates the organic P mineralization (Zhang et al., 2016). The extraradical AMF hyphae can then access Pi released from organic P sources (Tarafdar and Marschner, 1994; Feng et al., 2003). In addition, AMF hyphae-associated PSB in the soil play an important role in phytate P mineralization and that the AMF primes the mineralization and turnover of the organic P (organic P utilization affected by the AMF-bacteria interaction) (Zhang et al., 2014b). For example, in a recent study Zhang et al. (2018a) observed that fructose exuded by an arbuscular mycorrhizal fungus, *Rhizophagus irregularis*, stimulated the expression of phosphatase genes in a phosphate solubilizing bacterium, *Rahnella aquatilis*, as well as the rate of phosphatase release into the growth medium by regulating its protein secretory system. The phosphatase activity was also subsequently increased, promoting the mineralization of organic P (i.e., phytate) into Pi, stimulating simultaneously the processes involved in P uptake by *Rh. irregularis*. In general, PSB can increase P availability for AMF, especially from organic P sources, which may increase the expression of Pi transporter genes in the AMF hyphae (Zhang et al., 2016; Figure 3).

#### Effect of PSB on arbuscular mycorrhizal symbiosis

Bacteria are known to influence AMF fitness (Frey-Klett et al., 2007; Scheublin et al., 2010; Nuccio et al., 2013) and ecological functions (Hodge et al., 2001; Feng et al., 2003; Cheng et al., 2012; Zhang et al., 2014b). PSB can lead to increased plant growth parameters by stimulating the native AMF’s establishment, growth rate, multiplication, and spore germination (Barea et al., 2002; Bianciotto and Bonfante, 2002; Artursson et al., 2006; Frey-Klett et al., 2007; Berta et al., 2014). PSB can promote AMF extraradical hyphal growth and allow PSB to explore a greater volume of the mycorrhizosphere and AMF hyphae to gain access to new solubilized P sources (Ordoñez et al., 2016). Increased mycelial growth of *Glomus mosseae* spores, for example, was reported to be caused by an unidentified PGP rhizobacterium (Azcón, 1987). These bacteria also helped mycorrhiza by promoting root colonization by indigenous and introduced AMF (Toro et al., 1997). Bacteria can promote hyphal growth and facilitate root penetration by AMF via producing compounds that increase cell penetrability and result in increased root exudation rates (Hildebrandt et al., 2002; Jeffries et al., 2003; Jäderlund et al., 2008). Following hyphal growth, the rates of root colonization and AMF development also increase (Barea et al., 2005; Richardson et al., 2009). Bacterial IAA is known to be able to loosen plant cell walls and therefore promote root exudation which supplies additional nutrition that can support microbial growth (Chaintreuil et al., 2000; Sevilla et al., 2001; James et al., 2002; Chi et al., 2005). One of components of root exudates is enzymes such as amylase, DNase, phosphatase, polygalacturonase, protease, RNase, sucrase, urease, and xylanase that can play a role in organic P mineralization, decomposition of other organic compounds, and release of mineral elements (Ahemad and Kibret, 2014; Canarini et al., 2019), and, therefore, provide mineral P and other elements for the AMF. It is well established that bacterial IAA increases the ability of plants to convert nutrients from non–available forms to available forms by increasing the root system, root discharge and microbial flora (Etesami et al., 2015b). Bacteria IAA–mediated release of root exudates can enhance P mobility for plants and AMF by releasing protons (H^+^) or by forming amino/organic acid mineral complexes (by chelation of cations accompanying P e.g., Fe^3+^, Al^3+^, and Ca^2+^), and indirectly (as a source of nutrients for microorganisms) by stimulating the microbial activities in the rhizosphere (functioning, growth, propagation, survival) (Etesami et al., 2015b). The increased soil saprobiotic microbial populations mediated by root exudates can, in turn, improve N availability for AMF through organic matter decomposition (Leigh et al., 2011; Herman et al., 2012; Nuccio et al., 2013). Other microorganisms attracted to root exudates stimulate hyphal growth, mycorrhizal colonization, and spore production, thereby increasing AMF fitness (Frey-Klett et al., 2007). Flavonoids are the main signaling compounds that are isolated from plant root exudates, and it’s been suggested that they play a distinct role in the AM development. Different flavonoids affect the growth and differentiation of the hyphae as well as root colonization in a structure–specific manner. Flavonoids also influence presymbiotic growth differently according to the genus and species. Furthermore, it has also been proposed that some of the so-called mycorrhiza helper bacteria that promote mycorrhiza formation induce flavonoid release from plants, and facilitate root colonization by mycorrhizal plants (Schrey et al., 2014). A number of studies have demonstrated that the IAA-secretion induced stimulation of root hair growth and lateral root elongation supplies more active sites and access for symbiotic AMF and PSB associations (Aarab et al., 2015; Etesami et al., 2015b). Therefore, it seems that PSB (IAA positive) stimulate root hair elongation to improve root weight and architecture, and therefore potentially improve mycorrhizal formation. Previous studies have shown that PGPB modify hormonal signaling in plants to influence root architecture, stimulate the growth of the shoots and roots, and increase essential nutrient uptake (Appanna, 2007; Bhattacharyya and Jha, 2012). AMF-induced plant growth is in part attributed to modified plant hormone level (Bi et al., 2019; Wang et al., 2021).

The relationship between the AMF and host roots is complex and requires a continuous exchange of signals which leads to a developmental coexistence (Gianinazzi-Pearson, 1996; Hause and Fester, 2005). Phytohormones are the signals that regulate various plant growth processes and can therefore manage colonization and AM symbiosis formation (Barker and Tagu, 2000; Ludwig-Müller and Güther, 2007; Foo et al., 2013; Gutjahr, 2014). For example, bacterial IAA may increase the number of lateral roots for fungi to colonize in early growth stages to facilitate host colonization (Kaldorf and Ludwig-Müller, 2000). Increased IAA levels and IAA–induced gene expressions have been suggested to contribute to phenotypic changes during mycorrhizal colonization (Ludwig-Müller and Güther, 2007). Fungi may use IAA and the related compounds as a colonization strategy to interact with plants, stimulating plant growth and modifying basal plant defense mechanisms (Prusty et al., 2004; Contreras-Cornejo et al., 2009). Generally, the increased levels of IAA result in more lateral roots that form the preferred penetration sites for the AM hyphae.

Abscisic acid (ABA) is a sesquiterpenoid hormone, derived from carotenoids, which functions at multiple levels to regulate AM symbiosis (Ludwig-Müller, 2010). ABA deficiency also results in the induction of ethylene production, which adversely affects mycorrhizal interaction with plants (Herrera-Medina et al., 2007; Martín-Rodríguez et al., 2011). The synthesis of ACC deaminase produced by PSB lowers the stress ethylene levels associated with stimulating AMF growth (Gamalero et al., 2008; Etesami et al., 2015b).

In addition, gibberellins (GAs), key regulators of plant growth and development, play a role during arbuscular mycorrhizal (AM) formation (Foo et al., 2013; Martín-Rodríguez et al., 2015, 2016; Foo et al., 2016; Pons et al., 2020). GAs inhibit arbuscular mycorrhizal symbioses (McGuiness et al., 2019) by altering GA response changes in the expression of genes associated with mycorrhizal colonization (Martín-Rodríguez et al., 2015), inhibiting AM hyphal entry into the host root, and suppressing the expression of reduced arbuscular mycorrhization1 (RAM1) and RAM2 homologs that function in hyphal entry and arbuscule formation (Takeda et al., 2015). The balance between ABA and GAs is also essential for AM formation in plant roots (Martín-Rodríguez et al., 2016; McGuiness et al., 2019) as the imbalance in the ABA/GAs ratio can reduce arbuscule abundance in mycorrhizal roots (Martín-Rodríguez et al., 2015). In addition, GA signaling also positively interacts with symbiotic responses and promotes AM colonization of the host root. For example, in one study (Takeda et al., 2015), low GA conditions suppressed arbuscular mycorrhiza-induced subtilisin-like serine protease1 (SbtM1) expression, which is required for AM fungal colonization and reduced hyphal branching in the host root. In this study, the reduced hyphal branching and SbtM1 expression due to the inhibition of GA biosynthesis were recovered by GA treatment, supporting the theory that insufficient GA signaling causes inhibitory effects on arbuscular mycorrhiza development. Accordingly, it seems that PSB positive for ABA and GA-producing traits can regulate the level of production of these hormones in the plant and lead to improved arbuscular mycorrhizal symbioses. The ability to produce GAs in some bacteria has been reported (Hamayun et al., 2010; Kang et al., 2012; Tatsukami and Ueda, 2016; Etesami and Glick, 2020). However, it is not yet clear if mycorrhizal fungi produce GA. Therefore, the presence of such bacteria is necessary to improve mycorrhizal symbioses.

#### Effects of AMF on PSB and PSB-Mediated P Availability

Mycorrhizae affect both the composition and number of the rhizospheric and hyphospheric bacterial communities (Offre et al., 2007; Agnolucci et al., 2015; Taktek et al., 2015), as well as bacterial communities of the surface of the AMF hyphae or mycelium closely attached to the soil (Zhang et al., 2014b; Turrini et al., 2018). AMF result in the establishment of an extensive soil hyphal network, creating a dedicated niche for bacteria (Bianciotto and Bonfante, 2002; Agnolucci et al., 2015). In the cytoplasm of some AMF isolates belonging to the Gigasporaceae family endophytic bacteria are found, which is a case where bacteria coexist with fungi (Turrini et al., 2018). The bacterial colonization of the AMF hyphal and spore surfaces has been confirmed with molecular and microscopic analyses and illustrates the existence of a close relationship between the two microorganism groups (Toljander et al., 2006; Bharadwaj et al., 2008; Scheublin et al., 2010; Agnolucci et al., 2015). Similar to roots, AMF hyphae are rapid channels for photosynthates and release carbon-rich compounds into the soil (Toljander et al., 2007; Bharadwaj et al., 2012) and can stimulate microbial growth and function (Drigo et al., 2010; Leigh et al., 2011; Blagodatskaya and Kuzyakov, 2013; Kaiser et al., 2015; Zhang et al., 2016). The root exudates are a major nutrient source for the rhizospheric PSB, and its chemical composition may be influenced by the AMF (Artursson et al., 2006). Furthermore, the extensive extraradical AMF hyphae and the exudates create conditions that can influence bacterial activities and growth (Toljander et al., 2007; Bharadwaj et al., 2012; Gahan and Schmalenberger, 2015) including PSB (Taktek et al., 2015; Ordoñez et al., 2016; Wang et al., 2016; Turrini et al., 2018). The changes in the soil bacterial community composition induced by AMF are described, both under *in vivo* and controlled conditions (Marschner et al., 2001; Toljander et al., 2006, 2007).

Arbuscular mycorrhizal fungi enhance the chlorophyll content, PSII photochemical efficiency, and net photosynthetic rate of plants (Wu and Xia, 2006; Zhu et al., 2014; Augé et al., 2016; Shi-Chu et al., 2019) and also transfer plant photosynthates underground, which can stimulate PSB activity and growth (Zhang et al., 2016) as most PSB are heterotrophic and depend on nutrient substrates that can be easily decomposed. AMF hyphae are also rapid channels for the recently produced photosynthates, which can attract PSB and promote their growth (Kaiser et al., 2015). In addition, it has been found that the availability of easily decomposable organic compounds limits microbial P solubilization in soil extracts from phosphate minerals (Brucker and Spohn, 2019; Brucker et al., 2020). Saprotrophic phosphate solubilizing microorganisms in mineral soils generally lack sufficient carbon (Demoling et al., 2007; Heuck et al., 2015) because most organic carbon in soils is protected from sorptive or recalcitrant microbial decomposition or is simply spatially inaccessible (De Nobili et al., 2001; Dungait et al., 2012). Accordingly, increased microbial P solubilization rates are reported when carbon sources become available (Hameeda et al., 2006; Patel et al., 2008).

Arbuscular mycorrhizal fungi generate a vast extraradical hyphae in the soil that microorganisms can inhabit (Gahan and Schmalenberger, 2015). PSB can grow alongside AMF hyphae in and out of the root, in sterile conditions as well as with an indigenous microbial community (Ordoñez et al., 2016), demonstrating the close relationship of AMF and PSB (Scheublin et al., 2010; Agnolucci et al., 2015). This may help PSB to use the hyphae to access areas further away in the soil to acquire insoluble P. By developing an external mycelium, AMF, upon root colonization, connect the root with the surrounding soil microhabitats and can contribute to nutrient capture and supply (Toro et al., 1997). The PSB can also use the AMF hyphae to allow growth in the direction toward the plant, colonize the rhizosphere, and use more plant exudates (Ordoñez et al., 2016).

Phosphate–solubilizing bacteria attachment to the extraradical AMF hyphae can ensure that P solubilization would occur in locations where it is the most beneficial for fungi to access the additional soluble P. The phosphate solubilized by AMF and PSB is effectively absorbed by plants through a channel formed by the mycorrhiza between plant roots and the surrounding soil (Artursson et al., 2006).

Since the mobilized orthophosphates can quickly be immobilized by microbial biomass, AMF can absorb and transfer the nutrients to the roots through their effective mycorrhizal mycelium, and reach microhabitats where orthophosphates are made available by P-mobilizing microorganisms (Richardson et al., 2009). AMF cannot directly decompose organic nutrients, as they have no known saprotrophic capabilities (Smith and Read, 2008; Tisserant et al., 2013). AMF can also increase the soil saprobiotic microbe activities, including those of PSB. These bacteria can decompose organic matter and in turn also improve the N and P availability for AMF and plants (Leigh et al., 2011; Herman et al., 2012; Nuccio et al., 2013). Previously, Linderman (1992) reported that AMF enhance the activity of nitrogen–fixing bacteria (NFB) and PSB and thus promote plant growth. PSB can also release diffrent enzymes to decompose the organic matter, and can provide mineral nutrients for the AMF hyphae (Hodge and Fitter, 2010; Hodge, 2014; Zhang et al., 2014b). Therefore, in exchange for using the carbon released by the AMF, these microbes can provide additional benefits to the fungi. AMF and PSB may obtain their required nutrients from their partners and enhance their own fitness through cooperation. By increasing the root surface areas for nutrient acquisition, or through more specific mechanisms, AMF can also help plants resist abiotic and biotic stresses (Artursson et al., 2006; Miransari, 2010; Sikes, 2010; Mohammad and Mittra, 2013). PSB solubilize phosphates into forms that are usable by the AMF, and AMF can absorb the P and transport it to the plant using a range of mechanisms. AMF may also help spread PSB to neighboring rhizospheres. Therefore, AMF and PSB interact synergistically.

### AMF Increase Si Uptake by Plants

The benefits of Si nutrition, although significant, are limited due to its restricted uptake by plant (Anda et al., 2016). However, AMF such as *Glomus etunicatum*, *G. versiform, G. coronatum*, *Rhizophagus clarus* (=*Glomus clarum*), *Rhizophagus irregularis* (=*Glomus intraradices*), and *Funneliformis mosseae* (= *Glomus mosseae*) were observed to increase Si uptake in the roots and shoots of mycorrhizal plants (i.e., *Saccharum* spp., *Glycine max* L*., Zea mays* L., *Cajanus cajan* L., *Cicer arietinum* L., strawberry, and banana) compared to non–mycorrhizal plants (Yost and Fox, 1982; Kothari et al., 1990; Clark and Zeto, 1996; Clark and Zeto, 2000; Nogueira et al., 2002; Hammer et al., 2011; Garg and Singla, 2015; Anda et al., 2016; Garg and Bhandari, 2016a,b; Frew et al., 2017; Garg and Singh, 2018; Hajiboland et al., 2018; Gbongue et al., 2019; Moradtalab et al., 2019). AMF play a substantial role in Si uptake, translocation from the external solution to the intraradical mycelium, and transfer from the fungal cells to the root cells. The mechanisms remain unclear but it is not excluded that active transport is involved via transporters located within the extraradical hyphae at the soil-fungus interface for the uptake of Si and at the plant–fungal interface (i.e., arbuscule) for its transfer across the peri–arbuscular interface in the plant cells (Yost and Fox, 1982; Hammer et al., 2011; Anda et al., 2016; Garg and Bhandari, 2016b). These studies highlight the importance of AMF inoculation as tools to effectively enhance Si uptake by plants. Therefore, it would be of great interest to investigate how AM symbiosis enhances the host plant uptake of Si and how AM symbiosis and Si uptake help to improve P nutrition and plant growth.

### Si Increases Mycorrhizal Effectiveness in Plants

Mycorrhizal effectiveness (or responsiveness of plants to mycorrhizae) is defined as the difference in the growth of plants with and without mycorrhizae (Janos, 2007). Mycorrhizal effectiveness is influenced by different factors like fungal species, plant species and genotype, and soil conditions (Tawaraya, 2003). Compared to the studies widely performed on the effects of P availability as a soil chemical factor on the mycorrhizal effectiveness, research on how Si affects mycorrhizal effectiveness are lacking. However, in two recent studies (Hajiboland et al., 2018; Moradtalab et al., 2019), mycorrhizal effectiveness was increased with Si treatments in strawberry plants inoculated with AMF *Rhizophagus clarus*, *Rhizophagus intraradices*, and *Glomus versiform* compared to AMF plants not treated with Si. Some known mechanisms by which Si benefits the AMF effectiveness include: (i) enhancing the uptake and transfer of nutrients for plants and stimulating the root growth in AMF plants, which can lead to promoted AMF colonization (Hajiboland et al., 2018); (ii) increasing the photosynthetic rate such that the fungal partner is able to receive a greater carbon supply, for example, by increasing the leaf chlorophyll levels, photosynthetic enzyme activities, and stomatal conductance (Guntzer et al., 2012; Hajiboland, 2012) (Figure 3) and improving the leaf stability so that leaves are oriented more horizontally (Botta et al., 2014). Since 4–20% of the fixed carbon from photosynthesis is transferred to the AMF, the mycorrhizal association relies on the organic carbon supplied from their photosynthetic partners (Smith and Read, 2008). Furthermore, the photosynthetic rate (organic carbon supply) is positively correlated with the hyphal absorption capacity and arbuscule formation (Smith and Read, 2008; Anda et al., 2016; Moradtalab et al., 2019); (iii) modifying the phenolic metabolic pathways in AMF host plants and/or reducing polymerization and lignin synthesis (Rodrigues et al., 2004; Mandal et al., 2010; Hajiboland et al., 2018), which can affect how AMF interact with the host plant. Studies have investigated how Si affects the metabolism of phenolic compounds in plants (Dragišić Maksimović et al., 2007; Hajiboland et al., 2017). Phenolic compounds, such as flavonoids, are known to potentially help facilitate AMF to interact with their host plants (Vierheilig, 2004; Mandal et al., 2010), improve fungal growth parameters such as branching, hyphal growth, spore germination (Steinkellner et al., 2007) and secondary spore formation, and contribute to the fungal invasion and root arbuscule formation (Hassan and Mathesius, 2012); and (*iv*) increasing the pool of soluble sugars in the roots, which is crucial for the entry and further establishment in the roots, of AMF (Moradtalab et al., 2019). Future research should investigate the metabolic and molecular mechanisms that are associated with the synergistic relationship of Si and AMF.

### PSB Increase the Availability and Uptake of Si for Plants

Phosphate–solubilizing bacteria generally have the ability to weather silicates, likely because basic metabolic activities like organic acid production and respiration can cause the weathering of minerals (Brucker et al., 2020). PSB mainly solubilize insoluble Pi by acidifying the microenvironment (Etesami, 2020). In addition to increasing P availability for plants, there are some reports that PSB are also able to increase Si availability and uptake. Lee et al. (2019) observed that the PSB strain *Enterobacter ludwigii* GAK2, isolated from paddy soils, was able to significantly increase P and Si levels in rice plant tissues grown on insoluble Pi [Ca_3_(PO_4_)_2_] and insoluble silicate (Mg_2_O_8_Si_3_) based soils. This bacterial strain also increased rice plant growth indices (chlrophyll content, fresh biomass, root and shoot lengths). In another study, the PSB strains *Bacillus simplex* UT1 and *Pseudomonas* sp. FA1 significantly increased the shoot Si levels in sorghum (*Sorghum bicolor* L.) (Rezakhani et al., 2019a) and wheat (*Triticum aestivum* L.) (Rezakhani et al., 2019b). Given the role of Si in increasing soil P availability, one question that arises here is whether the Si-mediated increase in soil P availability has an inhibitory effect on bacterial solubilization of P from insoluble Pi sources. It is noteworthy that microbial P solubilization is not influenced by the soil P availability. For example, in a study (Brucker et al., 2020), adding P (100 mg of ground apatite) to soil extracts from soils with various P fractions (bioavailable P between 0.6 to 38 mg kg^–1^ and total P between 0.42 to 1.23 g kg^–1^) and degree of weathering, which had been incubated 28 days, did not substantially reduce P solubilization rates, which indicates that the P availability does not affect the microbial soil P solubilization. It is probable that microbial P solubilization is not driven by the microbial P demand but rather is a side effect of microbial metabolism. It was also observed that P fertilization over several years did not influence PSB abundance in the grassland soils of different continents (Widdig et al., 2019). Generally, PSB can benefit plants by accelerating the weathering of silicates and increasing the rhizospheric concentration of available Si.

### Si Increases the Population of PSB

The potential effect of Si on the soil microbial community has attracted only a limited amount of attention. However, there are some reports showing that Si can significantly influences some soil microbial community components (e.g., it increased beneficial bacterial population and reduced soil fungi/bacteria ratio) (Wainwright et al., 2003; Hordiienko et al., 2010; Karunakaran et al., 2013; Wang et al., 2013; Lin et al., 2020). It is reported that bacteria use Si-based autotrophy as a source of energy to support CO_2_ fixation (Das et al., 1992). It is also proposed that the first bacteria may have evolved on earth because of Si (Wainwright et al., 2003). A number of bacteria and fungi are able to grow on nutrient-free silica gel and distilled water (Wainwright et al., 1991). According to a study (Wainwright et al., 2003), silicic acid increases the number of both aerobic and facultative anaerobic bacteria in ultra-pure water incubated under strict oligotrophic conditions. In addition, organisms use silica through silicification, a process by which silica is utilized and deposited by bacteria (Perry, 2003) and also Si-based compounds stimulate the population of oligotrophic bacteria in soil (Ai-Falih, 2003). In a previous study (Karunakaran et al., 2013), it was shown that the microbial population increased with an increase in concentration of nanosilica. In addition, silica content in biomass also increased with an increase in the concentration of nanosilica. It is known that nanosilica is not toxic to the soil bacterial community (Karunakaran et al., 2013). The reason behind the interaction between nanosilica and bacteria may reflect a hydration property of the nanosilica surface, which could facilitate the attraction of silica to the microbial surface (Gordienko and Kurdish, 2007).

There are a few studies that have focused on the effect of Si application on the activity and population of PSB. In one study, the efficiency of nanosilica (0.5 g kg^–1^ of soil) was evaluated in terms of its effects on beneficial microbial population such as PSB in the rhizosphere soil of maize (Rangaraj et al., 2014). When compared with the control (2.0 × 10^4^ CFU g^–1^), the silica-treated rhizosphere soils revealed an increase in the PSB population (4.4 × 10^4^ CFU g^–1^). This shows that the addition of silica may act as a substrate for P uptake systems in soil as well as in plants. An increase in the population of PSB of nanosilica-amended soil indicates enhanced soil fertility and enhanced available nutrients to the plants. The increased population of PSB in nanosilica-treated rhizosphere soil may be due to the availability of more P to plants, as the Si competes for P in a plant system. Because both P and Si influence the P content and the population of PSB, the uptake of either source increases the populations of PSB. Hence, silica may act as a substrate for PSB, which results in an increase in the population of PSB and availability of P. The changes in soil inorganic nutrients with respect to silica fertilization may also be due to the production of organic compounds by increased microbial activity and desorption of inorganic nutrients from soil mineral compounds (Karunakaran et al., 2013). It is also reported that the bacteria use silica from soil and hence, there is a decrease in the silica content in the soil (Wainwright et al., 2003). Thus, nanosilica can be included for fertilizer formulations to make the soil more fertile and to improve soil phosphate-solubilizing bacterial community for improving plant P nutrition.

## SSB Increase Availability of P and Si and Their Uptake by Plants

Plants are not able to absorb Si until monosilicic acid (H_4_SiO_4_) is released into the soil solution through weathering or dissolution (Kang et al., 2017). Monosilicic acid generally originates from the weathering of soil minerals that contain Si, desorption from the soil matrix, irrigation water, and Si fertilizers (Klotzbücher et al., 2015). Si fertilizers, unlike conventional fertilizers, have a limited availability and are often not affordable for many farmers (Meena et al., 2014). Therefore, they are rarely used in many countries, especially in developing countries. Silicate fertilizers are usually composed of (*i*) industrial byproducts or slags rich in Si, whose application may lead to metal contamination of soils, (*ii*) bentonite, diatomaceous earth, feldspars, and micas, which are biologically/minerally derived Si fertilizers with low Si bioavailability and high application rates and (*iii*) highly soluble, but very expensive, potassium silicates (Datnoff et al., 2001). Si-rich crop residues, construction/demolition wastes that contain aluminum, calcium, and potassium silicates, mineral/metal mining wastes, and silicate rocks may be recycled to affordable silicate fertilizers. The solubility of the primary and secondary minerals in soils is the main factor that influences the soil Si concentration (Sommer et al., 2006). The primary and secondary minerals can be subjected to physico-chemical and biochemical interventions that accelerate the solubility for soil applications (Bin et al., 2008), but biochemical action via microbial activities is considered most important for this process (Vasanthi et al., 2018). Many studies have observed that microbes isolated from the surface of silicate minerals weather different silicates (Sheng et al., 2008; Lapanje et al., 2012; Wang et al., 2015).

Plants and their associated microflora are known to also influence silicate weathering (the dissolution and mobilization of silicate minerals in soil) by altering the physical soil properties, modifying the soil pH, and producing chelating ligands (Cornelis et al., 2011). It has been reported that among microorganisms, plant associated bacteria accelerate the dissolution of silicates and release Si to the plant–soil system (Savant et al., 1996; Hutchens et al., 2003; Sheng and He, 2006; Uroz et al., 2009; Chandrakala et al., 2019) through bio-weathering processes (Klotzbücher et al., 2015). With an increase in knowledge of how Si benefits plants, rhizospheric soils have been explored in search of new bacteria that solubilize silicates (Kang et al., 2017; Vasanthi et al., 2018). SSB have been gathering increasing interest, as rhizospheric silicate solubilization leads to increased potassium and Si uptake, which reduces the need for potash fertilizers.

The ability to solubilize silicates (to depolymerize crystalline silicate) has been reported in various Gram-positive and Gram-negative bacteria (*Burkholderia eburnea* CS4-2, *Bacillus* sp*., Bacillus flexus, Bacillus globisporus, B. mucilaginosus, B. megaterium* and *Pseudomonas fluorescens, Burkholderia susongensis* sp., *Rhizobium* sp*., Rhizobium yantingense*, *Rhizobium tropici*, and *Pseudomonas stutzeri*) (Malinovskaya et al., 1990; Lin et al., 2002; Liu et al., 2006; Vasanthi et al., 2013, 2018; Chen et al., 2015; Gu et al., 2015; Wang et al., 2015; Umamaheswari et al., 2016; Kang et al., 2017; Chandrakala et al., 2019).

Silicate-solubilizing bacteria can potentially release soluble silica from biogenic materials such as diatomaceous earth, rice husks, rice straw, and siliceous earth, as well as from insoluble, inorganic (Al, Ca, K, and Mg) silicates and silicate minerals such as feldspar and biotite (Wang et al., 2015; Chandrakala et al., 2019). These bacteria have been isolated from different habitats, such as rice plant rhizospheres (Kang et al., 2017; Chandrakala et al., 2019), from rice field soil samples (Vasanthi et al., 2013), weathered feldspar surfaces (Sheng and He, 2006), weathered rock surfaces (Gu et al., 2015), weathered rock (purple siltstone) surfaces (Chen et al., 2015), pond sediments, river water, soils, and talc minerals (Umamaheswari et al., 2016), potassium mine tailings (Huang et al., 2013), quercus petreae oak mycorrhizal roots surroundings (Calvaruso et al., 2010), and weathered rocks (Wang et al., 2015).

Some mechanisms which SSB could utilize to release soluble silica from insoluble silicates include: (i) production of organic acids including citric, tartaric, acetic, gluconic, hexadecanoic, malic, oxalic, phthalic, oleic, heptadecanoic, and hydroxypropionic acids (Vassilev et al., 2006; Vasanthi et al., 2018), which have metal complexing properties that may bind with aluminum and iron silicates and render silicates soluble, also provide protons (H^+^) for protonation for silicate hydrolysis (Duff and Webley, 1959; Avakyan et al., 1986; Drever and Stillings, 1997); (ii) inorganic acid production (i.e., oxidation of sulfur, reduction of sulfides to sulfuric acid, oxidation of ammonia to nitrates, and conversion of nitrates to nitric acid, which can act on silicates); (iii) synthesis and discharge of carbonic anhydrase that catalyzes the interconversion between carbon dioxide produced by soil microbes and water, and the dissociated ions of carbonic acid (Brucker et al., 2020), which promotes the microbial conversion of silicate minerals as observed in orthoclase degradation to kaolinite (Waksman and Starkey, 1924). In addition, CO_2_ sequestration in basaltic acquifers and the associated carbonate mineralization might maintain an environment suitable for silicate mineral dissolution (Pokrovsky et al., 2011); (iv) production of siderophores, which bind and transport iron(III) which can play a part in silicate solubilization by scavenging iron from silicate minerals as observed in hornblende degradation (Kalinowski et al., 2000); (v) the reduction of sulfates and production of H_2_S, which reacts with cations like Ca and Fe of silicate minerals forming sulfides and thus rendering silicate solubilization (Ehrlich et al., 2015); (vi) absorption and binding of the inorganic silicate ions on bacterial surfaces, due to having ionizable carboxylates and phosphates of lipopolysaccharides in Gram-negative bacteria and peptidoglycan, teichoic acids and teichoic acids in Gram-positive bacteria and their high reactivity to the ions, rendering dissolution (Urrutia and Beveridge, 1994); (vii) extracellular polysaccharide production (Xiao et al., 2016; Kang et al., 2017), which is implicated in weathering of rocks and breakdown of silicates due to their wetting and drying properties and acting as a sorbent of metal ions (binding silicates and affecting the equilibrium between the fluid and mineral phases, rendering them soluble) during this vital activity. The biofilm formation also solubilizes silicates in their microenvironment (Malinovskaya et al., 1990); and (viii) alkali production (Kutuzova, 1969). It is known that SSB can solubilize silicates by shifting the pH of the environment toward alkalinity by decomposing the organic matter and fixing nitrogen, to subsequently form ammonia and amines (Vasanthi et al., 2018); and acidolysis, the most commonly found mechanism of silicate mineral weathering (Jongmans et al., 1997; Chandrakala et al., 2019). Future research should focus on the yet unknown mineral weathering mechanisms of these bacteria (Kang et al., 2017).

These bacteria, isolated from both plant roots and soil minerals, could also increase the plant Si uptake and therefore Si levels in plants (Peera et al., 2016; Kang et al., 2017; Vasanthi et al., 2018; Chandrakala et al., 2019). In one study (Kang et al., 2017), it was found that inoculation of japonica rice plants with the SSB strain *Burkholderia eburnea* CS4-2 increased the Si content in the plants grown on the plant growth substrate including insoluble silicates. In addition, the plant growth attributes (chlorophyll levels, root and shoot lengths, root and shoot fresh weights, etc.) were also improved compared to those of the control and of plants grown on insoluble silica. CS4-2, when applied together with insoluble silica, significantly promoted the growth of rice plants (Kang et al., 2017). Chandrakala et al. (2019) found that the SSB strain *Rhizobium* sp. IIRR-1 isolated from the rhizospheric soil around rice plants could colonize and grow on all insoluble silicates, which resulted in increased silica release into the culture media (12.45–60.15% over that of the control). This strain also successfully colonized the roots of rice seedlings and improved their vigor by 29.18% compared to that of the control.

In addition to providing plants with Si, SSB can also solubilize P and other nutrients like Ca, Fe, K, Mg, and Zn, bound to the silicate minerals from insoluble sources and provide plants with P (Lin et al., 2002; Liu et al., 2006; Vasanthi et al., 2018; Chandrakala et al., 2019; Lee et al., 2019). The mechanisms for P solubilization are also responsible for the biogenic silicate weathering; namely, the release of extracellular polysaccharides, organic acids, protons, and siderophores (Vassilev et al., 2006; Gorbushina and Broughton, 2009; Uroz et al., 2009; Etesami, 2020). Silicate weathering provides access to minerals that contain P, such as apatite, which are calcium phosphates, and therefore is also related to P solubilization (Gorbushina and Broughton, 2009; Uroz et al., 2009). For example, studies have reported that SSB produce organic acids such as acetic, formic, and gluconic acids during the solubilization of insoluble tri-calcium silicates and other insoluble nutrient sources (Park et al., 2009). The aforementioned studies show that SSB utilization may improve the solubilization of insoluble P and Si, which could ultimately increase the plant P and Si uptake and to substantially improve the plant growth and health. Compared to the studies performed on PSB and other PGPB, very few studies have been conducted to isolate SSB with plant growth-promoting activities from plant-associated soils (Kang et al., 2017; Vasanthi et al., 2018), likely because SSB only accounts for a low proportion of the total bacteria that exist in soils and silicate minerals (Vasanthi et al., 2018).

**FIGURE 4:**
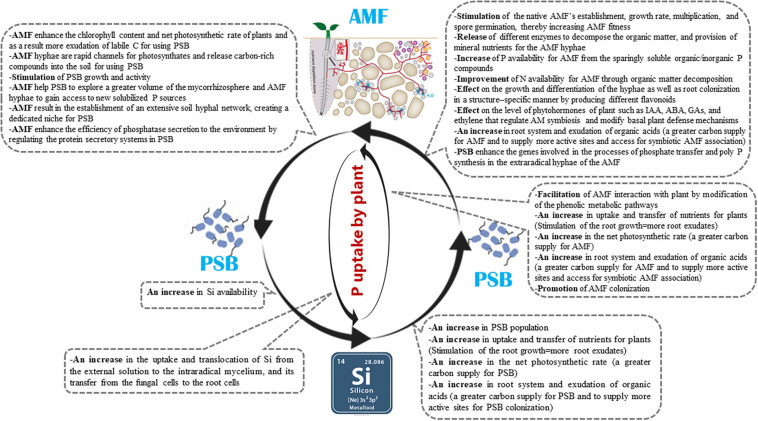
Synergistic effects of among arbuscular mycorrhizal fungi (AMF), phosphate-solubilizing bacterium (PSB), and silicon (Si) on P Availability and its uptake by plant.

The outcome of this review paper may widen research scope for use of Si/nanosilica (or SSB) in combination with AMF and PSB in improving P use efficiency in sustainable agriculture (Figure 4).

## Future Prospects

Arbuscular mycorrhizal fungi provide their host plants with P and other nutrients in exchange for photosynthates, by effectively increasing the volume of the soil solution that host plants can acquire minerals from via the hyphae that develop from the roots. AMF prevent the available P from re-precipitating before plants have access, and their capacity to transport P to plants can account for up to 80% of a plant’s total P uptake. However, much of the soil P exists in an insoluble form, and some AMF can only exploit soluble P sources. PSB can solubilize these insoluble P forms and potentially make them available for absorption by AMF hyphae and plants. PSB also increase the AMF hyphal expression Pi transporter genes. In addition to increasing P availability in soils, Si also enhances the expression of plant genes associated with inorganic P absorption under P-deficient conditions. Previous studies, indicate that AMF and PSB, Si and AMF, and Si and PSB synergistically act to more effectively increase the plant uptake of P, improving the growth of different plants more than when each was applied on its own. Accordingly, the use of Si along with these two microbial groups may increase P availability in the rhizosphere.

Several suggestions and avenues of research would move us closer to adopting this strategy for developing environment-friendly P-biofertilizer to be used as supplements and/or alternatives to chemical P fertilizers:

(i) It is known that AMF and PSB cooperate, in addition to having synergistic effects (Zhang et al., 2016). According to Zhang et al. (2016) AMF and free–living PSB cooperated for mutual benefit by supplying the required carbon or P for each microorganism, though these interactions were dependent on the environmental P availability. This indicates that when using co–inoculation of AMF and PSB, the amount of available P in the soil should be considered;
(ii) AMF hyphae, by secreting certain metabolites such as carbohydrates (Hooker et al., 2007; Toljander et al., 2007) (e.g., sugars such as galactose, fructose, glucose, and trehalose) (Zhang et al., 2018a, b), carboxylates (e.g., aconitate, citrate, and succinate) (Tawaraya et al., 2006; Zhang et al., 2018b), and amino acids (Bharadwaj et al., 2012), only benefit specific microorganisms and inhibit others (Nuccio et al., 2013; Bender et al., 2014). The exact mechanisms of these interactions remains to be investigated, although previous research has made suggestions: physical interactions such as the capacity to attach to the hyphae of the AMF (Scheublin et al., 2010), niche competition for nutrients (Veresoglou et al., 2011), and AMF hyphal exudation directly and indirectly manipulating the community (Toljander et al., 2007). In addition, it is important to note that the stimulated microbes positively affect AMF fitness in general (Scheublin et al., 2010; Nuccio et al., 2013);
(iii) It has also been found that relative to other PSB strains, the different bacteria may positively or negatively influence the AMF hyphal growth. Future research should investigate which combination and plant-growth-promoting characteristics are the most affected by fungal secretions, as the knowledge is yet unclear. Furthermore, it should be investigated which soil conditions (available P content, pH, organic matter composition, etc.) lead to the best results in plants when the co–inoculation of AMF and PSB with Si is provided;
(iv) Further research is necessary to identify the different mechanisms with which spore- and AMF hyphae-associated PSB affect plant growth with and without AMF in non-sterile conditions in the field. Evidence to date is still inconsistent regarding significant organic P mineralization by AMF. Continuous monitoring of the characteristics of the different AMF mycelial exudates, and how they interact with the biotic and abiotic environments *in situ* will also help further the understanding AMF’s ecological roles (Toljander, 2006);
(v) The relationship between certain soil bacteria and mycorrhizal fungi provides new insights into the design of mixed inoculation, while identifying fungal strains that contain plant growth-promoting endosymbiotic bacteria and mycorrhiza helper bacteria (bacterial communities living strictly associated with AMF spores extraradical mycelium and mycorrhizal roots, in the mycorrhizosphere) evidenced by ACC deaminase activities, IAA production, siderophore production, Pi solubilization, and N_2_ fixation ability enables new strategies for AMF use (Turrini et al., 2018). A better understanding of such relationships between certain bacteria and fungi should lead to substantial ecological benefits and contribute to sustainable agriculture;
(vi) To make optimal use of soil microorganisms to maximize the benefits for plant growth and development, future research should investigate how the soil bacteria and fungi interact. Calcareous soils with a high pH and low P availability could benefit greatly from making use of such microorganism interactions;
(vii) Since the coexistence of AMF and PSB in the rhizosphere spans millions of years, numerous interactions should have evolved between the two microorganism groups. The exact mechanisms between AMF and PSB should be identified. It is not clear whether the phosphate-solubilizing ability of any bacteria allows them to attach to the extraradical AMF hyphae (Scheublin et al., 2010);
(viii) Most research has been done using culturable bacteria. Since most bacteria are unculturable, more research is needed dealing with unculturable bacteria in the mycorrhizosphere, which will lead to an improved knowledge of the microbial community and the associated mycorrhizospheric interactions (Toljander, 2006);
(ix) Further research to evaluate the application and the efficacy of AMF, PSB, and SSB (or Si fertilizers) as co–inoculants or as independent inoculants under various environmental stresses on crops fertilized with different low-solubility P sources in real world conditions is necessary, where the survival of AMF, PSB, and SSB, as well as how the mechanisms with which they promote plant growth is affected by competition with the endemic microorganisms, environmental stresses, and soil conditions;
(x) Further research is necessary to validate the AMF and PSB performance in conjunction with SSB or suitable insoluble silicate sources, as silica itself is considered as agronomically beneficial and its mobilization is always accompanied by the release of other macronutrients and macronutrients that are bound to silicate minerals, under various field conditions and different ecosystems; and
(xi) Because AMF are unable to release phosphatases outside the hyphae, organic P utilization by AMF seems to depend on the presence of other soil microbes. Since different bacterial genera possess different organic P mineralization abilities (Rodrìguez and Fraga, 1999) and multiple plant-growth-promoting characteristics, bacteria are expected to promote plant growth more effectively in comparison to microorganisms that only possess a single plant growth-promoting trait (Shahi et al., 2011; Etesami and Maheshwari, 2018); manipulation of the bacterial community associated with the AMF hyphae (i.e., introduction of superior PSB and SSB into the hyphae) may influence the organic P mineralization and Si uptake processes in plants.

## Conclusion

**FIGURE 5:**
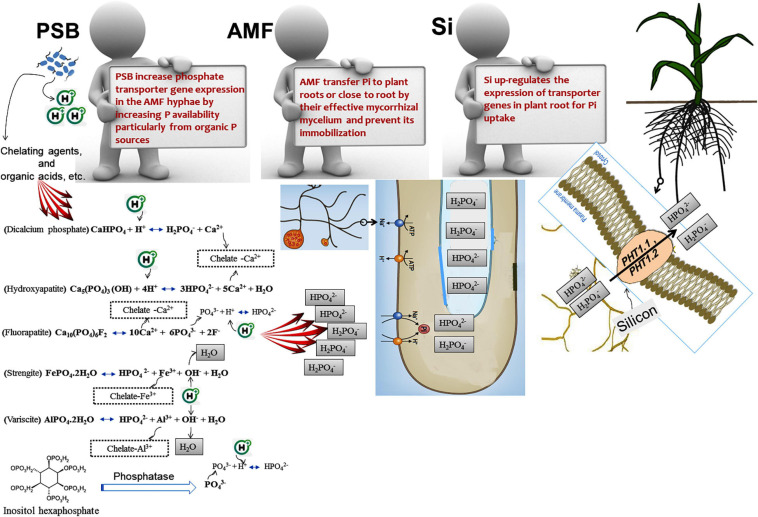
Synergetic role of among arbuscular mycorrhizal fungi (AMF), phosphate-solubilizing bacterium (PSB), and silicon (Si) in improving phosphorus (P) uptake by plant.

P is a vital element in crop nutrition. Adverse environmental effects of chemical-based P fertilizers have compelled us to find a sustainable approach for efficient P availability in agriculture to meet the ever-increasing global demand of food. According to the review paper, the use of AMF, PSB, and the addition of Si can be an effective and economical way to improve the availability and efficacy of P. Based on what is known about them, the combination of AMF, PSB, and Si (or SSB) may be utilized as a strategy for improving plant growth in P-deficient soils and minimizing chemical fertilization to exercise sustainable agriculture (Figure 5). The combination can help plants effectively utilize the low-solubility P sources by solubilizing them into utilizable forms that are later absorbed by plants. This may assist in solving problems encountered with the crop production economy and food shortages, which also make the co-inoculation with Si or SSB a promising technique for use in commercial inoculant formulations.

## Author Contributions

HE wrote the manuscript. BJ checked and edited the sections related to silicon in this manuscript. BG revised and approved the final version to be published. All the authors contributed to the article and approved the submitted version.

## Conflict of Interest

The authors declare that the research was conducted in the absence of any commercial or financial relationships that could be construed as a potential conflict of interest.

## Footnotes

1 http://www.amf-phylogeny.com

## References

[B1] Aarab S. Ollero F. J. Megías M. Laglaoui A. Bakkali M. Arakrak A. (2015). Isolation and screening of bacteria from rhizospheric soils of rice fields in Northwestern Morocco for different plant growth promotion (PGP) activities: an in vitro study. *Int. J. Curr. Microbiol. Appl. Sci.* 4 260–269.

[B2] Adesemoye A. O. Torbert H. A. Kloepper J. W. (2009). Plant growth-promoting rhizobacteria allow reduced application rates of chemical fertilizers. *Microb. Ecol.* 58 921–929. 10.1007/s00248-009-9531-y 19466478

[B3] Agnolucci M. Battini F. Cristani C. Giovannetti M. (2015). Diverse bacterial communities are recruited on spores of different arbuscular mycorrhizal fungal isolates. *Biol. Fertility Soils* 51 379–389. 10.1007/s00374-014-0989-5

[B4] Ahemad M. (2012). Implications of bacterial resistance against heavy metals in bioremediation: a review. *J. Institute Integrative Omics Appl. Biotechnol (IIOAB)* 3 39–46.

[B5] Ahemad M. Khan M. (2010). Phosphate solubilizing *Enterobacter* asburiae strain PS2. *Afr. J. Microbiol. Res.* 5 849–857.

[B6] Ahemad M. Kibret M. (2014). Mechanisms and applications of plant growth promoting rhizobacteria: current perspective. *J. King Saud University-science* 26 1–20. 10.1016/j.jksus.2013.05.001

[B7] Ahmed E. Holmström S. J. M. (2014). Siderophores in environmental research: roles and applications. *Microbial Biotechnol.* 7 196–208. 10.1111/1751-7915.12117 24576157 PMC3992016

[B8] Ai-Falih A. M. (2003). Effect of silicon compounds on oligotrophic soil bacteria. *Saudi. J. Biol. Sci.* 10 131–137.

[B9] Alori E. T. Glick B. R. Babalola O. O. (2017). Microbial phosphorus solubilization and its potential for use in sustainable agriculture. *Front. Microbiol.* 8:971. 10.3389/fmicb.2017.00971 28626450 PMC5454063

[B10] Amir H. Pineau R. (2003). Release of Ni and Co by microbial activity in New Caledonian ultramafic soils. *Can. J. Microbiol.* 49 288–293. 10.1139/w03-039 12897838

[B11] Anda C. C. O. Opfergelt S. Declerck S. (2016). Silicon acquisition by bananas (cV Grande Naine) is increased in presence of the arbuscular mycorrhizal fungus Rhizophagus irregularis MUCL 41833. *Plant Soil* 409 77–85. 10.1007/s11104-016-2954-6

[B12] Antunes P. M. Schneider K. Hillis D. Klironomos J. N. (2007). Can the arbuscular mycorrhizal fungus Glomus intraradices actively mobilize P from rock phosphates? Pedobiologia 51 281–286. 10.1016/j.pedobi.2007.04.007

[B13] Appanna V. (2007). Efficacy of phosphate solubilizing bacteria isolated from vertisols on growth and yield parameters of sorghum. *Res. J. Microbiol.* 2 550–559. 10.3923/jm.2007.550.559

[B14] Artursson V. Finlay R. D. Jansson J. K. (2006). Interactions between arbuscular mycorrhizal fungi and bacteria and their potential for stimulating plant growth. *Environ. Microbiol.* 8 1–10. 10.1111/j.1462-2920.2005.00942.x 16343316

[B15] Augé R. M. Toler H. D. Saxton A. M. (2016). Mycorrhizal stimulation of leaf gas exchange in relation to root colonization, shoot size, leaf phosphorus and nitrogen: a quantitative analysis of the literature using meta-regression. *Front. Plant Sci.* 7:1084. 10.3389/fpls.2016.01084 27524989 PMC4965464

[B16] Avakyan Z. A. Pivovarova T. A. Karavaiko G. I. (1986). Properties of a new species, bacillus-mucilaginosus. *Microbiology* 55 369–374.

[B17] Azcón R. (1987). Germination and hyphal growth of Glomus mosseae in vitro: effects of rhizosphere bacteria and cell-free culture media. *Soil Biol. Biochem.* 19 417–419. 10.1016/0038-0717(87)90032-0

[B18] Aziz T. Ahmed I. Farooq M. Maqsood M. A. Sabir M. (2011). Variation in phosphorus efficiency among Brassica cultivars I: internal utilization and phosphorus remobilization. *J. Plant Nutrition* 34 2006–2017. 10.1080/01904167.2011.610487

[B19] Aziz T. Sabir M. Farooq M. Maqsood M. A. Ahmad H. R. Warraich E. A. (2014). “Phosphorus deficiency in plants: responses, adaptive mechanisms, and signaling,” in *Plant signaling: Understanding the Molecular Crosstalk.* Hakeem K. R. Rehman R. U. Tahir I. eds (Springer:Berlin), 133–148. 10.1007/978-81-322-1542-4_7

[B20] Badri D. V. Vivanco J. M. (2009). Regulation and function of root exudates. *Plant Cell Environ.* 32 666–681. 10.1111/j.1365-3040.2009.01926.x 19143988

[B21] Balestrini R. Gómez-Ariza J. Lanfranco L. Bonfante P. (2007). Laser microdissection reveals that transcripts for five plant and one fungal phosphate transporter genes are contemporaneously present in arbusculated cells. *Mol. Plant Microbe Interact.* 20 1055–1062. 10.1094/mpmi-20-9-1055 17849708

[B22] Barea J. -M. Pozo M. J. Azcon R. Azcon-Aguilar C. (2005). Microbial co-operation in the rhizosphere. *J. Exp. Bot.* 56 1761–1778. 10.1093/jxb/eri197 15911555

[B23] Barea J. M. Toro M. Orozco M. O. Campos E. Azcón R. (2002). The application of isotopic (32P and 15N) dilution techniques to evaluate the interactive effect of phosphate-solubilizing rhizobacteria, mycorrhizal fungi and Rhizobium to improve the agronomic efficiency of rock phosphate for legume crops. *Nutrient Cycling Agroecosystems* 63 35–42.

[B24] Barker S. J. Tagu D. (2000). The roles of auxins and cytokinins in mycorrhizal symbioses. *J. Plant Growth Regulat.* 19 144–154. 10.1007/s003440000021 11038224

[B25] Bashan Y. Holguin G. De-Bashan L. E. (2004). Azospirillum-plant relationships: physiological, molecular, agricultural, and environmental advances (1997-2003). *Can. J. Microbiol.* 50 521–577. 10.1139/w04-035 15467782

[B26] Bashan Y. Kamnev A. A. De-Bashan L. E. (2013). Tricalcium phosphate is inappropriate as a universal selection factor for isolating and testing phosphate-solubilizing bacteria that enhance plant growth: a proposal for an alternative procedure. *Biol. Fertility Soils* 49 465–479. 10.1007/s00374-012-0737-7

[B27] Battini F. Grønlund M. Agnolucci M. Giovannetti M. Jakobsen I. (2017). Facilitation of phosphorus uptake in maize plants by mycorrhizosphere bacteria. *Sci. Rep.* 7:4686. 10.1038/s41598-017-04959-0 PMC5498536 28680077

[B28] Beauchemin S. Hesterberg D. Chou J. Beauchemin M. Simard R. R. Sayers D. E. (2003). Speciation of phosphorus in phosphorus-enriched agricultural soils using X-ray absorption near-edge structure spectroscopy and chemical fractionation. *J. Environ. Qual.* 32 1809–1819. 10.2134/jeq2003.1809 14535324

[B29] Begum N. Qin C. Ahanger M. A. Raza S. Khan M. I. Ashraf M. (2019). Role of arbuscular mycorrhizal fungi in plant growth regulation: implications in abiotic stress tolerance. *Front. Plant Sci.* 10:1068. 10.3389/fpls.2019.01068 31608075 PMC6761482

[B30] Bender S. F. Plantenga F. Neftel A. Jocher M. Oberholzer H. -R. Köhl L. (2014). Symbiotic relationships between soil fungi and plants reduce N2O emissions from soil. *ISME J.* 8 1336–1345. 10.1038/ismej.2013.224 24351937 PMC4030222

[B31] Benedetto A. Magurno F. Bonfante P. Lanfranco L. (2005). Expression profiles of a phosphate transporter gene (GmosPT) from the endomycorrhizal fungus *Glomus mosseae*. *Mycorrhiza* 15 620–627. 10.1007/s00572-005-0006-9 16133249

[B32] Berta G. Copetta A. Gamalero E. Bona E. Cesaro P. Scarafoni A. (2014). Maize development and grain quality are differentially affected by mycorrhizal fungi and a growth-promoting pseudomonad in the field. *Mycorrhiza* 24 161–170. 10.1007/s00572-013-0523-x 23995918

[B33] Bharadwaj D. P. Alström S. Lundquist P. -O. (2012). Interactions among Glomus irregulare, arbuscular mycorrhizal spore-associated bacteria, and plant pathogens under in vitro conditions. *Mycorrhiza* 22 437–447. 10.1007/s00572-011-0418-7 22081167

[B34] Bharadwaj D. P. Lundquist P. -O. Alström S. (2008). Arbuscular mycorrhizal fungal spore-associated bacteria affect mycorrhizal colonization, plant growth and potato pathogens. *Soil Biol. Biochem.* 40 2494–2501. 10.1016/j.soilbio.2008.06.012

[B35] Bhattacharyya P. N. Jha D. K. (2012). Plant growth-promoting rhizobacteria (PGPR): emergence in agriculture. *World J. Microbiol. Biotechnol.* 28 1327–1350. 10.1007/s11274-011-0979-9 22805914

[B36] Bi Y. Xiao L. Sun J. (2019). An arbuscular mycorrhizal fungus ameliorates plant growth and hormones after moderate root damage due to simulated coal mining subsidence: a microcosm study. *Environ. Sci. Poll. Res.* 26 11053–11061. 10.1007/s11356-019-04559-7 30790167

[B37] Bianciotto V. Bonfante P. (2002). Arbuscular mycorrhizal fungi: a specialised niche for rhizospheric and endocellular bacteria. *Antonie Van Leeuwenhoek* 81 365–371. 12448735 10.1023/a:1020544919072

[B38] Bin L. Ye C. Lijun Z. H. U. Ruidong Y. (2008). Effect of microbial weathering on carbonate rocks. *Earth Sci. Front.* 15 90–99. 10.1016/S1872-5791(09)60009-9

[B39] Blagodatskaya E. Kuzyakov Y. (2013). Active microorganisms in soil: critical review of estimation criteria and approaches. *Soil Biol. Biochem.* 67 192–211. 10.1016/j.soilbio.2013.08.024

[B40] Bona E. Cantamessa S. Massa N. Manassero P. Marsano F. Copetta A. (2016). Arbuscular mycorrhizal fungi and plant growth-promoting pseudomonads improve yield, quality and nutritional value of tomato: a field study. *Mycorrhiza* 27 1–11. 10.1007/s00572-016-0727-y 27539491

[B41] Borch K. Bouma T. Lynch J. Brown K. (1999). Ethylene: a regulator of root architectural responses to soil phosphorus availability. *Plant Cell Environ.* 22 425–431. 10.1046/j.1365-3040.1999.00405.x

[B42] Botta A. Rodrigues F. A. Sierras N. Marin C. Cerda J. M. Brossa R. (2014). “Evaluation of Armurox^®^ (complex of peptides with soluble silicon) on mechanical and biotic stresses in gramineae,” in *Proceedings of the 6th International Conference on Silicon in Agriculture*, Stockholm, Sweden, 26–30.

[B43] Bouhraoua D. Aarab S. Laglaoui A. Bakkali M. Arakrak A. (2015). Phosphate solubilizing bacteria efficiency on mycorrhization and growth of peanut in the northwest of Morocco. *Am. J. Microbiol. Res.* 3 176–180.

[B44] Brady N. C. Weil R. R. (1999). *The Nature and Properties of Soil* 12th edn.New York, NY:Mac. Pub. Com. 625–640.

[B45] Browne P. Rice O. Miller S. H. Burke J. Dowling D. N. Morrissey J. P. (2009). Superior inorganic phosphate solubilization is linked to phylogeny within the *Pseudomonas fluorescens* complex. *Appl. Soil Ecol.* 43 131–138. 10.1016/j.apsoil.2009.06.010

[B46] Brucker E. Spohn M. (2019). Formation of soil phosphorus fractions along a climate and vegetation gradient in the Coastal Cordillera of Chile. *Catena* 180 203–211. 10.1016/j.catena.2019.04.022

[B47] Brucker E. Kernchen S. Spohn M. (2020). Release of phosphorus and silicon from minerals by soil microorganisms depends on the availability of organic carbon. *Soil Biol. Biochem.* 143:107737. 10.1016/j.soilbio.2020.107737

[B48] Brundrett M. C. Tedersoo L. (2018). Evolutionary history of mycorrhizal symbioses and global host plant diversity. *New Phytol.* 220 1108–1115. 10.1111/nph.14976 29355963

[B49] Bruns T. D. Corradi N. Redecker D. Taylor J. W. Öpik M. (2018). Glomeromycotina: what is a species and why should we care? *New Phytol.* 220 963–967. 10.1111/nph.14913 29165821

[B50] Bücking H. Liepold E. Ambilwade P. (2012). The role of the mycorrhizal symbiosis in nutrient uptake of plants and the regulatory mechanisms underlying these transport processes. *Plant Sci.* 4 108–132.

[B51] Bünemann E. K. Oberson A. Frossard E. (2010). *Phosphorus in Action: Biological Processes in Soil Phosphorus Cycling.* Berlin:Springer Science & Business Media.

[B52] Caballero-Mellado J. Onofre-Lemus J. Estrada-De Los Santos P. Martínez-Aguilar L. (2007). The tomato rhizosphere, an environment rich in nitrogen-fixing *Burkholderia species* with capabilities of interest for agriculture and bioremediation. *Appl. Environ. Microbiol.* 73 5308–5319. 10.1128/aem.00324-07 17601817 PMC1950987

[B53] Calvaruso C. Turpault M. -P. Leclerc E. Ranger J. Garbaye J. Uroz S. (2010). Influence of forest trees on the distribution of mineral weathering-associated bacterial communities of the *Scleroderma citrinum* mycorrhizosphere. *Appl. Environ. Microbiol.* 76 4780–4787. 10.1128/aem.03040-09 20511429 PMC2901721

[B54] Calvo P. Nelson L. Kloepper J. W. (2014). Agricultural uses of plant biostimulants. *Plant Soil* 383 3–41. 10.1007/s11104-014-2131-8

[B55] Campos P. Borie F. Cornejo P. López-Ráez J. A. López-García Á. Seguel A. (2018). Phosphorus acquisition efficiency related to root traits: is mycorrhizal symbiosis a key factor to wheat and barley cropping? *Front. Plant Sci.* 9:752. 10.3389/fpls.2018.00752 29922321 PMC5996197

[B56] Canarini A. Kaiser C. Merchant A. Richter A. Wanek W. (2019). Root exudation of primary metabolites: mechanisms and their roles in plant responses to environmental stimuli. *Front. Plant Sci.* 10:157. 10.3389/fpls.2019.00157 30881364 PMC6407669

[B57] Carey J. C. Fulweiler R. W. (2012). The terrestrial silica pump. *PLoS One* 7:e52932. 10.1371/journal.pone.0052932 23300825 PMC3534122

[B58] Carlisle E. M. Mckeague J. A. Siever R. Van Soest P. J. (1977). “Silicon,” in *Geochemistry and the Environment.* Elsevier:Washington, DC, 54–115.

[B59] Castagno L. Estrella M. Sannazzaro A. Grassano A. Ruiz O. (2011). Phosphate-solubilization mechanism and in vitro plant growth promotion activity mediated by *Pantoea eucalypti* isolated from *Lotus tenuis* rhizosphere in the Salado River Basin (Argentina). *J. Appl. Microbiol.* 110 1151–1165. 10.1111/j.1365-2672.2011.04968.x 21299771

[B60] Chaintreuil C. Giraud E. Prin Y. Lorquin J. Ba A. Gillis M. (2000). Photosynthetic bradyrhizobia are natural endophytes of the African wild rice *Oryza breviligulata*. *Appl. Environ. Microbiol.* 66 5437–5447. 10.1128/aem.66.12.5437-5447.2000 11097925 PMC92479

[B61] Chandrakala C. Voleti S. R. Bandeppa S. Kumar N. S. Latha P. C. (2019). Silicate solubilization and plant growth promoting potential of *Rhizobium sp*. isolated from rice rhizosphere. *Silicon* 11 2895–2906. 10.1007/s12633-019-0079-2

[B62] Chen W. Sheng X. -F. He L. -Y. Huang Z. (2015). Rhizobium yantingense sp. nov., a mineral-weathering bacterium. *Int. J. Systematic Evol. Microbiol.* 65 412–417. 10.1099/ijs.0.064428-0 25376852

[B63] Cheng L. Booker F. L. Tu C. Burkey K. O. Zhou L. Shew H. D. (2012). Arbuscular mycorrhizal fungi increase organic carbon decomposition under elevated CO2. *Science* 337 1084–1087. 10.1126/science.1224304 22936776

[B64] Chi F. Shen S. H. Cheng H. P. Jing Y. X. Yanni Y. G. Dazzo F. B. (2005). Ascending migration of endophytic rhizobia, from roots to leaves, inside rice plants and assessment of benefits to rice growth physiology. *Appl. Environ. Microbiol.* 71 7271–7278. 10.1128/aem.71.11.7271-7278.2005 16269768 PMC1287620

[B65] Chiba Y. Mitani N. Yamaji N. Ma J. F. (2009). HvLsi1 is a silicon influx transporter in barley. *Plant J.* 57 810–818. 10.1111/j.1365-313x.2008.03728.x 18980663

[B66] Clark R. Á. Zeto S. K. (2000). Mineral acquisition by arbuscular mycorrhizal plants. *J. Plant Nutrition* 23 867–902. 10.1080/01904160009382068

[B67] Clark R. B. Zeto S. K. (1996). Mineral acquisition by mycorrhizal maize grown on acid and alkaline soil. *Soil Biol. Biochem.* 28 1495–1503. 10.1016/s0038-0717(96)00163-0

[B68] Contreras-Cornejo H. A. Macías-Rodríguez L. Cortés-Penagos C. López-Bucio J. (2009). Trichoderma virens, a plant beneficial fungus, enhances biomass production and promotes lateral root growth through an auxin-dependent mechanism in *Arabidopsis*. *Plant Physiol.* 149 1579–1592. 10.1104/pp.108.130369 19176721 PMC2649400

[B69] Cordell D. Drangert J. -O. White S. (2009). The story of phosphorus: global food security and food for thought. *Global Environ. Change* 19 292–305. 10.1016/j.gloenvcha.2008.10.009

[B70] Cornelis J.-T. Delvaux B. Georg R. Lucas Y. Ranger J. Opfergelt S. (2011). Tracing the origin of dissolved silicon transferred from various soil-plant systems towards rivers: a review. *Biogeosciences* 8, 89–112.

[B71] Cross A. F. Schlesinger W. H. (1995). A literature review and evaluation of the. *Hedley fractionation*: applications to the biogeochemical cycle of soil phosphorus in natural ecosystems. *Geoderma* 64 197–214. 10.1016/0016-7061(94)00023-4

[B72] Cruz A. F. Ishii T. (2012). Arbuscular mycorrhizal fungal spores host bacteria that affect nutrient biodynamics and biocontrol of soil-borne plant pathogens. *Biol. Open* 1 52–57. 10.1242/bio.2011014 23213368 PMC3507164

[B73] Dakora F. D. Phillips D. A. (2002). Root exudates as mediators of mineral acquisition in low-nutrient environments. *Plant Soil* 245 35–47.

[B74] Das P. Manna I. Sil P. Bandyopadhyay M. Biswas A. K. (2019). Exogenous silicon alters organic acid production and enzymatic activity of TCA cycle in two NaCl stressed indica rice cultivars. *Plant Physiol. Biochem.* 136 76–91. 10.1016/j.plaphy.2018.12.026 30658287

[B75] Das S. Mandal S. Chakrabarty A. N. Dastidar S. G. (1992). Metabolism of silicon as a probable pathogenicity factor for Mycobacterium & *Nocardia spp*. *Indian J. Med. Res.* 95 59–65. 1601470

[B76] Datnoff L. E. Snyder G. H. Korndörfer G. H. (2001). *Silicon in Agriculture.* Amsterdam:Elsevier.

[B77] de La Vega O. M. Guevara-Garcia A. Herrera-Estrella L. (2000). Enhanced phosphorus uptake in transgenic tobacco plants that overproduce citrate. *Nat. Biotechnol.* 18 450–453. 10.1038/74531 10748530

[B78] De Nobili M. Contin M. Mondini C. Brookes P. (2001). Soil microbial biomass is triggered into activity by trace amounts of substrate. *Soil Biol. Biochem.* 33 1163–1170. 10.1016/s0038-0717(01)00020-7

[B79] Demoling F. Figueroa D. Bååth E. (2007). Comparison of factors limiting bacterial growth in different soils. *Soil Biol. Biochem.* 39 2485–2495. 10.1016/j.soilbio.2007.05.002

[B80] Derry L. A. Kurtz A. C. Ziegler K. Chadwick O. A. (2005). Biological control of terrestrial silica cycling and export fluxes to watersheds. *Nature* 433 728–731. 10.1038/nature03299 15716949

[B81] Dinkelaker B. Marschner H. (1992). In vivo demonstration of acid phosphatase activity in the rhizosphere of soil-grown plants. *Plant Soil* 144 199–205. 10.1007/bf00012876

[B82] Dobbelaere S. Croonenborghs A. Thys A. Broek A. V. Vanderleyden J. (1999). Phytostimulatory effect of *Azospirillum brasilense* wild type and mutant strains altered in IAA production on wheat. *Plant Soil* 212 153–162.

[B83] Dobbelaere S. Vanderleyden J. Okon Y. (2003). Plant growth-promoting effects of diazotrophs in the rhizosphere. *Crit. Rev. Plant Sci.* 22 107–149. 10.1080/713610853

[B84] Dragišić Maksimović J. Bogdanović J. Maksimović V. Nikolic M. (2007). Silicon modulates the metabolism and utilization of phenolic compounds in cucumber (*Cucumis sativus* L.) grown at excess manganese. *J. Plant Nutrition Soil Sci.* 170 739–744. 10.1002/jpln.200700101

[B85] Drever J. I. Stillings L. L. (1997). The role of organic acids in mineral weathering. *Colloids Surf. A: Physicochem. Eng. Aspects* 120 167–181. 10.1016/s0927-7757(96)03720-x

[B86] Drigo B. Pijl A. S. Duyts H. Kielak A. M. Gamper H. A. Houtekamer M. J. (2010). Shifting carbon flow from roots into associated microbial communities in response to elevated atmospheric CO2. *Proc. Natl. Acad. Sci. U S A.* 107 10938–10942. 10.1073/pnas.0912421107 20534474 PMC2890735

[B87] Drogue B. Combes-Meynet E. Moënne-Loccoz Y. Wisniewski-Dyé F. Prigent-Combaret C. (2013). Control of the cooperation between plant growth-promoting rhizobacteria and crops by rhizosphere signals. *Mol. Microbial Ecol. Rhizosphere* 1-2 279–293. 10.1002/9781118297674.ch27

[B88] Duff R. B. Webley D. M. (1959). 2-Ketogluconic acid as a natural chelator produced by soil bacteria. *Chem. Industry* 1329, 1376–1377.

[B89] Duff S. M. G. Sarath G. Plaxton W. C. (1994). The role of acid phosphatases in plant phosphorus metabolism. *Physiol. Plant.* 90 791–800. 10.1111/j.1399-3054.1994.tb02539.x

[B90] Dungait J. A. J. Hopkins D. W. Gregory A. S. Whitmore A. P. (2012). Soil organic matter turnover is governed by accessibility not recalcitrance. *Global Change Biol.* 18 1781–1796. 10.1111/j.1365-2486.2012.02665.x

[B91] Ehrlich H. L. Newman D. K. Kappler A. (2015). *Ehrlich’s Geomicrobiology.* Boca Raton, FL:CRC press.

[B92] Elser J. J. (2012). Phosphorus: a limiting nutrient for humanity? *Curr. Opin. Biotechnol.* 23 833–838. 10.1016/j.copbio.2012.03.001 22465489

[B93] Emami S. Alikhani H. A. Pourbabaei A. A. Etesami H. Sarmadian F. Motessharezadeh B. (2019). Effect of rhizospheric and endophytic bacteria with multiple plant growth promoting traits on wheat growth. *Environ. Sci. Pollution Res.* 26 19804–19813. 10.1007/s11356-019-05284-x 31090003

[B94] Emran M. Rashad M. Gispert M. Pardini G. (2017). Increasing soil nutrients availability and sustainability by glomalin in alkaline soils. *Agricul. Biosystems Eng.* 2 74–84.

[B95] Epstein E. (1999). Silicon. *Annu. Rev. Plant Biol.* 50 641–664. 10.1146/annurev.arplant.50.1.641 15012222

[B96] Etesami H. (2018). Can interaction between silicon and plant growth promoting rhizobacteria benefit in alleviating abiotic and biotic stresses in crop plants? *Agricul. Ecosystems Environ.* 253 98–112. 10.1016/j.agee.2017.11.007

[B97] Etesami H. (2020). “Enhanced phosphorus fertilizer use efficiency with microorganisms,” in *Nutrient Dynamics for Sustainable Crop Production.* Meena R. (eds) Singapore:Springer, 215–245. 10.1007/978-981-13-8660-2_8

[B98] Etesami H. Alikhani H. A. Hosseini H. M. (2015a). Indole-3-acetic acid (IAA) production trait, a useful screening to select endophytic and rhizosphere competent bacteria for rice growth promoting agents. *MethodsX* 2 72–78. 10.1016/j.mex.2015.02.008 26150974 PMC4487705

[B99] Etesami H. Alikhani H.A. Mirseyed Hosseini H. (2015b). “Indole-3-acetic acid and 1-Aminocyclopropane-1-Carboxylate deaminase: bacterial traits required in rhizosphere, rhizoplane and/or endophytic competence by beneficial bacteria,” in *Bacterial Metabolites in Sustainable Agroecosystem*, ed. Maheshwari D.K. (Cham:Springer International Publishing), 183–258.

[B100] Etesami H. Adl S. M. (2020). Can interaction between silicon and non–rhizobial bacteria benefit in improving nodulation and nitrogen fixation in salinity–stressed legumes? a review. *Rhizosphere* 15:100229. 10.1016/j.rhisph.2020.100229

[B101] Etesami H. Alikhani H. A. (2016a). Co-inoculation with endophytic and rhizosphere bacteria allows reduced application rates of N-fertilizer for rice plant. *Rhizosphere* 2 5–12. 10.1016/j.rhisph.2016.09.003

[B102] Etesami H. Alikhani H. A. (2016b). Rhizosphere and endorhiza of oilseed rape (*Brassica napus* L.) plant harbor bacteria with multifaceted beneficial effects. *Biol. Control* 94 11–24. 10.1016/j.biocontrol.2015.12.003

[B103] Etesami H. Glick B. R. (2020). Halotolerant plant growth–promoting bacteria: prospects for alleviating salinity stress in plants. *Environ. Exp. Bot.* 178:104124. 10.1016/j.envexpbot.2020.104124

[B104] Etesami H. Jeong B. R. (2018). Silicon (Si): review and future prospects on the action mechanisms in alleviating biotic and abiotic stresses in plants. *Ecotoxicol. Environ. Saf* 147 881–896. 10.1016/j.ecoenv.2017.09.063 28968941

[B105] Etesami H. Jeong B. R. (2020). Importance of silicon in fruit nutrition: agronomic and physiological implications. *Fruit Crops* 2020 255–277. 10.1016/b978-0-12-818732-6.00019-8

[B106] Etesami H. Maheshwari D. K. (2018). Use of plant growth promoting rhizobacteria (PGPRs) with multiple plant growth promoting traits in stress agriculture: action mechanisms and future prospects. *Ecotoxicol. Environ. Saf.* 156 225–246. 10.1016/j.ecoenv.2018.03.013 29554608

[B107] Etesami H. Noori F. (2019). “Soil salinity as a challenge for sustainable agriculture and bacterial-mediated alleviation of salinity stress in crop plants,” in *Saline Soil-based Agriculture by Halotolerant Microorganisms.* (eds) Kumar M. Etesami H. Kumar V. (Singapore:Springer).

[B108] Etesami H. Hosseini H. M. Alikhani H. A. Mohammadi L. (2014). Bacterial biosynthesis of 1-aminocyclopropane-1-carboxylate (ACC) deaminase and indole-3-acetic acid (IAA) as endophytic preferential selection traits by rice plant seedlings. *J. Plant Growth Regulat.* 33 654–670. 10.1007/s00344-014-9415-3

[B109] Etesami H. Jeong B. R. Rizwan M. (2020). The use of silicon in stressed agriculture management: action mechanisms and future prospects. *Metalloids Plants: Adv. Future Prospects*, 381–431. 10.1002/9781119487210.ch19

[B110] Ezawa T. Saito K. (2018). How do arbuscular mycorrhizal fungi handle phosphate? new insight into fine-tuning of phosphate metabolism. *New Phytol.* 220 1116–1121. 29701874 10.1111/nph.15187

[B111] Fang Z. Shao C. Meng Y. Wu P. Chen M. (2009). Phosphate signaling in *Arabidopsis* and *Oryza sativa*. *Plant Sci.* 176 170–180.

[B112] Farhat M. B. Farhat A. Bejar W. Kammoun R. Bouchaala K. Fourati A. (2009). Characterization of the mineral phosphate solubilizing activity of *Serratia marcescens* CTM 50650 isolated from the phosphate mine of Gafsa. *Arch. Microbiol.* 191 815–824. 10.1007/s00203-009-0513-8 19771411

[B113] Feng G. Song Y. C. Li X. L. Christie P. (2003). Contribution of arbuscular mycorrhizal fungi to utilization of organic sources of phosphorus by red clover in a calcareous soil. *Appl. Soil Ecol.* 22 139–148. 10.1016/s0929-1393(02)00133-6

[B114] Fiorilli V. Lanfranco L. Bonfante P. (2013). The expression of GintPT, the phosphate transporter of *Rhizophagus irregularis*, depends on the symbiotic status and phosphate availability. *Planta* 237 1267–1277. 10.1007/s00425-013-1842-z 23361889

[B115] Foo E. Mcadam E. L. Weller J. L. Reid J. B. (2016). Interactions between ethylene, gibberellins, and brassinosteroids in the development of rhizobial and mycorrhizal symbioses of pea. *J. Exp. Bot.* 67 2413–2424. 10.1093/jxb/erw047 26889005 PMC4809293

[B116] Foo E. Ross J. J. Jones W. T. Reid J. B. (2013). Plant hormones in arbuscular mycorrhizal symbioses: an emerging role for gibberellins. *Ann. Bot.* 111 769–779. 10.1093/aob/mct041 23508650 PMC3631329

[B117] Frew A. Powell J. R. Allsopp P. G. Sallam N. Johnson S. N. (2017). Arbuscular mycorrhizal fungi promote silicon accumulation in plant roots, reducing the impacts of root herbivory. *Plant Soil* 419 423–433. 10.1007/s11104-017-3357-z

[B118] Frey-Klett P. Garbaye J.A. Tarkka M. (2007). The mycorrhiza helper bacteria revisited. *New Phytol.* 176 22–36. 10.1111/j.1469-8137.2007.02191.x 17803639

[B119] Gahan J. Schmalenberger A. (2015). Arbuscular mycorrhizal hyphae in grassland select for a diverse and abundant hyphospheric bacterial community involved in sulfonate desulfurization. *Appl. Soil Ecol.* 89 113–121. 10.1016/j.apsoil.2014.12.008

[B120] Gamalero E. Berta G. Massa N. Glick B. R. Lingua G. (2008). Synergistic interactions between the ACC deaminase-producing bacterium *Pseudomonas putida* UW4 and the AM fungus *Gigaspora rosea* positively affect cucumber plant growth. *FEMS Microbiol. Ecol.* 64 459–467. 10.1111/j.1574-6941.2008.00485.x 18400004

[B121] Gamalero E. Lingua G. Berta G. Glick B. R. (2009). Beneficial role of plant growth promoting bacteria and arbuscular mycorrhizal fungi on plant responses to heavy metal stress. *Can. J. Microbiol.* 55 501–514. 10.1139/w09-010 19483778

[B122] Garg N. Bhandari P. (2016a). Interactive effects of silicon and arbuscular mycorrhiza in modulating ascorbate-glutathione cycle and antioxidant scavenging capacity in differentially salt-tolerant *Cicer arietinum* L. genotypes subjected to long-term salinity. *Protoplasma* 253 1325–1345. 10.1007/s00709-015-0892-4 26468060

[B123] Garg N. Bhandari P. (2016b). Silicon nutrition and mycorrhizal inoculations improve growth, nutrient status, K+/Na+ ratio and yield of *Cicer arietinum* L. genotypes under salinity stress. *Plant Growth Regulat.* 78 371–387. 10.1007/s10725-015-0099-x

[B124] Garg N. Pandey R. (2015). Effectiveness of native and exotic arbuscular mycorrhizal fungi on nutrient uptake and ion homeostasis in salt-stressed *Cajanus cajan* L.(Millsp.) genotypes. *Mycorrhiza* 25 165–180. 10.1007/s00572-014-0600-9 25155616

[B125] Garg N. Singh S. (2018). Arbuscular mycorrhiza rhizophagus irregularis and silicon modulate growth, proline biosynthesis and yield in *Cajanus cajan* L. Millsp. (pigeonpea) genotypes under cadmium and zinc stress. *J. Plant Growth Regulat.* 37 46–63. 10.1007/s00344-017-9708-4

[B126] Garg N. Singla P. (2015). Naringenin-and Funneliformis mosseae-mediated alterations in redox state synchronize antioxidant network to alleviate oxidative stress in *Cicer arietinum* L. genotypes under salt stress. *J. Plant Growth Regulat.* 34 595–610. 10.1007/s00344-015-9494-9

[B127] Gaume A. Mächler F. De León C. Narro L. Frossard E. (2001). Low-P tolerance by maize (*Zea mays* L.) genotypes: significance of root growth, and organic acids and acid phosphatase root exudation. *Plant Soil* 228 253–264.

[B128] Gaur A. C. (2003). “Microbial mineral phosphate solubilization-an over view,” in *National Symposium On Mineral Phosphate Solubilization*, Vol. 1, 1–3.

[B129] Gaxiola R. A. Edwards M. Elser J. J. (2011). A transgenic approach to enhance phosphorus use efficiency in crops as part of a comprehensive strategy for sustainable agriculture. *Chemosphere* 84 840–845. 10.1016/j.chemosphere.2011.01.062 21367444

[B130] Gbongue L. -R. Lalaymia I. Zeze A. Delvaux B. Declerck S. (2019). Increased silicon acquisition in bananas colonized by *Rhizophagus irregularis* MUCL 41833 reduces the incidence of *Pseudocercospora fijiensis*. *Front. Plant Sci.* 9:1977. 10.3389/fpls.2018.01977 30687370 PMC6334260

[B131] Gerke J. Beißner L. Römer W. (2000). The quantitative effect of chemical phosphate mobilization by carboxylate anions on P uptake by a single root. I. the basic concept and determination of soil parameters. *J. Plant Nutrition Soil Sci.* 163 207–212. 10.1002/(sici)1522-2624(200004)163:2<207::aid-jpln207>3.0.co;2-p

[B132] Ghorchiani M. Etesami H. Alikhani H. A. (2018). Improvement of growth and yield of maize under water stress by co-inoculating an arbuscular mycorrhizal fungus and a plant growth promoting rhizobacterium together with phosphate fertilizers. *Agricul. Ecosystems Environ.* 258 59–70. 10.1016/j.agee.2018.02.016

[B133] Gianinazzi-Pearson V. (1996). Plant cell responses to arbuscular mycorrhizal fungi: getting to the roots of the symbiosis. *Plant Cell* 8:1871. 10.2307/3870236 PMC161321 12239368

[B134] Gill M. A. Ahmad Z. (2003). Inter-varietal differences of absorbed-phosphorus utilization in cotton exposed to P-free nutrition: part II. P-absorption and remobilization in plant. *Pakistan J. Sci. Res. (Pakistan)* 55 10–14

[B135] Gilroy S. Jones D. L. (2000). Through form to function: root hair development and nutrient uptake. *Trends Plant Sci.* 5 56–60. 10.1016/s1360-1385(99)01551-4 10664614

[B136] Gladkova K. F. (1982). The role of silicon in phosphate plant nutrition. *Agrochemistry* 2:133.

[B137] Glick B. R. (2012). Plant growth-promoting bacteria: mechanisms and applications. *Scientifica* 2012:963401. 10.6064/2012/963401 PMC3820493 24278762

[B138] Glick B. R. Cheng Z. Czarny J. Duan J. (2007). Promotion of plant growth by ACC deaminase-producing soil bacteria. *Eur. J. Plant Pathol.* 119 329–339. 10.1007/978-1-4020-6776-1_8

[B139] Goldstein A. H. (2007). “Future trends in research on microbial phosphate solubilization: one hundred years of insolubility”, in: *First International Meeting on Microbial Phosphate Solubilization* eds Velázquez E. Rodríguez-Barrueco C. (Dordrecht:Springer), 91–96. 10.1007/978-1-4020-5765-6_11

[B140] Gomez-Ariza J. Balestrini R. Novero M. Bonfante P. (2009). Cell-specific gene expression of phosphate transporters in mycorrhizal tomato roots. *Biol. Fertility Soils* 45 845–853. 10.1007/s00374-009-0399-2

[B141] Gonzàlez-Meler M. A. Giles L. Thomas R. B. Siedow J. N. (2001). Metabolic regulation of leaf respiration and alternative pathway activity in response to phosphate supply. *Plant Cell Environ.* 24 205–215. 10.1111/j.1365-3040.2001.00674.x

[B142] Gorbushina A. A. Broughton W. J. (2009). Microbiology of the atmosphere-rock interface: how biological interactions and physical stresses modulate a sophisticated microbial ecosystem. *Annu. Rev. Microbiol.* 63 431–450. 10.1146/annurev.micro.091208.073349 19575564

[B143] Gordienko A. S. Kurdish I. K. (2007). Surface electrical properties of *Bacillus subtilis* cells and the effect of interaction with silicon dioxide particles. *Biophysics* 52 217–219. 10.1134/s0006350907020121 17477060

[B144] Greger M. Landberg T. Vaculík M. (2018). Silicon influences soil availability and accumulation of mineral nutrients in various plant species. *Plants* 7:41. 10.3390/plants7020041 29783754 PMC6027514

[B145] Grierson P. F. (1992). Organic acids in the rhizosphere of Banksia integrifolia Lf. *Plant Soil* 144 259–265. 10.1007/bf00012883

[B146] Gu J. -Y. Zang S. -G. Sheng X. -F. He L. -Y. Huang Z. Wang Q. (2015). Burkholderia susongensis sp. nov., a mineral-weathering bacterium isolated from weathered rock surface. *Int. J. Systematic Evol. Microbiol.* 65 1031–1037. 10.1099/ijs.0.000059 25575828

[B147] Gu M. Chen A. Sun S. Xu G. (2016). Complex regulation of plant phosphate transporters and the gap between molecular mechanisms and practical application: what is missing? *Mol. Plant* 9 396–416. 10.1016/j.molp.2015.12.012 26714050

[B148] Guidry M. W. Mackenzie F. T. (2003). Experimental study of igneous and sedimentary apatite dissolution: control of pH, distance from equilibrium, and temperature on dissolution rates. *Geochimica et Cosmochimica Acta* 67 2949–2963. 10.1016/s0016-7037(03)00265-5

[B149] Guntzer F. Keller C. Meunier J. -D. (2012). Benefits of plant silicon for crops: a review. *Agronomy Sustainable Dev.* 32 201–213. 10.1007/s13593-011-0039-8

[B150] Gutjahr C. (2014). Phytohormone signaling in arbuscular mycorhiza development. *Curr. Opin. Plant Biol.* 20 26–34. 10.1016/j.pbi.2014.04.003 24853646

[B151] Gyaneshwar P. Kumar G. N. Parekh L. J. Poole P. S. (2002). Role of soil microorganisms in improving P nutrition of plants. *Plant Soil* 245 83–93.

[B152] Hajiboland R. (2012). “Effect of micronutrient deficiencies on plants stress responses,” in *Abiotic Stress Responses in Plants.* eds Ahmad P. Prasad M. (New York, NY:Springer), 283–329. 10.1007/978-1-4614-0634-1_16

[B153] Hajiboland R. Bahrami-Rad S. Poschenrieder C. (2017). Silicon modifies both a local response and a systemic response to mechanical stress in tobacco leaves. *Biol. Plantarum* 61 187–191. 10.1007/s10535-016-0633-3

[B154] Hajiboland R. Moradtalab N. Aliasgharzad N. Eshaghi Z. Feizy J. (2018). Silicon influences growth and mycorrhizal responsiveness in strawberry plants. *Physiol. Mol. Biol. Plants* 24 1103–1115. 10.1007/s12298-018-0533-4 30425427 PMC6214425

[B155] Hamayun M. Khan S. A. Khan A. L. Rehman G. Kim Y. -H. Iqbal I. (2010). Gibberellin production and plant growth promotion from pure cultures of *Cladosporium sp*. MH-6 isolated from cucumber (*Cucumis sativus* L.). *Mycologia* 102 989–995. 10.3852/09-261 20943499

[B156] Hamdali H. Bouizgarne B. Hafidi M. Lebrihi A. Virolle M. J. Ouhdouch Y. (2008). Screening for rock phosphate solubilizing actinomycetes from moroccan phosphate mines. *Appl. Soil Ecol.* 38 12–19. 10.1016/j.apsoil.2007.08.007

[B157] Hameeda B. Reddy Y. H. K. Rupela O. P. Kumar G. N. Reddy G. (2006). Effect of carbon substrates on rock phosphate solubilization by bacteria from composts and macrofauna. *Curr. Microbiol.* 53 298–302. 10.1007/s00284-006-0004-y 16941242

[B158] Hammer E. C. Nasr H. Pallon J. Olsson P. A. Wallander H. (2011). Elemental composition of arbuscular mycorrhizal fungi at high salinity. *Mycorrhiza* 21 117–129. 10.1007/s00572-010-0316-4 20499112

[B159] Hassan S. Mathesius U. (2012). The role of flavonoids in root–rhizosphere signalling: opportunities and challenges for improving plant–microbe interactions. *J. Exp. Bot.* 63 3429–3444. 10.1093/jxb/err430 22213816

[B160] Hause B. Fester T. (2005). Molecular and cell biology of arbuscular mycorrhizal symbiosis. *Planta* 221 184–196. 10.1007/s00425-004-1436-x 15871030

[B161] Hayat R. Ali S. Amara U. Khalid R. Ahmed I. (2010). Soil beneficial bacteria and their role in plant growth promotion: a review. *Annals Microbiol.* 60 579–598. 10.1007/s13213-010-0117-1

[B162] Haynes R. J. (2014). A contemporary overview of silicon availability in agricultural soils. *J. Plant Nutrition Soil Sci.* 177 831–844. 10.1002/jpln.201400202

[B163] He L. Y. Zhang Y. F. Ma H. Y. Chen Z. J. Wang Q. Y. Qian M. (2010). Characterization of copper-resistant bacteria and assessment of bacterial communities in rhizosphere soils of copper-tolerant plants. *Appl. Soil Ecol.* 44 49–55. 10.1016/j.apsoil.2009.09.004

[B164] Herman D. J. Firestone M. K. Nuccio E. Hodge A. (2012). Interactions between an arbuscular mycorrhizal fungus and a soil microbial community mediating litter decomposition. *FEMS Microbiol. Ecol.* 80 236–247. 10.1111/j.1574-6941.2011.01292.x 22224699

[B165] Herrera-Medina M. J. Steinkellner S. Vierheilig H. Ocampo Bote J. A. García Garrido J. M. (2007). Abscisic acid determines arbuscule development and functionality in the tomato arbuscular mycorrhiza. *New Phytol.* 175 554–564. 10.1111/j.1469-8137.2007.02107.x 17635230

[B166] Heuck C. Weig A. Spohn M. (2015). Soil microbial biomass C: N: P stoichiometry and microbial use of organic phosphorus. *Soil Biol. Biochem.* 85 119–129. 10.1016/j.soilbio.2015.02.029

[B167] Hildebrandt U. Janetta K. Bothe H. (2002). Towards growth of arbuscular mycorrhizal fungi independent of a plant host. *Appl. Environ. Microbiol.* 68 1919–1924. 10.1128/aem.68.4.1919-1924.2002 11916713 PMC123902

[B168] Hingston F. (1972). The role of the proton in determining adsorption envelopes. *J. Soil Sci.* 23 175–191.

[B169] Hinsinger P. (2001). Bioavailability of soil inorganic P in the rhizosphere as affected by root-induced chemical changes: a review. *Plant Soil* 237 173–195.

[B170] Hodge A. (2014). Interactions between arbuscular mycorrhizal fungi and organic material substrates. *Adv. Appl. Microbiol.* 89 47–99. 10.1016/b978-0-12-800259-9.00002-0 25131400

[B171] Hodge A. Fitter A. H. (2010). Substantial nitrogen acquisition by arbuscular mycorrhizal fungi from organic material has implications for N cycling. *Proc. Natl. Acad. Sci. U S A.* 107 13754–13759. 10.1073/pnas.1005874107 20631302 PMC2922220

[B172] Hodge A. Berta G. Doussan C. Merchan F. Crespi M. (2009). Plant root growth, architecture and function. *Plant Soil* 321 153–187. 10.1007/s11104-009-9929-9

[B173] Hodge A. Campbell C. D. Fitter A. H. (2001). An arbuscular mycorrhizal fungus accelerates decomposition and acquires nitrogen directly from organic material. *Nature* 413 297–299. 10.1038/35095041 11565029

[B174] Holford I. C. R. (1997). Soil phosphorus: its measurement, and its uptake by plants. *Soil Res.* 35 227–240. 10.1071/s96047

[B175] Hooker J. E. Piatti P. Cheshire M. V. Watson C. A. (2007). Polysaccharides and monosaccharides in the hyphosphere of the arbuscular mycorrhizal fungi Glomus E3 and Glomus tenue. *Soil Biol. Biochem.* 39 680–683. 10.1016/j.soilbio.2006.08.006

[B176] Hordiienko A. S. Samchuk A. I. Kurdysh I. K. (2010). Influence of silicon dioxide and saponite on growth of *Bacillus subtilis* IMV B-7023. *Mikrobiolohichnyi Zhurnal* 72 33–39. 20812508

[B177] Huang Z. He L. Sheng X. He Z. (2013). Weathering of potash feldspar by *Bacillus sp*. L11. *Wei sheng wu xue bao* 53 1172–1178. 24617258

[B178] Hutchens E. Valsami-Jones E. Mceldowney S. Gaze W. Mclean J. (2003). The role of heterotrophic bacteria in feldspar dissolution–an experimental approach. *Mineral. Magazine* 67 1157–1170. 10.1180/0026461036760155

[B179] Iffis B. St-Arnaud M. Hijri M. (2016). Petroleum hydrocarbon contamination, plant identity and arbuscular mycorrhizal fungal (AMF) community determine assemblages of the AMF spore-associated microbes. *Environ. Microbiol.* 18 2689–2704. 10.1111/1462-2920.13438 27376781

[B180] Illmer P. Barbato A. Schinner F. (1995). Solubilization of hardly-soluble AlPO 4 with P-solubilizing microorganisms. *Soil Biol. Biochem.* 27 265–270. 10.1016/0038-0717(94)00205-f

[B181] Iqbal M. A. Khalid M. Shahzad S. M. Ahmad M. Soleman N. Akhtar N. (2012). Integrated use of Rhizobium leguminosarum, plant growth promoting rhizobacteria and enriched compost for improving growth, nodulation and yield of lentil (*Lens culinaris* Medik.). *Chilean J. Agricul. Res.* 72:104. 10.4067/s0718-5839201200010001727315006

[B182] Jäderlund L. Arthurson V. Granhall U. Jansson J. K. (2008). Specific interactions between arbuscular mycorrhizal fungi and plant growth-promoting bacteria: as revealed by different combinations. *FEMS Microbiol. Lett.* 287 174–180. 10.1111/j.1574-6968.2008.01318.x 18754788

[B183] Jakobsen I. Abbott L. K. Robson A. D. (1992). External hyphae of vesicular-arbuscular mycorrhizal fungi associated with *Trifolium subterraneum* L. 1. spread of hyphae and phosphorus inflow into roots. *New Phytol.* 120 371–380. 10.1111/j.1469-8137.1992.tb01077.x

[B184] Jakobsen I. Gazey C. Abbott L. K. (2001). Phosphate transport by communities of arbuscular mycorrhizal fungi in intact soil cores. *New Phytol.* 149 95–103. 10.1046/j.1469-8137.2001.00006.x 33853235

[B185] James E. K. Gyaneshwar P. Mathan N. Barraquio W. L. Reddy P. M. Iannetta P. P. M. (2002)). Infection and colonization of rice seedlings by the plant growth-promoting bacterium *Herbaspirillum seropedicae* Z 67. *Mol. Plant Microbiol. Interact* 15 894–906. 10.1094/mpmi.2002.15.9.894 12236596

[B186] Janos D. P. (2007). Plant responsiveness to mycorrhizas differs from dependence upon mycorrhizas. *Mycorrhiza* 17 75–91. 10.1007/s00572-006-0094-1 17216499

[B187] Jansa J. Bukovská P. Gryndler M. (2013). Mycorrhizal hyphae as ecological niche for highly specialized hypersymbionts – or just soil free-riders? *Front. Plant Sci.* 4:134. 10.3389/fpls.2013.00134 23720665 PMC3655320

[B188] Jansa J. Finlay R. Wallander H. Smith F. A. Smith S. E. (2011). “Role of mycorrhizal symbioses in phosphorus cycling,” in *Phosphorus in Action.* eds Bünemann E. Oberson A. Frossard E. (Berlin:Springer), 137–168. 10.1007/978-3-642-15271-9_6

[B189] Jeffries P. Gianinazzi S. Perotto S. Turnau K. Barea J. -M. (2003). The contribution of arbuscular mycorrhizal fungi in sustainable maintenance of plant health and soil fertility. *Biol. Fertility Soils* 37 1–16. 10.1007/s00374-002-0546-5

[B190] Jia H. Ren H. Gu M. Zhao J. Sun S. Zhang X. (2011). The phosphate transporter gene OsPht1; 8 is involved in phosphate homeostasis in rice. *Plant Physiol.* 156 1164–1175. 10.1104/pp.111.175240 21502185 PMC3135946

[B191] Jianfeng M. A. Takahashi E. (1991). Effect of silicate on phosphate availability for rice in a P-deficient soil. *Plant Soil* 133 151–155. 10.1007/bf00009187

[B192] Johansson J. F. Paul L. R. Finlay R. D. (2004). Microbial interactions in the mycorrhizosphere and their significance for sustainable agriculture. *FEMS Microbiol. Ecol.* 48 1–13. 10.1016/j.femsec.2003.11.012 19712426

[B193] Johnson S. E. Loeppert R. H. (2006). Role of organic acids in phosphate mobilization from iron oxide. *Soil Sci. Soc. Am. J.* 70 222–234. 10.2136/sssaj2005.0012

[B194] Johri A. K. Oelmüller R. Dua M. Yadav V. Kumar M. Tuteja N. (2015). Fungal association and utilization of phosphate by plants: success, limitations, and future prospects. *Front. Microbiol.* 6:984. 10.3389/fmicb.2015.00984 26528243 PMC4608361

[B195] Joner E. J. Jakobsen I. (1995). Growth and extracellular phosphatase activity of arbuscular mycorrhizal hyphae as influenced by soil organic matter. *Soil Biol. Biochem.* 27 1153–1159. 10.1016/0038-0717(95)00047-i

[B196] Joner E. J. Van Aarle I. M. Vosatka M. (2000). Phosphatase activity of extra-radical arbuscular mycorrhizal hyphae: a review. *Plant Soil* 226 199–210.

[B197] Jones D. L. Oburger E. (2011). “Solubilization of phosphorus by soil microorganisms,” in *Phosphorus in Action.* Eds Bunemann E. K. Oberson A. Frossard E. (Berlin:Springer), 169–198. 10.1007/978-3-642-15271-9_7

[B198] Jones D. Dennis P. Owen A. Van Hees P. (2003). Organic acid behavior in soils–misconceptions and knowledge gaps. *Plant Soil* 248 31–41. 10.1007/978-94-010-0243-1_3

[B199] Jongmans A. G. Van Breemen N. Lundström U. Van Hees P. A. W. Finlay R. D. Srinivasan M. (1997). Rock-eating fungi. *Nature* 389 682–683.

[B200] Jorquera M. A. Hernández M. T. Rengel Z. Marschner P. De La Luz Mora M. (2008). Isolation of culturable phosphobacteria with both phytate-mineralization and phosphate-solubilization activity from the rhizosphere of plants grown in a volcanic soil. *Biol. Fertility Soils* 44:1025. 10.1007/s00374-008-0288-0

[B201] Kaiser C. Kilburn M. R. Clode P. L. Fuchslueger L. Koranda M. Cliff J. B. (2015). Exploring the transfer of recent plant photosynthates to soil microbes: mycorrhizal pathway vs direct root exudation. *New Phytol.* 205 1537–1551. 10.1111/nph.13138 25382456 PMC4357392

[B202] Kaldorf M. Ludwig-Müller J. (2000). AM fungi might affect the root morphology of maize by increasing indole-3-butyric acid biosynthesis. *Physiol. Plant* 109 58–67. 10.1034/j.1399-3054.2000.100109.x11841302

[B203] Kalinowski B. E. Liermann L. J. Brantley S. L. Barnes A. Pantano C. G. (2000). X-ray photoelectron evidence for bacteria-enhanced dissolution of hornblende. *Geochim. Cosmochimica Acta* 64 1331–1343. 10.1016/s0016-7037(99)00371-3

[B204] Kang S. -M. Khan A. L. Hamayun M. Hussain J. Joo G. -J. You Y. -H. (2012). Gibberellin-producing Promicromonospora sp. SE188 improves *Solanum lycopersicum* plant growth and influences endogenous plant hormones. *J. Microbiol.* 50 902–909. 10.1007/s12275-012-2273-4 23274975

[B205] Kang S. -M. Waqas M. Shahzad R. You Y. -H. Asaf S. Khan M. A. (2017). Isolation and characterization of a novel silicate-solubilizing bacterial strain Burkholderia eburnea CS4-2 that promotes growth of japonica rice (*Oryza sativa* L. cv. Dongjin). *Soil Sci. Plant Nutrition* 63 233–241.

[B206] Karimzadeh J. Alikhani H. A. Etesami H. Pourbabaei A. A. (2020). Improved phosphorus uptake by wheat plant (*Triticum aestivum* L.) with rhizosphere fluorescent pseudomonads strains under water-deficit stress. *J. Plant Growth Regulation* 40 162–178 10.1007/s00344-020-10087-3

[B207] Karthikeyan A. S. Varadarajan D. K. Mukatira U. T. D’urzo M. P. Damsz B. Raghothama K. G. (2002). Regulated expression of *Arabidopsis* phosphate transporters. *Plant Physiol.* 130 221–233. 10.1104/pp.020007 12226502 PMC166555

[B208] Karunakaran G. Suriyaprabha R. Manivasakan P. Yuvakkumar R. Rajendran V. Prabu P. (2013). Effect of nanosilica and silicon sources on plant growth promoting rhizobacteria, soil nutrients and maize seed germination. *IET Nanobiotechnol.* 7 70–77. 10.1049/iet-nbt.2012.0048 24028804

[B209] Kaufmian P. B. Bigelow W. C. Petering L. B. Drogosz F. B. (1969). Silica in developing epidermal cells of Avena internodes: electron microprobe analysis. *Science* 166 1015–1017. 10.1126/science.166.3908.1015 17758067

[B210] Khan M. S. Zaidi A. Wani P. A. (2007). Role of phosphate-solubilizing microorganisms in sustainable agriculture—a review. *Agronomy Sustainable Dev.* 27 29–43. 10.1051/agro:2006011

[B211] Khan M. S. Zaidi A. Wani P. A. Oves M. (2009). Role of plant growth promoting rhizobacteria in the remediation of metal contaminated soils. *Environ. Chem. Lett.* 7 1–19. 10.1007/s10311-008-0155-0

[B212] Kihara T. Wada T. Suzuki Y. Hara T. Koyama H. (2003). Alteration of citrate metabolism in cluster roots of white lupin. *Plant Cell Physiol.* 44 901–908. 10.1093/pcp/pcg115 14519771

[B213] Kim K. Y. Jordan D. Mcdonald G. A. (1997). Effect of phosphate-solubilizing bacteria and vesicular-arbuscular mycorrhizae on tomato growth and soil microbial activity. *Biol. Fertility Soils* 26 79–87. 10.1007/s003740050347

[B214] Kim Y. H. Khan A. L. Waqas M. Shim J. K. Kim D. H. Lee K. Y. (2014). Silicon application to rice root zone influenced the phytohormonal and antioxidant responses under salinity stress. *J. Plant Growth Regulation* 33 137–149. 10.1007/s00344-013-9356-2

[B215] Klotzbücher T. Marxen A. Vetterlein D. Schneiker J. Türke M. Van Sinh N. (2015). Plant-available silicon in paddy soils as a key factor for sustainable rice production in Southeast Asia. *Basic Appl. Ecol.* 16 665–673. 10.1016/j.baae.2014.08.002

[B216] Klugh K. R. Cumming J. R. (2007). Variations in organic acid exudation and aluminum resistance among arbuscular mycorrhizal species colonizing *Liriodendron tulipifera*. *Tree Physiol.* 27 1103–1112. 10.1093/treephys/27.8.1103 17472937

[B217] Klugh-Stewart K. Cumming J. R. (2009). Organic acid exudation by mycorrhizal *Andropogon virginicus* L.(broomsedge) roots in response to aluminum. *Soil Biol. Biochem.* 41 367–373. 10.1016/j.soilbio.2008.11.013

[B218] Koide R. T. Kabir Z. (2000). Extraradical hyphae of the mycorrhizal fungus Glomus intraradices can hydrolyse organic phosphate. *New Phytol.* 148 511–517. 10.1046/j.1469-8137.2000.00776.x 33863024

[B219] Konhauser K. O. Lalonde S. V. Amskold L. Holland H. D. (2007). Was there really an archean phosphate crisis? *Science* 315 1234–1234. 10.1126/science.1136328 17332403

[B220] Koski-Vähälä J. Hartikainen H. Tallberg P. (2001). Phosphorus mobilization from various sediment pools in response to increased pH and silicate concentration. *J. Environ. Qual.* 30 546–552. 10.2134/jeq2001.302546x 11285916

[B221] Kostic L. Nikolic N. Bosnic D. Samardzic J. Nikolic M. (2017). Silicon increases phosphorus (P) uptake by wheat under low P acid soil conditions. *Plant Soil* 419 447–455. 10.1007/s11104-017-3364-0

[B222] Kostic L. Nikolic N. Samardzic J. Milisavljevic M. Maksimović V. Cakmak D. (2015). Liming of anthropogenically acidified soil promotes phosphorus acquisition in the rhizosphere of wheat. *Biol. Fertility Soils* 51 289–298. 10.1007/s00374-014-0975-y

[B223] Kothari S. K. Marschner H. Römheld V. (1990). Direct and indirect effects of VA mycorrhizal fungi and rhizosphere microorganisms on acquisition of mineral nutrients by maize (*Zea mays* L.) in a calcareous soil. *New Phytol.* 116 637–645. 10.1111/j.1469-8137.1990.tb00549.x

[B224] Kutuzova R. S. (1969). Release of silica from minerals as a result of microbial activity. *Mikrobiologiya* 38 596–602. 5401119

[B225] Lambers H. Plaxton W. C. (2015). Phosphorus: back to the roots. *Annual Plant Rev.* 48 3–22.

[B226] Lambers H. Brundrett M. C. Raven J. A. Hopper S. D. (2010). Plant mineral nutrition in ancient landscapes: high plant species diversity on infertile soils is linked to functional diversity for nutritional strategies. *Plant Soil* 334 11–31. 10.1007/s11104-010-0444-9

[B227] Lambers H. Shane M. W. Cramer M. D. Pearse S. J. Veneklaas E. J. (2006). Root structure and functioning for efficient acquisition of phosphorus: matching morphological and physiological traits. *Ann. Bot.* 98 693–713. 10.1093/aob/mcl114 16769731 PMC2806175

[B228] Lambrecht M. Okon Y. Broek A. V. Vanderleyden J. (2000). Indole-3-acetic acid: a reciprocal signalling molecule in bacteria–plant interactions. *Trends Microbiol.* 8 298–300. 10.1016/s0966-842x(00)01732-7 10878760

[B229] Lapanje A. Wimmersberger C. Furrer G. Brunner I. Frey B. (2012). Pattern of elemental release during the granite dissolution can be changed by aerobic heterotrophic bacterial strains isolated from Damma Glacier (central Alps) deglaciated granite sand. *Microb. Ecol.* 63 865–882. 10.1007/s00248-011-9976-7 22105516

[B230] Lecomte J. St-Arnaud M. Hijri M. (2011). Isolation and identification of soil bacteria growing at the expense of arbuscular mycorrhizal fungi. *FEMS Microbiol. Lett.* 317 43–51. 10.1111/j.1574-6968.2011.02209.x 21219415

[B231] Lee K. -E. Adhikari A. Kang S. -M. You Y. -H. Joo G. -J. Kim J. -H. (2019). Isolation and characterization of the high silicate and phosphate solubilizing novel strain *Enterobacter ludwigii* GAK2 that promotes growth in rice plants. *Agronomy* 9:144. 10.3390/agronomy9030144

[B232] Lee Y. Krishnamoorthy R. Selvakumar G. Kim K. Sa T. (2015). Alleviation of salt stress in maize plant by co-inoculation of arbuscular mycorrhizal fungi and *Methylobacterium oryzae* CBMB20. *J. Korean Soc. Appl. Biol. Chem.* 58 533–540. 10.1007/s13765-015-0072-4

[B233] Leggewie G. Willmitzer L. Riesmeier J. W. (1997). Two cDNAs from potato are able to complement a phosphate uptake-deficient yeast mutant: identification of phosphate transporters from higher plants. *Plant Cell* 9 381–392. 10.2307/3870489 9090882 PMC156925

[B234] Leigh J. Fitter A. H. Hodge A. (2011). Growth and symbiotic effectiveness of an arbuscular mycorrhizal fungus in organic matter in competition with soil bacteria. *FEMS Microbiol. Ecol.* 76 428–438. 10.1111/j.1574-6941.2011.01066.x 21303398

[B235] Liao H. Rubio G. Yan X. Cao A. Brown K. M. Lynch J. P. (2001). Effect of phosphorus availability on basal root shallowness in common bean. *Plant Soil* 232 69–79. 10.1007/978-94-010-0566-1_7 11729851

[B236] Lin Q. -M. Rao Z. -H. Sun Y. -X. Yao J. Xing L. -J. (2002). Identification and practical application of silicate-dissolving bacteria. *Agricultural Sci. China* 1 81–85.

[B237] Lin W. -P. Jiang N. -H. Peng L. Fan X. -Y. Gao Y. Wang G. -P. (2020). Silicon impacts on soil microflora under *Ralstonia solanacearum* inoculation. *J. Int. Agricul.* 19 251–264. 10.1016/s2095-3119(18)62122-7

[B238] Linderman R. G. (1992). Vesicular-arbuscular mycorrhizae and soil microbial interactions. *Mycorrhizae Sustainable Agricul.* 54, 45–70. 10.2134/asaspecpub54.c3

[B239] Liu W. Xu X. Wu X. Yang Q. Luo Y. Christie P. (2006). Decomposition of silicate minerals by Bacillus mucilaginosus in liquid culture. *Environ. Geochem. Health* 28 133–140. 10.1007/s10653-005-9022-0 16528584

[B240] López-Bucio J. Cruz-Ramırez A. Herrera-Estrella L. (2003). The role of nutrient availability in regulating root architecture. *Curr. Opin. Plant Biol.* 6 280–287. 10.1016/s1369-5266(03)00035-9 12753979

[B241] Ludwig-Müller J. (2010). “Hormonal responses in host plants triggered by arbuscular mycorrhizal fungi,” in *Arbuscular Mycorrhizas: Physiology and Function*, Koltai H. Kapulnik Y. (Berlin:Springer), 169–190.

[B242] Ludwig-Müller J. Güther M. (2007). Auxins as signals in arbuscular mycorrhiza formation. *Plant Signal. Behav.* 2 194–196. 10.4161/psb.2.3.4152 19704695 PMC2634056

[B243] Lynch J. P. (2007). Roots of the second green revolution. *Aus. J. Botany* 55 493–512. 10.1071/bt06118

[B244] Lynch J. P. (2011). Root phenes for enhanced soil exploration and phosphorus acquisition: tools for future crops. *Plant Physiol.* 156 1041–1049. 10.1104/pp.111.175414 21610180 PMC3135935

[B245] Ma J. F. (2004). Role of silicon in enhancing the resistance of plants to biotic and abiotic stresses. *Soil Sci. Plant Nutrition* 50 11–18. 10.1080/00380768.2004.10408447

[B246] Ma J. F. Takahashi E. (2002). *Soil, Fertilizer, and Plant Silicon Research in Japan.* Elsevier:Amsterdam.

[B247] Ma J. F. Tamai K. Yamaji N. Mitani N. Konishi S. Katsuhara M. (2006). A silicon transporter in rice. *Nature* 440 688–691. 16572174 10.1038/nature04590

[B248] Ma J. F. Yamaji N. Mitani N. Tamai K. Konishi S. Fujiwara T. (2007). An efflux transporter of silicon in rice. *Nature* 448 209–212. 10.1038/nature05964 17625566

[B249] Ma J. Takahashi E. (1990). Effect of silicon on the growth and phosphorus uptake of rice. *Plant Soil* 126 115–119. 10.1007/bf00041376

[B250] Mäder P. Kaiser F. Adholeya A. Singh R. Uppal H. S. Sharma A. K. (2011). Inoculation of root microorganisms for sustainable wheat–rice and wheat–black gram rotations in India. *Soil Biol. Biochem.* 43 609–619. 10.1016/j.soilbio.2010.11.031

[B251] Malinovskaya I. M. Kosenko L. V. Votselko S. K. Podgorskii V. S. (1990). Role of *Bacillus mucilaginosus* polysaccharide in degradation of silicate minerals. *Microbiology* 59 49–55.

[B252] Mandal S. M. Chakraborty D. Dey S. (2010). Phenolic acids act as signaling molecules in plant-microbe symbioses. *Plant Signal. Behav.* 5 359–368. 10.4161/psb.5.4.10871 20400851 PMC2958585

[B253] Marschner H. (1995). *Mineral Nutrition of Higher Plants* 2nd edition.Great Britain:Academic press.

[B254] Marschner H. Dell B. (1994). Nutrient uptake in mycorrhizal symbiosis. *Plant Soil* 159 89–102. 10.1007/bf00000098

[B255] Marschner H. Rimmington G. (1988). Mineral nutrition of higher plants. *Plant Cell Environ.* 11 147–148.

[B256] Marschner P. Crowley D. Lieberei R. (2001). Arbuscular mycorrhizal infection changes the bacterial 16 S rDNA community composition in the rhizosphere of maize. *Mycorrhiza* 11 297–302. 10.1007/s00572-001-0136-7 24549350

[B257] Martín-Rodríguez J. A. Huertas R. Ho-Plágaro T. Ocampo J. A. Turečková V. Tarkowská D. (2016). Gibberellin–abscisic acid balances during arbuscular mycorrhiza formation in tomato. *Front. Plant Sci.* 7:1273. 10.3389/fpls.2016.01273 27602046 PMC4993810

[B258] Martín-Rodríguez J. Á. León-Morcillo R. Vierheilig H. Ocampo J. A. Ludwig-Müller J. García-Garrido J. M. (2011). Ethylene-dependent/ethylene-independent ABA regulation of tomato plants colonized by arbuscular mycorrhiza fungi. *New Phytol.* 190 193–205. 10.1111/j.1469-8137.2010.03610.x 21232061

[B259] Martín-Rodríguez J. Á. Ocampo J. A. Molinero-Rosales N. Tarkowská D. Ruíz-Rivero O. García-Garrido J. M. (2015). Role of gibberellins during arbuscular mycorrhizal formation in tomato: new insights revealed by endogenous quantification and genetic analysis of their metabolism in mycorrhizal roots. *Physiol. Plant.* 154 66–81. 10.1111/ppl.12274 25186107

[B260] McGuiness P. N. Reid J. B. Foo E. (2019). The role of gibberellins and brassinosteroids in nodulation and arbuscular mycorrhizal associations. *Front. Plant Sci.* 10:269. 10.3389/fpls.2019.00269 30930916 PMC6429038

[B261] Meding S. M. Zasoski R. J. (2008). Hyphal-mediated transfer of nitrate, arsenic, cesium, rubidium, and strontium between arbuscular mycorrhizal forbs and grasses from a California oak woodland. *Soil Biol. Biochem.* 40 126–134. 10.1016/j.soilbio.2007.07.019

[B262] Meena V. D. Dotaniya M. L. Coumar V. Rajendiran S. Kundu S. Rao A. S. (2014). A case for silicon fertilization to improve crop yields in tropical soils. *Proc. Natl. Acad. Sci. U S A. India Section B: Biol. Sci.* 84 505–518. 10.1007/s40011-013-0270-y

[B263] Meharg C. Meharg A. A. (2015). Silicon, the silver bullet for mitigating biotic and abiotic stress, and improving grain quality, in rice? *Environ. Exp. Botany* 120 8–17. 10.1016/j.envexpbot.2015.07.001

[B264] Menezes-Blackburn D. Jorquera M. A. Gianfreda L. Greiner R. De La Luz Mora M. (2014). A novel phosphorus biofertilization strategy using cattle manure treated with phytase–nanoclay complexes. *Biol. Fertility Soils* 50 583–592.

[B265] Miao J. Sun J. Liu D. Li B. Zhang A. Li Z. (2009). Characterization of the promoter of phosphate transporter TaPHT1. 2 differentially expressed in wheat varieties. *J. Genet. Genom.* 36 455–466. 10.1016/s1673-8527(08)60135-6 19683668

[B266] Miransari M. (2010). Contribution of arbuscular mycorrhizal symbiosis to plant growth under different types of soil stress. *Plant Biol.* 12 563–569. 20636898 10.1111/j.1438-8677.2009.00308.x

[B267] Misra N. Gupta G. Jha P. N. (2012). Assessment of mineral phosphate-solubilizing properties and molecular characterization of zinc-tolerant bacteria. *J. Basic Microbiol.* 52 549–558. 10.1002/jobm.201100257 22359218

[B268] Mitani N. Ma J. F. (2005). Uptake system of silicon in different plant species. *J. Exp. Bot.* 56 1255–1261. 10.1093/jxb/eri121 15753109

[B269] Mitani-Ueno N. Yamaji N. Ma J. F. (2011). Silicon efflux transporters isolated from two pumpkin cultivars contrasting in Si uptake. *Plant Signal. Behav.* 6 991–994. 10.4161/psb.6.7.15462 21617377 PMC3257775

[B270] Mohamed A. A. Eweda W. E. E. Heggo A. M. Hassan E. A. (2014). Effect of dual inoculation with arbuscular mycorrhizal fungi and sulphur-oxidising bacteria on onion (*Allium cepa* L.) and maize (*Zea mays* L.) grown in sandy soil under green house conditions. *Annals Agricul. Sci.* 59 109–118. 10.1016/j.aoas.2014.06.015

[B271] Mohammad A. Mittra B. (2013). Effects of inoculation with stress-adapted arbuscular mycorrhizal fungus Glomus deserticola on growth of *Solanum melogena* L. and *Sorghum sudanese* Staph. seedlings under salinity and heavy metal stress conditions. *Arch. Agronomy Soil Sci.* 59 173–183. 10.1080/03650340.2011.610029

[B272] Montpetit J. Vivancos J. Mitani-Ueno N. Yamaji N. Rémus-Borel W. Belzile F. (2012). Cloning, functional characterization and heterologous expression of TaLsi1, a wheat silicon transporter gene. *Plant Mol. Biol.* 79 35–46. 10.1007/s11103-012-9892-3 22351076

[B273] Moradtalab N. Hajiboland R. Aliasgharzad N. Hartmann T. E. Neumann G. (2019). Silicon and the association with an arbuscular-mycorrhizal fungus (*Rhizophagus clarus*) mitigate the adverse effects of drought stress on strawberry. *Agronomy* 9:41. 10.3390/agronomy9010041

[B274] Nadeem S. M. Ahmad M. Zahir Z. A. Javaid A. Ashraf M. (2014). The role of mycorrhizae and plant growth promoting rhizobacteria (PGPR) in improving crop productivity under stressful environments. *Biotechnol. Adv.* 32 429–448. 10.1016/j.biotechadv.2013.12.005 24380797

[B275] Neu S. Schaller J. Dudel E. G. (2017). Silicon availability modifies nutrient use efficiency and content, C: N: P stoichiometry, and productivity of winter wheat (*Triticum aestivum* L.). *Sci. Rep.* 7:40829. 10.1038/srep40829 PMC5240101 28094308

[B276] Neumann G. Römheld V. (1999). Root excretion of carboxylic acids and protons in phosphorus-deficient plants. *Plant Soil* 211 121–130.

[B277] Niu Y. F. Chai R. S. Jin G. L. Wang H. Tang C. X. Zhang Y. S. (2013). Responses of root architecture development to low phosphorus availability: a review. *Ann. Bot.* 112 391–408. 10.1093/aob/mcs285 23267006 PMC3698383

[B278] Nogueira M. A. Cardoso E. Hampp R. (2002). Manganese toxicity and callose deposition in leaves are attenuated in mycorrhizal soybean. *Plant Soil* 246 1–10. 10.1111/j.1365-313x.2010.04399.x 21175885

[B279] Nuccio E. E. Hodge A. Pett-Ridge J. Herman D. J. Weber P. K. Firestone M. K. (2013). An arbuscular mycorrhizal fungus significantly modifies the soil bacterial community and nitrogen cycling during litter decomposition. *Environ. Microbiol.* 15 1870–1881. 10.1111/1462-2920.12081 23360621

[B280] Oburger E. Jones D. L. Wenzel W. W. (2011). Phosphorus saturation and pH differentially regulate the efficiency of organic acid anion-mediated P solubilization mechanisms in soil. *Plant Soil* 341 363–382. 10.1007/s11104-010-0650-5

[B281] Offre P. Pivato B. Siblot S. Gamalero E. Corberand T. Lemanceau P. (2007). Identification of bacterial groups preferentially associated with mycorrhizal roots of *Medicago truncatula*. *Appl. Environ. Microbiol.* 73 913–921. 10.1128/aem.02042-06 17142371 PMC1800773

[B282] Olander L. P. Vitousek P. M. (2004). Biological and geochemical sinks for phosphorus in soil from a wet tropical forest. *Ecosystems* 7 404–419.

[B283] Olsson P. A. Van Aarle I. M. Allaway W. G. Ashford A. E. Rouhier H. (2002). Phosphorus effects on metabolic processes in monoxenic arbuscular mycorrhiza cultures. *Plant Physiol.* 130 1162–1171. 10.1104/pp.009639 12427983 PMC166637

[B284] Ordoñez Y. M. Fernandez B. R. Lara L. S. Rodriguez A. Uribe-Vélez D. Sanders I. R. (2016). Bacteria with phosphate solubilizing capacity alter mycorrhizal fungal growth both inside and outside the root and in the presence of native microbial communities. *PLoS One* 11:e0154438. 10.1371/journal.pone.0154438 27253975 PMC4890779

[B285] Ordookhani K. Khavazi K. Moezzi A. Rejali F. (2010). Influence of PGPR and AMF on antioxidant activity, lycopene and potassium contents in tomato. *African J. Agricul. Res.* 5 1108–1116.

[B286] Osborne L. D. Rengel Z. (2002). Growth and P uptake by wheat genotypes supplied with phytate as the only P source. *Australian J. Agricul. Res.* 53 845–850. 10.1071/ar01102

[B287] Osorio Vega N. W. (2007). A review on beneficial effects of rhizosphere bacteria on soil nutrient availability and plant nutrient uptake. *Revista Facultad Nacional de Agronomia Medellin* 60 3621–3643.

[B288] Oueslati O. (2003). Allelopathy in two durum wheat (*Triticum durum* L.) varieties. *Agricult. Ecosystems Environ.* 96 161–163. 10.1016/s0167-8809(02)00201-3

[B289] Oves M. Khan M. S. Zaidi A. (2013). Chromium reducing and plant growth promoting novel strain *Pseudomonas aeruginosa* OSG41 enhance chickpea growth in chromium amended soils. *Eur. J. Soil Biol.* 56 72–83. 10.1016/j.ejsobi.2013.02.002

[B290] Owino-Gerroh C. Gascho G. J. (2005). Effect of silicon on low pH soil phosphorus sorption and on uptake and growth of maize. *Commun. Soil Sci. Plant Anal.* 35 2369–2378. 10.1081/lcss-200030686

[B291] Pang J. Yang J. Lambers H. Tibbett M. Siddique K. H. M. Ryan M. H. (2015). Physiological and morphological adaptations of herbaceous perennial legumes allow differential access to sources of varyingly soluble phosphate. *Physiol. Plant.* 154 511–525. 10.1111/ppl.12297 25291346

[B292] Park K. H. Lee C. Y. Son H. J. (2009). Mechanism of insoluble phosphate solubilization by *Pseudomonas fluorescens* RAF15 isolated from ginseng rhizosphere and its plant growth-promoting activities. *Lett. Appl. Microbiol.* 49 222–228. 10.1111/j.1472-765x.2009.02642.x 19486289

[B293] Parks E. J. Olson G. J. Brinckman F. E. Baldi F. (1990). Characterization by high performance liquid chromatography (HPLC) of the solubilization of phosphorus in iron ore by a fungus. *J. Industrial Microbiol.* 5 183–189. 10.1007/bf01573868

[B294] Patel D. K. Archana G. Kumar G. N. (2008). Variation in the nature of organic acid secretion and mineral phosphate solubilization by Citrobacter sp. DHRSS in the presence of different sugars. *Curr. Microbiol.* 56 168–174. 10.1007/s00284-007-9053-0 17965911

[B295] Pavlovic J. Samardzic J. Maksimović V. Timotijevic G. Stevic N. Laursen K. H. (2013). Silicon alleviates iron deficiency in cucumber by promoting mobilization of iron in the root apoplast. *New Phytol.* 198 1096–1107. 10.1111/nph.12213 23496257

[B296] Peera S. K. P. G. Balasubramaniam P. Mahendran P. P. (2016). Effect of fly ash and silicate solubilizing bacteria on yield and silicon uptake of rice in Cauvery Delta Zone. *Environ. Ecol.* 34 1966–1971.

[B297] Pepe A. Giovannetti M. Sbrana C. (2020). Appressoria and phosphorus fluxes in mycorrhizal plants: connections between soil-and plant-based hyphae. *Mycorrhiza* 30 589–600. 10.1007/s00572-020-00972-w 32533256

[B298] Perry C. C. (2003). Silicification: the processes by which organisms capture and mineralize silica. *Rev. Mineral. Geochem.* 54 291–327. 10.1515/9781501509346-015

[B299] Planavsky N. J. Rouxel O. J. Bekker A. Lalonde S. V. Konhauser K. O. Reinhard C. T. (2010). The evolution of the marine phosphate reservoir. *Nature* 467 1088–1090. 10.1038/nature09485 20981096

[B300] Plaxton W. C. (2004). *Plant Response to Stress: Biochemical Adaptations to Phosphate Deficiency. Encyclopedia of Plant and Crop Science.* Marcel Dekker,New York, 976–980.

[B301] Plaxton W. C. Carswell M. C. (1999). “Metabolic aspects of the phosphate starvation response in plants,” in *Plant Responses to Environmental Stresses: from Phytohormones to Genome Reorganization* ed Lerner H. R. (New York, NY:Marcel Dekker), 349–372. 10.1201/9780203743157-16

[B302] Pokrovsky O. Shirokova L. Stockman G. Zabelina S. Bénézeth P. Gerard E. (2011). Quantifying the role of microorganisms in silicate mineral dissolution at the conditions of CO2 storage in basalts. *Geophys. Res. Abstracts* 13:13904.

[B303] Pons S. Fournier S. Chervin C. Bécard G. Rochange S. Frei Dit Frey N. (2020). Phytohormone production by the arbuscular mycorrhizal fungus *Rhizophagus irregularis*. *PLoS One* 15:e0240886. 10.1371/journal.pone.0240886 33064769 PMC7567356

[B304] Pothier J. F. Wisniewski-Dye F. Weiss-Gayet M. Moenne-Loccoz Y. Prigent-Combaret C. (2007). Promoter-trap identification of wheat seed extract-induced genes in the plant-growth-promoting rhizobacterium *Azospirillum brasilense* Sp245. *Microbiology* 153 3608–3622. 10.1099/mic.0.2007/009381-0 17906157

[B305] Prusty R. Grisafi P. Fink G. R. (2004). The plant hormone indoleacetic acid induces invasive growth in *Saccharomyces cerevisiae*. *Proc. Natl. Acad. Sci. U S A.* 101 4153–4157. 10.1073/pnas.0400659101 15010530 PMC384710

[B306] Raghothama K. G. Karthikeyan A. S. (2005). Phosphate acquisition. *Plant Soil* 274:37.

[B307] Rajan S. S. S. (1975). Phosphate adsorption and the displacement of structural silicon in an allophane clay. *J. Soil Sci.* 26 250–256. 10.1111/j.1365-2389.1975.tb01949.x

[B308] Ramaekers L. Remans R. Rao I. M. Blair M. W. Vanderleyden J. (2010). Strategies for improving phosphorus acquisition efficiency of crop plants. *Field Crops Res.* 117 169–176. 10.1016/j.fcr.2010.03.001

[B309] Rangaraj S. Gopalu K. Rathinam Y. Periasamy P. Venkatachalam R. Narayanasamy K. (2014). Effect of silica nanoparticles on microbial biomass and silica availability in maize rhizosphere. *Biotechnol. Appl. Biochem.* 61 668–675. 10.1002/bab.1191 24329970

[B310] Rashid M. Khalil S. Ayub N. Alam S. Latif F. (2004). Organic acids production and phosphate solubilization by phosphate solubilizing microorganisms (PSM) under in vitro conditions. *Pak. J. Biol. Sci.* 7 187–196. 10.3923/pjbs.2004.187.196

[B311] Redecker D. Schüßler A. Stockinger H. Stürmer S. L. Morton J. B. Walker C. (2013). An evidence-based consensus for the classification of arbuscular mycorrhizal fungi (Glomeromycota). *Mycorrhiza* 23 515–531. 10.1007/s00572-013-0486-y 23558516

[B312] Reithmaier G. -M. S. Knorr K. -H. Arnhold S. Planer-Friedrich B. Schaller J. (2017). Enhanced silicon availability leads to increased methane production, nutrient and toxicant mobility in peatlands. *Sci. Rep.* 7: 8728. 10.1038/s41598-017-09130-3 PMC5562759 28821870

[B313] Rengel Z. Marschner P. (2005). Nutrient availability and management in the rhizosphere: exploiting genotypic differences. *New Phytol.* 168 305–312. 10.1111/j.1469-8137.2005.01558.x 16219070

[B314] Rezakhani L. Motesharezadeh B. Tehrani M. M. Etesami H. Hosseini H. M. (2019a). Effect of silicon and phosphate-solubilizing bacteria on improved phosphorus (P) uptake is not specific to insoluble P-fertilized sorghum (*Sorghum bicolor* L.) Plants. *J. Plant Growth Regulat.* 39 239–253. 10.1007/s00344-019-09978-x

[B315] Rezakhani L. Motesharezadeh B. Tehrani M. M. Etesami H. Hosseini H. M. (2019b). Phosphate–solubilizing bacteria and silicon synergistically augment phosphorus (P) uptake by wheat (*Triticum aestivum* L.) plant fertilized with soluble or insoluble P source. *Ecotoxicol. Environ. Safety* 173 504–513. 10.1016/j.ecoenv.2019.02.060 30802739

[B316] Richardson A. E. Barea J. -M. Mcneill A. M. Prigent-Combaret C. (2009). Acquisition of phosphorus and nitrogen in the rhizosphere and plant growth promotion by microorganisms. *Plant Soil* 321 305–339. 10.1007/s11104-009-9895-2

[B317] Ringeval B. Augusto L. Monod H. Apeldoorn D. Bouwman L. Yang X. (2017). Phosphorus in agricultural soils: drivers of its distribution at the global scale. *Global Change Biol.* 23 3418–3432 10.1111/gcb.13618 28067005

[B318] Rodrigues F. Á. Mcnally D. J. Datnoff L. E. Jones J. B. Labbé C. Benhamou N. (2004). Silicon enhances the accumulation of diterpenoid phytoalexins in rice: a potential mechanism for blast resistance. *Phytopathology* 94 177–183. 18943541 10.1094/PHYTO.2004.94.2.177

[B319] Rodrìguez H. Fraga R. (1999). Phosphate solubilizing bacteria and their role in plant growth promotion. *Biotechnol. Adv.* 17 319–339. 10.1016/s0734-9750(99)00014-2 14538133

[B320] Römer W. Schenk H. (1998). Influence of genotype on phosphate uptake and utilization efficiencies in spring barley. *Eur. J. Agronomy* 8 215–224. 10.1016/s1161-0301(97)00061-0

[B321] Sahebi M. Hanafi M. M. Siti nor Akmar A. Rafii M. Y. Azizi P. Tengoua F. F. (2015). Importance of silicon and mechanisms of biosilica formation in plants. *BioMed Res. Int.* 2015:396010. 10.1155/2015/396010 PMC4317640 25685787

[B322] Sánchez-Calderón L. López-Bucio J. Chacón-López A. Gutiérrez-Ortega A. Hernández-Abreu E. Herrera-Estrella L. (2006). Characterization of low phosphorus insensitive mutants reveals a crosstalk between low phosphorus-induced determinate root development and the activation of genes involved in the adaptation of *Arabidopsis* to phosphorus deficiency. *Plant Physiol.* 140 879–889. 10.1104/pp.105.073825 16443695 PMC1400555

[B323] Sarathambal C. Ilamurugu K. (2013). Saline tolerant plant growth promoting diazotrophs from rhizosphere of *Bermuda grass* and their effect on rice. *Indian J. Weed Sci.* 45 80–85.

[B324] Sato T. Ezawa T. Cheng W. Tawaraya K. (2015). Release of acid phosphatase from extraradical hyphae of arbuscular mycorrhizal fungus *Rhizophagus clarus*. *Soil Sci. Plant Nutrition* 61 269–274. 10.1080/00380768.2014.993298 31745622

[B325] Sauer D. Saccone L. Conley D. J. Herrmann L. Sommer M. (2006). Review of methodologies for extracting plant-available and amorphous Si from soils and aquatic sediments. *Biogeochemistry* 80 89–108. 10.1007/s10533-005-5879-3

[B326] Savant N. K. Snyder G. H. Datnoff L. E. (1996). Silicon management and sustainable rice production. *Adv. Agronomy* 58 151–199. 10.1016/s0065-2113(08)60255-2

[B327] Schachtman D. P. Reid R. J. Ayling S. M. (1998). Phosphorus uptake by plants: from soil to cell. *Plant Physiol.* 116 447–453. 10.1104/pp.116.2.447 9490752 PMC1539172

[B328] Schaller J. Faucherre S. Joss H. Obst M. Goeckede M. Planer-Friedrich B. (2019). Silicon increases the phosphorus availability of Arctic soils. *Sci. Rep.* 9:449. 10.1038/s41598-018-37104-6 PMC6345794 30679628

[B329] Scheublin T. R. Sanders I. R. Keel C. Van Der Meer J. R. (2010). Characterisation of microbial communities colonising the hyphal surfaces of arbuscular mycorrhizal fungi. *ISME J.* 4 752–763. 10.1038/ismej.2010.5 20147983

[B330] Schrey S. Hartmann A. Hampp R. (2014). “Rhizosphere interactions,” in *Ecological Biochemistry: Environmental and Interspecies Interactions*, eds Krauss G.-J. Nies D. H. (New Jersey, USA: John Wiley & Sons, Inc.), 292–311. 10.1002/9783527686063.ch15

[B331] Schwertmann U. Fechter H. (1982). The point of zero charge of natural and synthetic ferrihydrites and its relation to adsorbed silicate. *Clay Minerals* 17 471–476. 10.1180/claymin.1982.017.4.10

[B332] Seeling B. Zasoski R. J. (1993). Microbial effects in maintaining organic and inorganic solution phosphorus concentrations in a grassland topsoil. *Plant Soil* 148 277–284. 10.1007/bf00012865

[B333] Sevilla M. Gunapala N. Burris R. Kennedy C. (2001). Comparison of benefit to sugarcane plant growth and 15N2 incorporation following inoculation of sterile plants with *Acetobacter diazotrophicus* wild-type and nif- mutant strains. *Mol. Plant–Microbe Interact.* 14 358–366. 10.1094/mpmi.2001.14.3.358 11277433

[B334] Shahi S. K. Rai A. K. Tyagi M. B. Sinha R. P. Kumar A. (2011). Rhizosphere of rice plants harbor bacteria with multiple plant growth promoting features. *Afr. J. Biotechnol.* 10 8296–8305. 10.5897/ajb11.602

[B335] Shahzad S. M. Khalid A. Arif M. S. Riaz M. Ashraf M. Iqbal Z. (2014). Co-inoculation integrated with P-enriched compost improved nodulation and growth of Chickpea (*Cicer arietinum* L.) under irrigated and rainfed farming systems. *Biol. Fertility Soils* 50 1–12. 10.1007/s00374-013-0826-2

[B336] Sharif M. Claassen N. (2011). Action mechanisms of arbuscular mycorrhizal fungi in phosphorus uptake by *Capsicum annuum* L. *Pedosphere* 21 502–511. 10.1016/s1002-0160(11)60152-5

[B337] Sharma S. B. Sayyed R. Z. Trivedi M. H. Gobi T. A. (2013). Phosphate solubilizing microbes: sustainable approach for managing phosphorus deficiency in agricultural soils. *SpringerPlus* 2:587. 10.1186/2193-1801-2-587 PMC4320215 25674415

[B338] Sheng X. F. He L. Y. (2006). Solubilization of potassium-bearing minerals by a wild-type strain of *Bacillus edaphicus* and its mutants and increased potassium uptake by wheat. *Can. J. Microbiol.* 52 66–72. 10.1139/w05-117 16541160

[B339] Sheng X. F. Zhao F. He L. Y. Qiu G. Chen L. (2008). Isolation and characterization of silicate mineral-solubilizing *Bacillus globisporus* Q12 from the surfaces of weathered feldspar. *Can. J. Microbiol.* 54 1064–1068. 10.1139/w08-089 19096461

[B340] Shenoy V. V. Kalagudi G. M. (2005). Enhancing plant phosphorus use efficiency for sustainable cropping. *Biotechnol. Adv.* 23 501–513. 10.1016/j.biotechadv.2005.01.004 16140488

[B341] Shi-Chu L. Yong J. Ma-Bo L. Wen-Xu Z. Nan X. Hui-Hui Z. (2019). Improving plant growth and alleviating photosynthetic inhibition from salt stress using AMF in alfalfa seedlings. *J. Plant Interact.* 14 482–491. 10.1080/17429145.2019.1662101

[B342] Sigg L. Stumm W. (1981). The interaction of anions and weak acids with the hydrous goethite (α-FeOOH) surface. *Colloids Surf.* 2 101–117. 10.1016/0166-6622(81)80001-7

[B343] Sikes B. A. (2010). When do arbuscular mycorrhizal fungi protect plant roots from pathogens? *Plant Signal. Behav.* 5 763–765. 10.4161/psb.5.6.11776 20400855 PMC3001584

[B344] Singh K. P. Sarkar M. C. (1992). Phosphorus availability in soils as affected by fertilizer phosphorus, sodium silicate and farmyard manure. *J. Ind. Soc. Soil Sci.* 40 762–767.

[B345] Smith S. E. Read D. J. (2008). *Mycorrhizal Symbiosis.* London:Academic Press.

[B346] Smith S. E. Smith F. A. (2011). Roles of arbuscular mycorrhizas in plant nutrition and growth: new paradigms from cellular to ecosystem scales. *Annu. Rev. Plant Biol.* 62 227–250. 10.1146/annurev-arplant-042110-103846 21391813

[B347] Smith S. E. Jakobsen I. Grønlund M. Smith F. A. (2011). Roles of arbuscular mycorrhizas in plant phosphorus nutrition: interactions between pathways of phosphorus uptake in arbuscular mycorrhizal roots have important implications for understanding and manipulating plant phosphorus acquisition. *Plant Physiol.* 156 1050–1057. 10.1104/pp.111.174581 21467213 PMC3135927

[B348] Smyth T. J. Sanchez P. A. (1980). Effects of lime, silicate, and phosphorus applications to an *Oxisol* on phosphorus sorption and ion retention. *Soil Sci. Soc. Am. J.* 44 500–505. 10.2136/sssaj1980.03615995004400030012x

[B349] Sommer M. Kaczorek D. Kuzyakov Y. Breuer J. (2006). Silicon pools and fluxes in soils and landscapes—a review. *J. Plant Nutrition Soil Sci.* 169 310–329. 10.1002/jpln.200521981

[B350] Stamford N. P. Santos P. R. D. Moura A. M. M. F. D. Freitas A. D. S. D. (2003). Biofertilzers with natural phosphate, sulphur and *Acidithiobacillus* in a siol with low available-P. *Sci. Agricola* 60 767–773. 10.1590/s0103-90162003000400024

[B351] Steenhoudt O. Vanderleyden J. (2000). Azospirillum, a free-living nitrogen-fixing bacterium closely associated with grasses: genetic, biochemical and ecological aspects. *FEMS Microbiol. Rev.* 24 487–506. 10.1111/j.1574-6976.2000.tb00552.x 10978548

[B352] Steinkellner S. Lendzemo V. Langer I. Schweiger P. Khaosaad T. Toussaint J. -P. (2007). Flavonoids and strigolactones in root exudates as signals in symbiotic and pathogenic plant-fungus interactions. *Molecules* 12 1290–1306. 10.3390/12071290 17909485 PMC6149470

[B353] Struyf E. Conley D. J. (2009). Silica: an essential nutrient in wetland biogeochemistry. *Front. Ecol. Environ.* 7 88–94. 10.1890/070126

[B354] Struyf E. Smis A. Van Damme S. Garnier J. Govers G. Van Wesemael B. (2010). Historical land use change has lowered terrestrial silica mobilization. *Nat. Commun.* 1:129. 10.1038/ncomms1128 21119642

[B355] Takeda N. Handa Y. Tsuzuki S. Kojima M. Sakakibara H. Kawaguchi M. (2015). Gibberellins interfere with symbiosis signaling and gene expression and alter colonization by arbuscular mycorrhizal fungi in *Lotus japonicus*. *Plant Physiol.* 167 545–557. 10.1104/pp.114.247700 25527715 PMC4326748

[B356] Taktek S. Trépanier M. Servin P. M. St-Arnaud M. Piché Y. Fortin J. A. (2015). Trapping of phosphate solubilizing bacteria on hyphae of the arbuscular mycorrhizal fungus *Rhizophagus irregularis* DAOM 197198. *Soil Biol. Biochem.* 90 1–9. 10.1016/j.soilbio.2015.07.016

[B357] Tarafdar J. C. Marschner H. (1994). Phosphatase activity in the rhizosphere and hyphosphere of VA mycorrhizal wheat supplied with inorganic and organic phosphorus. *Soil Biol. Biochem.* 26 387–395. 10.1016/0038-0717(94)90288-7

[B358] Tarafdar J. C. Yadav R. S. Meena S. C. (2001). Comparative efficiency of acid phosphatase originated from plant and fungal sources. *J. Plant Nutrition Soil Sci.* 164 279–282. 10.1002/1522-2624(200106)164:3<279::aid-jpln279>3.0.co;2-l

[B359] Tatsukami Y. Ueda M. (2016). Rhizobial gibberellin negatively regulates host nodule number. *Sci. Rep.* 6:27998. 10.1038/srep27998 PMC4910070 27307029

[B360] Tawaraya K. (2003). Arbuscular mycorrhizal dependency of different plant species and cultivars. *Soil Sci. Plant Nutrition* 49 655–668. 10.1080/00380768.2003.10410323

[B361] Tawaraya K. Naito M. Wagatsuma T. (2006). Solubilization of insoluble inorganic phosphate by hyphal exudates of arbuscular mycorrhizal fungi. *J. Plant Nutrition* 29 657–665. 10.1080/01904160600564428

[B362] Tipping E. (1981). The adsorption of aquatic humic substances by iron oxides. *Geochimica et Cosmochimica Acta* 45 191–199. 10.1016/0016-7037(81)90162-9

[B363] Tisserant E. Kohler A. Dozolme-Seddas P. Balestrini R. Benabdellah K. Colard A. (2012). The transcriptome of the arbuscular mycorrhizal fungus Glomus intraradices (DAOM 197198) reveals functional tradeoffs in an obligate symbiont. *New Phytol.* 193 755–769. 10.1111/j.1469-8137.2011.03948.x 22092242

[B364] Tisserant E. Malbreil M. Kuo A. Kohler A. Symeonidi A. Balestrini R. (2013). Genome of an arbuscular mycorrhizal fungus provides insight into the oldest plant symbiosis. *Proc. Natl. Acad. Sci. U S A.* 110 20117–20122. 24277808 10.1073/pnas.1313452110 PMC3864322

[B365] Tittarelli A. Milla L. Vargas F. Morales A. Neupert C. Meisel L. (2007). Isolation and comparative analysis of the wheat TaPT2 promoter: identification in silico of new putative regulatory motifs conserved between monocots and dicots. *J. Exp. Bot.* 58 2573–2582. 10.1093/jxb/erm123 17562688

[B366] Toljander J. (2006). *Interactions Between Soil Bacteria and Arbuscular Mycorrhizal Fungi.* Disseration Uppsala: Sveriges lantbruksuniv

[B367] Toljander J. F. Artursson V. Paul L. R. Jansson J. K. Finlay R. D. (2006). Attachment of different soil bacteria to arbuscular mycorrhizal fungal extraradical hyphae is determined by hyphal vitality and fungal species. *FEMS Microbiol. Lett.* 254 34–40. 10.1111/j.1574-6968.2005.00003.x 16451176

[B368] Toljander J. F. Lindahl B. D. Paul L. R. Elfstrand M. Finlay R. D. (2007). Influence of arbuscular mycorrhizal mycelial exudates on soil bacterial growth and community structure. *FEMS Microbiol. Ecol.* 61 295–304. 10.1111/j.1574-6941.2007.00337.x 17535297

[B369] Toro M. Azcon R. Barea J. (1997). Improvement of arbuscular mycorrhiza development by inoculation of soil with phosphate-solubilizing rhizobacteria to improve rock phosphate bioavailability ((sup32) P) and nutrient cycling. *Appl. Environ. Microbiol.* 63 4408–4412. 10.1128/aem.63.11.4408-4412.1997 16535730 PMC1389286

[B370] Toro M. Azcón R. Barea J. M. (1998). The use of isotopic dilution techniques to evaluate the interactive effects of Rhizobium genotype, mycorrhizal fungi, phosphate-solubilizing rhizobacteria and rock phosphate on nitrogen and phosphorus acquisition by *Medicago sativa*. *New Phytol.* 138 265–273. 10.1046/j.1469-8137.1998.00108.x 33863097

[B371] Treder W. Cieslinski G. (2005). Effect of silicon application on cadmium uptake and distribution in strawberry plants grown on contaminated soils. *J. Plant Nutrition* 28 917–929. 10.1081/pln-200058877

[B372] Trolove S. Hedley M. Kirk G. Bolan N. Loganathan P. (2003). Progress in selected areas of rhizosphere research on P acquisition. *Soil Res.* 41 471–499. 10.1071/sr02130

[B373] Turrini A. Avio L. Giovannetti M. Agnolucci M. (2018). Functional complementarity of arbuscular mycorrhizal fungi and associated microbiota: the challenge of translational research. *Front. Plant Sci.* 9:1407. 10.3389/fpls.2018.01407 30319670 PMC6166391

[B374] Uhde-Stone C. Zinn K. E. Ramirez-Yáñez M. Li A. Vance C. P. Allan D. L. (2003). Nylon filter arrays reveal differential gene expression in proteoid roots of white lupin in response to phosphorus deficiency. *Plant Physiol.* 131 1064–1079. 10.1104/pp.102.016881 12644659 PMC166872

[B375] Umamaheswari T. Srimeena N. Vasanthi N. Cibichakravarthy B. Anthoniraj S. Karthikeyan S. (2016). Silica as biologically transmutated source for bacterial growth similar to carbon. *Matt. Arch.* 2:e201511000005.

[B376] Uroz S. Calvaruso C. Turpault M. -P. Frey-Klett P. (2009). Mineral weathering by bacteria: ecology, actors and mechanisms. *Trends Microbiol.* 17 378–387. 10.1016/j.tim.2009.05.004 19660952

[B377] Urrutia M. M. Beveridge T. J. (1994). Formation of fine grained silicate minerals and metal precipitates by a bacterial surface and the implications on the global cycling of silicon. *Chem. Geol.* 116 261–280. 10.1016/0009-2541(94)90018-3

[B378] Vance C. P. Uhde-Stone C. Allan D. L. (2003). Phosphorus acquisition and use: critical adaptations by plants for securing a nonrenewable resource. *New Phytol.* 157 423–447. 10.1046/j.1469-8137.2003.00695.x 33873400

[B379] Vasanthi N. Saleena L. M. Anthoni A. R. (2013). Evaluation of media for isolation and screening of silicate solubilising bacteria. *Int. J. Curr. Res.* 5 406–408.

[B380] Vasanthi N. Saleena L. M. Raj S. A. (2018). Silica solubilization potential of certain bacterial species in the presence of different silicate minerals. *Silicon* 10 267–275. 10.1007/s12633-016-9438-4

[B381] Vassilev N. Vassileva M. Nikolaeva I. (2006). Simultaneous P-solubilizing and biocontrol activity of microorganisms: potentials and future trends. *Appl. Microbiol. Biotechnol.* 71 137–144. 10.1007/s00253-006-0380-z 16544140

[B382] Veneklaas E. J. Lambers H. Bragg J. Finnegan P. M. Lovelock C. E. Plaxton W. C. (2012). Opportunities for improving phosphorus-use efficiency in crop plants. *New Phytol.* 195 306–320. 10.1111/j.1469-8137.2012.04190.x 22691045

[B383] Veresoglou S. D. Chen B. Rillig M. C. (2012). Arbuscular mycorrhiza and soil nitrogen cycling. *Soil Biol. Biochem.* 46 53–62. 10.1016/j.soilbio.2011.11.018

[B384] Veresoglou S. D. Sen R. Mamolos A. P. Veresoglou D. S. (2011). Plant species identity and arbuscular mycorrhizal status modulate potential nitrification rates in nitrogen-limited grassland soils. *J. Ecol.* 99 1339–1349. 10.1111/j.1365-2745.2011.01863.x

[B385] Vierheilig H. (2004). Regulatory mechanisms during the plant arbuscular mycorrhizal fungus interaction. *Can. J. Botany* 82 1166–1176. 10.1139/b04-01533356898

[B386] Villegas J. Fortin J. A. (2001). Phosphorus solubilization and pH changes as a result of the interactions between soil bacteria and arbuscular mycorrhizal fungi on a medium containing NH4+ as nitrogen source. *Can. J. Botany* 79 865–870. 10.1139/b01-06933356898

[B387] Wainwright M. Al-Wajeeh K. Wickramasinghe N. C. Narlikar J. V. (2003). Did silicon aid in the establishment of the first bacterium? *Int. J. Astrobiol.* 2 227–229. 10.1017/s1473550403001587

[B388] Wainwright M. Barakah F. Al-Turk I. Ali T. A. (1991). Oligotrophic micro-organisms in industry, medicine and the environment. *Sci. Prog.* 75, 313–322. 1842853

[B389] Waksman S. A. Starkey R. L. (1924). Microbiological analysis of soil as an index of soil fertility: VII. carbon dioxide evolution1. Soil Science 17 141–162. 10.1097/00010694-192402000-00004

[B390] Wang B. Qiu Y. L. (2006). Phylogenetic distribution and evolution of mycorrhizas in land plants. *Mycorrhiza* 16 299–363. 10.1007/s00572-005-0033-6 16845554

[B391] Wang F. Shi N. Jiang R. Zhang F. Feng G. (2016). In situ stable isotope probing of phosphate-solubilizing bacteria in the hyphosphere. *J. Exp. Botany* 67 1689–1701. 10.1093/jxb/erv561 26802172 PMC4783358

[B392] Wang L. Cai K. Chen Y. Wang G. (2013). Silicon-mediated tomato resistance against *Ralstonia solanacearum* is associated with modification of soil microbial community structure and activity. *Biol. Trace Elem. Res.* 152 275–283. 10.1007/s12011-013-9611-1 23371799

[B393] Wang R. R. Wang Q. He L. Y. Qiu G. Sheng X. F. (2015). Isolation and the interaction between a mineral-weathering *Rhizobium tropici* Q34 and silicate minerals. *World J. Microbiol. Biotechnol.* 31 747–753. 10.1007/s11274-015-1827-0 25716616

[B394] Wang Y. Zhang W. Liu W. Ahammed G. J. Wen W. Guo S. (2021). Auxin is involved in arbuscular mycorrhizal fungi-promoted tomato growth and NADP-malic enzymes expression in continuous cropping substrates. *BMC Plant Biol.* 21:48. 10.1186/s12870-020-02817-2 33461504 PMC7814736

[B395] Whitelaw M. A. (1999). Growth promotion of plants inoculated with phosphate-solubilizing fungi. *Adv. Agronomy* 69 99–151. 10.1016/s0065-2113(08)60948-7

[B396] Whitelaw M. A. Harden T. J. Helyar K. R. (1999). Phosphate solubilisation in solution culture by the soil fungus *Penicillium radicum*. *Soil Biol. Biochem.* 31 655–665. 10.1016/s0038-0717(98)00130-8

[B397] Widdig M. Schleuss P. M. Weig A. R. Guhr A. Biederman L. A. Borer E. T. (2019). Nitrogen and phosphorus additions alter the abundance of phosphorus-solubilizing bacteria and phosphatase activity in grassland soils. *Front. Environ. Sci.* 7:185. 10.3389/fenvs.2019.00185

[B398] Wright D. P. Read D. J. Scholes J. D. (1998). Mycorrhizal sink strength influences whole plant carbon balance of *Trifolium repens* L. *Plant Cell Environ.* 21 881–891. 10.1046/j.1365-3040.1998.00351.x

[B399] Wu L. Jacobson A. D. Hausner M. (2008). Characterization of elemental release during microbe–granite interactions at T= 28 C. *Geochimica et Cosmochimica Acta* 72 1076–1095. 10.1016/j.gca.2007.11.025

[B400] Wu Q. -S. Xia R. -X. (2006). Arbuscular mycorrhizal fungi influence growth, osmotic adjustment and photosynthesis of citrus under well-watered and water stress conditions. *J. Plant Physiol.* 163 417–425. 10.1016/j.jplph.2005.04.024 16455355

[B401] Xiao B. Sun Y. -F. Lian B. Chen T. -M. (2016). Complete genome sequence and comparative genome analysis of the *Paenibacillus mucilaginosus* K02. *Microb. Pathog.* 93 194–203. 10.1016/j.micpath.2016.01.016 26802523

[B402] Xun F. Xie B. Liu S. Guo C. (2015). Effect of plant growth-promoting bacteria (PGPR) and arbuscular mycorrhizal fungi (AMF) inoculation on oats in saline-alkali soil contaminated by petroleum to enhance phytoremediation. *Environ. Sci. Pollut. Res.* 22 598–608. 10.1007/s11356-014-3396-4 25091168

[B403] Yang X. Post W. M. (2011). Phosphorus transformations as a function of pedogenesis: a synthesis of soil phosphorus data using Hedley fractionation method. *Biogeosciences* 8 2907–2916. 10.5194/bg-8-2907-2011

[B404] Yao Q. Li X. Feng G. Christie P. (2001). Mobilization of sparingly soluble inorganic phosphates by the external mycelium of an abuscular mycorrhizal fungus. *Plant Soil* 230 279–285.

[B405] Ye M. Song Y. Long J. Wang R. Baerson S. R. Pan Z. (2013). Priming of jasmonate-mediated antiherbivore defense responses in rice by silicon. *Proc. Natl. Acad. Sci. U S A.* 110 E3631–E3639. 24003150 10.1073/pnas.1305848110 PMC3780902

[B406] Yost R. S. Fox R. L. (1982). Influence of mycorrhizae on the mineral contents of cowpea and soybean grown in an Oxisol 1. *Agronomy J.* 74 475–481. 10.2134/agronj1982.00021962007400030018x

[B407] Zarei M. Saleh-Rastin N. Alikhani H. A. Aliasgharzadeh N. (2006). Responses of lentil to co-inoculation with phosphate-solubilizing rhizobial strains and arbuscular mycorrhizal fungi. *J. Plant Nutrition* 29 1509–1522. 10.1080/01904160600837667

[B408] Zhang L. Ding X. Chen S. He X. Zhang F. Feng G. (2014a). Reducing carbon: phosphorus ratio can enhance microbial phytin mineralization and lessen competition with maize for phosphorus. *J. Plant Interact.* 9 850–856. 10.1080/17429145.2014.977831

[B409] Zhang L. Fan J. Ding X. He X. Zhang F. Feng G. (2014b). Hyphosphere interactions between an arbuscular mycorrhizal fungus and a phosphate solubilizing bacterium promote phytate mineralization in soil. *Soil Biol. Biochem.* 74 177–183. 10.1016/j.soilbio.2014.03.004

[B410] Zhang L. Feng G. Declerck S. (2018a). Signal beyond nutrient, fructose, exuded by an arbuscular mycorrhizal fungus triggers phytate mineralization by a phosphate solubilizing bacterium. *ISME J.* 12 2339–2351. 10.1038/s41396-018-0171-4 29899507 PMC6155042

[B411] Zhang L. Shi N. Fan J. Wang F. George T. S. Feng G. (2018b). Arbuscular mycorrhizal fungi stimulate organic phosphate mobilization associated with changing bacterial community structure under field conditions. *Environ. Microbiol.* 20 2639–2651. 10.1111/1462-2920.14289 29901256

[B412] Zhang L. Xu M. Liu Y. Zhang F. Hodge A. Feng G. (2016). Carbon and phosphorus exchange may enable cooperation between an arbuscular mycorrhizal fungus and a phosphate-solubilizing bacterium. *New Phytol.* 210 1022–1032. 10.1111/nph.13838 27074400

[B413] Zhu X. Q. Wang C. Y. Chen H. Tang M. (2014). Effects of arbuscular mycorrhizal fungi on photosynthesis, carbon content, and calorific value of black locust seedlings. *Photosynthetica* 52 247–252. 10.1007/s11099-014-0031-z

